# Novel Agents on the Therapeutic Horizon For Paediatric Inflammatory Bowel Diseases: An Analysis of Clinical Trials Registries

**DOI:** 10.1007/s40272-026-00766-1

**Published:** 2026-08-13

**Authors:** Dhruv Gupte, Nidhi Rashmikant Suthar, John K. MacDonald, Jessica Le, Ruben J. Colman, Brian G. Feagan, Camilyn Cheng, Jurij Hanzel, Christopher Ma, Vipul Jairath, Eileen Crowley

**Affiliations:** 1 Department of Paediatrics, McMaster University, Hamilton, ON Canada; 2 Division of Paediatric Gastroenterology, Hepatology and Nutrition, Department of Paediatrics, London Health Sciences Centre, Children’s Hospital Western Ontario, Western University, London, ON Canada; 3 Alimentiv Inc., London, ON Canada; 4 Division of Pediatric Gastroenterology, Hepatology and Nutrition, Department of Pediatrics, Stanford University School of Medicine, Stanford, CA USA; 5 Division of Gastroenterology, Department of Medicine, Schulich School of Medicine, Western University, London, ON Canada; 6 Department of Epidemiology and Biostatistics, Western University, London, ON Canada; 7 Department of Gastroenterology and Hepatology, University Medical Center Ljubljana, University of Ljubljana, Ljubljana, Slovenia; 8 Division of Gastroenterology and Hepatology, Department of Medicine, University of Calgary, Calgary, AB Canada; 9 Department of Community Health Sciences, Cumming School of Medicine, University of Calgary, Calgary, AB Canada

## Abstract

**Background and Aims:**

Despite considerable advancements in the therapeutic landscape of inflammatory bowel disease (IBD) for adults, long delays exist for paediatric IBD (pIBD) approval, with a median delay of >7 years following adult approval. Our aim is to summarise the landscape of pIBD clinical trials through a review of trial registries.

**Methods:**

We conducted a cross-sectional review of ClinicalTrials.gov and ClinicalTrialsRegister.eu to identify investigational and approved therapeutic agents for the treatment of pIBD. All interventional studies involving pIBD (aged <18 years) from database inception to July 2, 2026, were considered for inclusion.

**Results:**

Of 3941 records screened, 123 completed trials (77 randomised controlled trials [RCTs] and 46 non-RCTs) and 90 active trials (55 RCTs and 35 non-RCTs) met the inclusion criteria. A majority of completed trials (71%) recruited mixed adult and paediatric populations, whereas nearly half of the active trials recruited exclusively paediatric patients, with most studies enrolling participants aged 2–17 years. Biologics were assessed in 81 trials (42 completed and 39 active), including the approved agents (*n* = 39), infliximab, adalimumab, golimumab, and ustekinumab, and investigational therapies (*n* = 42) such as vedolizumab, guselkumab, mirikizumab, risankizumab, duvakitug, afimkibart, and tulisokibart. Nine completed and 11 active trials assess small molecule therapies, including tofacitinib, upadacitinib, filgotinib, etrasimod, ozanimod, and obefazimod.

**Conclusions:**

Efforts to expedite approval of new agents in pIBD are warranted to ensure timely access to effective medications. Consideration for novel trial designs alongside continued engagement with regulatory bodies, sponsors, and the academic pIBD community is essential to advance drug approvals for pIBD.

**Supplementary Information:**

The online version contains supplementary material available at https://doi.org/10.1007/s40272-026-00766-1.

## Key Points

**Table:** 

Long delays exist for paediatric inflammatory bowel disease (pIBD) drug approval.
A total of 123 completed pIBD clinical trials were identified, with 23 trials (19%) focusing on approved biologics, 19 trials (15%) focusing on investigational biologics, and 9 trials (7%) examining small molecule agents.
A further 90 trials in progress were identified, with 16 trials (18%) exploring the use of approved biologics, 23 trials (26%) focusing on investigational biologics, and 11 trials (12%) examining small molecule therapies.
Adopting novel trial designs and fostering collaboration among research networks may help expedite paediatric drug development and approvals, enabling timely access to emerging therapies for pIBD.

## Introduction

The therapeutic landscape for inflammatory bowel disease (IBD) has advanced considerably over the past two decades, particularly with the introduction of new targeted therapies, including biologics and small molecules. IBD is the most common chronic gastrointestinal disorder among paediatric patients, with an estimated incidence of 8–9 cases per 100,000 children [1, 2]. Furthermore, the incidence of paediatric IBD (pIBD) is increasing, with the highest proportional rise observed in children under the age of 5 years [3].

In general, there are six categories of approved treatments for adult IBD: 5-aminosalicylates (5-ASA), corticosteroids, conventional broad-spectrum immunosuppressants, antibiotics, biologics, and oral small molecules [4]. Currently, only a limited number of therapies are approved by the European Medicines Agency (EMA) or the US Food and Drug Administration (FDA) for pIBD, including sulfasalazine, balsalazide, mesalamine, prednisone/prednisolone, methylprednisolone, hydrocortisone, budesonide, infliximab, adalimumab, golimumab, and ustekinumab. In recent years, dietary therapy has gained increasing recognition in the management of Crohn’s disease (CD), particularly exclusive enteral nutrition (EEN) and the Crohn’s disease exclusion diet (CDED) [5]. EEN involves the administration of a nutritionally complete formula over a defined period and is often used as a first-line induction therapy in children with mild to moderate CD [6]. The CDED is a whole-food dietary therapy that has emerged as an alternative to EEN, offering a broader range of acceptable food choices and improved tolerability in children with CD [7].

**Fig. 1:**
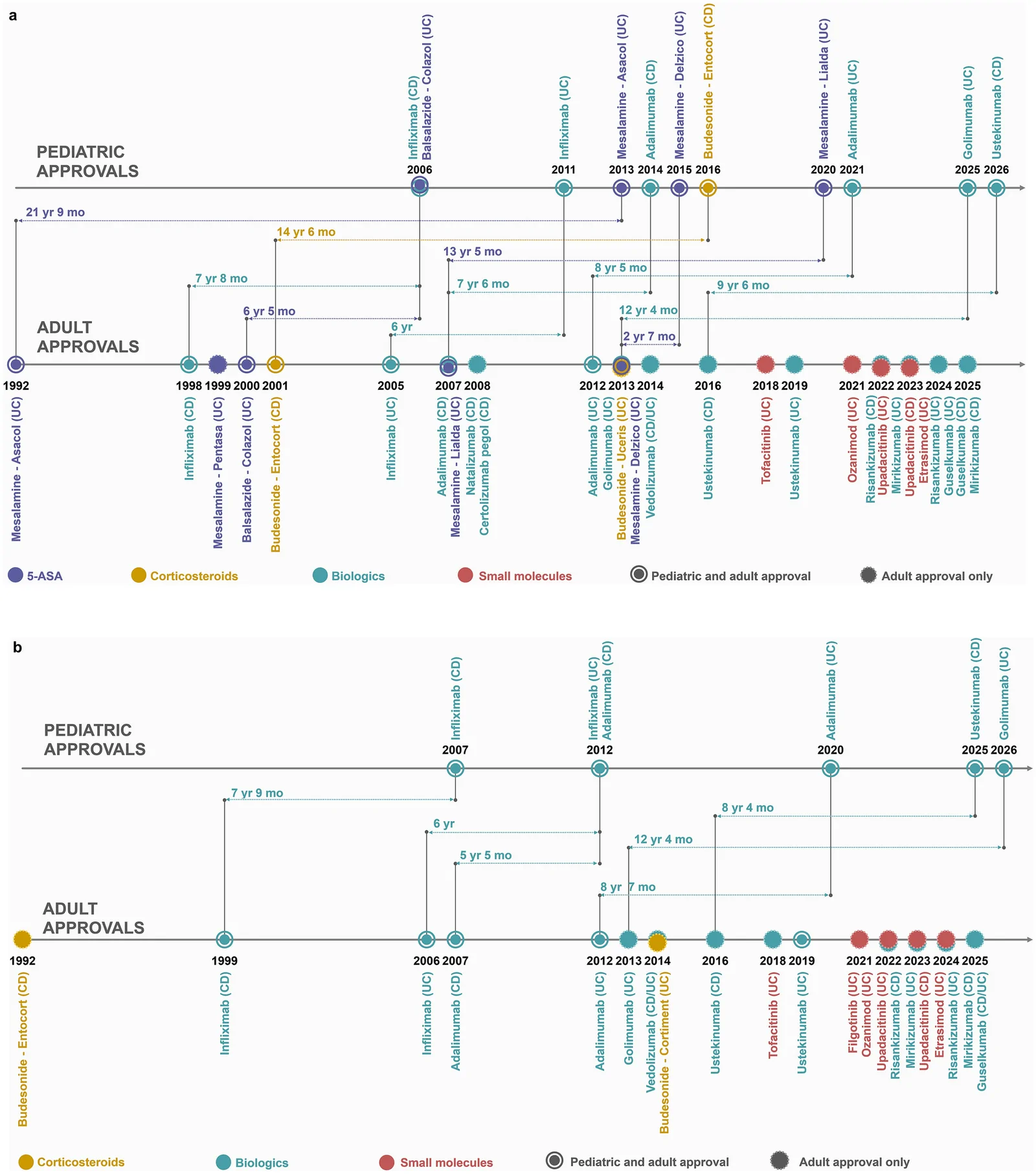
Timeline of FDA (**a**) and EMA (**b**) approved therapies for adult versus paediatric IBD from January 1992 to May 2026. *5-ASA* 5-aminosalicylates, *CD* Crohn’s disease, *EMA* European Medicines Agency, *FDA* Food and Drug Administration, *IBD* inflammatory bowel disease, *mo* months, *UC* ulcerative colitis, *yr* years

Despite recent advances in the therapeutic landscape of IBD, there is a considerable delay in the timing of drug approval between paediatric and adult patients (Fig. 1). A prior review revealed a median delay of 7.5 years (range 6.1–14.5) for the FDA approval of pIBD therapies, and a median delay of 6.8 years (range 5.6–8.6) for the EMA approval relative to the adult approval dates [8]. As a result, ‘off-label’ prescribing is common in paediatric practice, exposing children to drugs without established age-specific safety, efficacy, and dosing data. Several novel drugs targeting various pathways have demonstrated positive results in adult trials, with more agents under development for pIBD [9–11].

Given the recent release of the FDA draft guidance on drug development in pIBD [12], this is an opportune time to improve clinical trial processes for paediatric patients. We aim to provide an overview of the therapeutic landscape in pIBD by reviewing ongoing and completed clinical trials from the ClinicalTrials.gov and ClinicalTrialsRegister.eu registries.

## Methods

We conducted a cross-sectional review of publicly accessible data from ClinicalTrials.gov and ClinicalTrialsRegister.eu to identify all interventional studies for pIBD treatment from database inception to July 2, 2026. The search strategy is reported in the electronic supplementary material (ESM), Supplementary Appendix  1.

Patients under 18 years of age with a confirmed diagnosis of pIBD (CD or ulcerative colitis [UC]) were included. Studies that included adult patients were also considered if they enrolled paediatric patients. All investigational and approved therapies for the treatment of pIBD were considered, including immune-mediated therapies, microbiome-modifying therapies, and nutritional or dietary therapies. Randomised controlled trials (RCTs) and non-randomised (e.g. single-arm) studies were included. Observational, diagnostic, and non-interventional studies were excluded.

Two authors (DG and NRS, JKM, or JL) independently screened the search results based on the inclusion criteria described above and performed independent data extraction. Extracted data included (1) primary and secondary efficacy outcomes, including clinical, endoscopic, histologic, biochemical, pharmacokinetic (PK), pharmacodynamic (PD), transcriptomic, and patient-reported outcomes and composite outcomes; (2) safety outcomes, including occurrence of any adverse events, serious adverse events, or drug discontinuation; (3) trial characteristics, including study drug and its approval status, comparator(s), trial setting (multi- or single-centre), sponsor, trial phases, study design (RCT or non-RCT, open-label, blinded or unblinded), number of target and enrolled participants, recruitment status, treatment phases (induction, maintenance, or induction and maintenance), availability of results and/or publications; and (4) disease and population characteristics, including IBD subtype (CD, UC, or unclassified IBD), disease severity (as measured by the Paediatric Crohn’s Disease Activity Index, the Paediatric Ulcerative Colitis Activity Index, the Physician Global Assessment, the Mayo Clinic Score, or other scales), time to flare(s), and disease duration. All identified interventions were summarised separately for the completed and ongoing trials using their generic names where available. For studies evaluating a specific commercial formulation or variant, both the generic name (where applicable) and the relevant trade name were presented. No automation tools were used. Disagreements during screening or data extraction were resolved by discussion, and if consensus was not reached, a third author (EC) was consulted.

## Results

### Study Selection

**Fig. 2:**
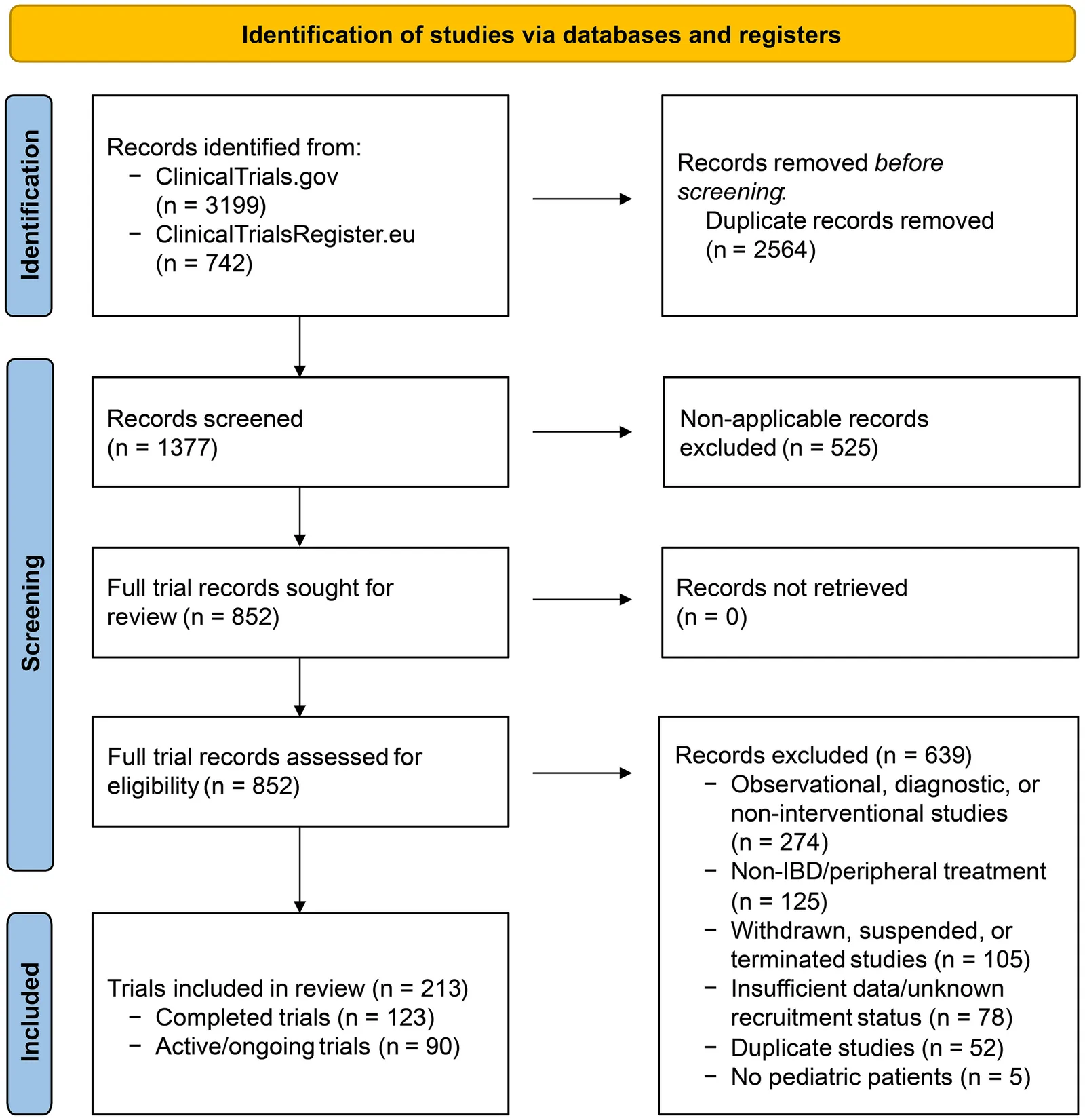
PRISMA flow diagram illustrating the study identification and selection process. ClinicalTrials.gov and ClinicalTrialsRegister.eu were searched from database inception to July 2, 2026, to identify all interventional studies evaluating treatments for paediatric inflammatory bowel disease. *IBD* inflammatory bowel disease

**Fig. 3:**
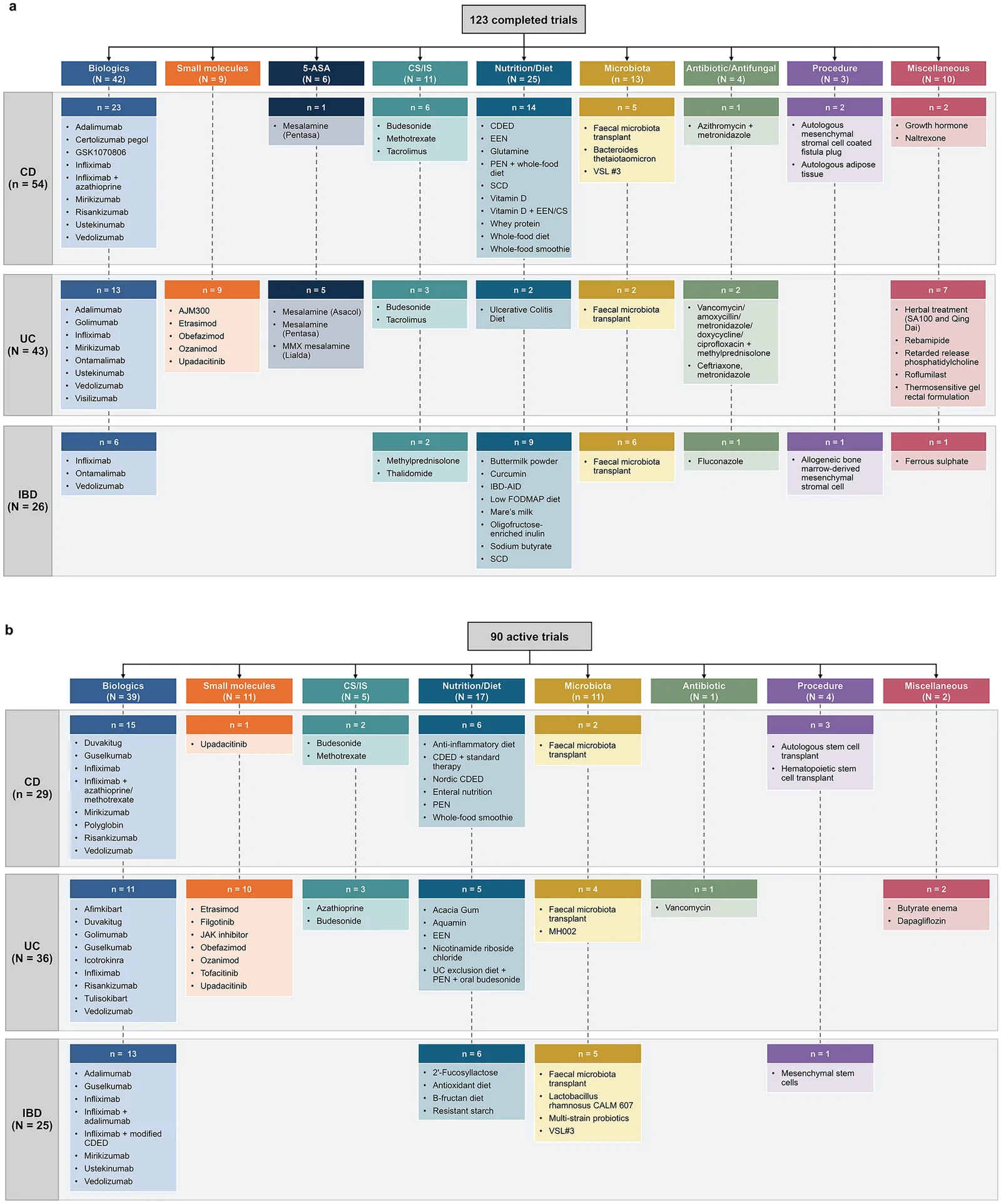
Summary of completed (**a**) and active (**b**) paediatric trials across IBD subtype and intervention category. *5-ASA* 5-aminosalicylates, *CD* Crohn’s disease, *CDED* Crohn’s disease exclusion diet, *CS* corticosteroid, *EEN* exclusive enteral nutrition, *FODMAP* fermentable oligosaccharides, disaccharides, monosaccharides and polyols, *IBD* inflammatory bowel disease, *IBD-AID IBD* anti-inflammatory diet, *IS* immunosuppressant, *JAK* Janus kinase, *PEN* partial enteral nutrition, *SCD* specific carbohydrate diet, *UC* ulcerative colitis

A total of 3941 records were identified from ClinicalTrials.gov (*n* = 3199) and ClinicalTrialsRegister.eu (*n* = 742). After removing duplicates and non-applicable trials, 852 full trial records were assessed for eligibility. A total of 639 trials were excluded with reasons outlined (ESM, Supplementary Table 1), and 213 trials were included in the review (Fig. 2). A summary of completed and active trials, stratified by IBD subtype and intervention category, is provided in Fig. 3. Table 1 provides an overview of recent biologics and small molecules currently under investigation for pIBD.

**Table 1:** Overview of emerging biologic and small-molecule therapies for paediatric inflammatory bowel disease

Class	Therapy	Mechanism of action	Indication	Phase	Age range (years)	Clinical trial identifier	Study status
Anti-α4β7 integrin biologics	Vedolizumab (*n* = 11)	Inhibits α4β7 integrin interaction with MAdCAM-1, preventing lymphocyte trafficking to the intestinal mucosa	CD	III	2–17	NCT04779320	Active
					15–80	NCT02038920	Completed
			UC	III	6–17	NCT06614387	Active
					2–17	NCT04779307	Completed
					15–80	NCT02039505	Completed
			IBD	III	2–17	NCT06405087	Active
						NCT06100289	Active
						NCT05442567	Active
				II	2–17	NCT03138655	Completed
						NCT03196427	Completed
				NA	0–17	NCT02862132	Completed
Anti-IL-23 biologics	Risankizumab (*n* = 7)	Inhibits IL-23 (p19 subunit) and downstream Th17 signalling	CD	III	2–17	NCT05995353	Active
					16–80	NCT03105102	Active
						NCT03104413	Completed
						NCT03105128	Completed
				NA	6–18	NCT06298188	Active
			UC	III	2–17	NCT07071519	Active
					16–80	NCT03398135	Active
	Mirikizumab (*n* = 5)	Inhibits IL-23 (p19 subunit) and downstream Th17 signalling	CD	III	2–17	NCT05509777	Active
					15–80	NCT03926130	Completed
			UC	III	2–17	NCT05784246	Completed
				II	2–17	NCT04004611	Completed
			IBD	III	2–19	NCT04844606	Active
	Guselkumab (*n* = 3)	Inhibits IL-23 (p19 subunit) and downstream Th17 signalling	CD	III	2–17	NCT05923073	Active
			UC	III	2–17	NCT06260163	Active
			IBD	III	≥3	NCT06663332	Active
Anti-IL-12/23 biologics	Ustekinumab (*n* = 5)	Inhibits IL-12/23 (p40 subunit) and downstream Th1/Th17 pathways	CD (approved)	III	2–17	NCT04673357	Completed
				I	2–17	NCT02968108	Completed
			UC	III	2–17	NCT04630028	Completed
			IBD	III	2–17	NCT05092269	Active
				NA	6–17	NCT07529236	Active
Anti-TL1A biologics	Duvakitug (*n* = 4)	Inhibits TL1A signalling via DR3	CD	III	16–80	NCT07184931	Active
						NCT07184944	Active
			UC	III	16–80	NCT07184996	Active
						NCT07185009	Active
	Afimkibart (*n* = 1)	Inhibits TL1A signalling via DR3	UC	III	2–17	NCT07158242	Active
	Tulisokibart (*n* = 1)	Inhibits TL1A signalling via DR3	UC	III	16–80	NCT06052059	Active
JAK inhibitors	Upadacitinib (*n* = 5)	Selective JAK1 inhibitor	CD	III	2–17	NCT06332534	Active
			UC	III	2–17	NCT05782907	Active
					16–75	NCT03006068	Active
						NCT03653026	Completed
				II/III	16–75	NCT02819635	Completed
	Filgotinib (*n* = 1)	Selective JAK1 inhibitor	UC	III	8–17	NCT06865417	Active
	Tofacitinib (*n* = 1)	Pan-JAK inhibitor (predominantly JAK1 and JAK3)	UC	III	2–17	NCT04624230	Active
S1PR modulators	Etrasimod (*n* = 6)	S1PR1 modulation, sequestering lymphocytes in lymph node	UC	III	16–80	NCT03945188	Completed
						NCT03996369	Completed
						NCT04706793	Completed
						NCT03950232	Active
				II	2–11	NCT07470879	Active
					12–17	NCT05287126	Active
	Ozanimod (*n* = 2)	S1PR1 modulation, sequestering lymphocytes in lymph node	UC	III	12–75	EudraCT: 2015-000319-41	Completed
				II/III	2–17	NCT05076175	Active
Micro-RNA modulators	Obefazimod (*n* = 3)	Upregulates microRNA-124, which downregulates pro-inflammatory cytokine production	UC	III	≥16	NCT05507203	Completed
						NCT05507216	Completed
						NCT05535946	Active

n represents the number of completed and active trials

*CD* Crohn’s disease, *DR3* death receptor 3, *IBD* inflammatory bowel disease, *IL* interleukin, *JAK* Janus kinase, *MAdCAM-1* mucosal addressin cell adhesion molecule-1, *NA*  not applicable, *S1PR* sphingosine-1-phosphate receptor, *Th1* T helper 1, *Th17* T helper 17, *TL1A* tumour necrosis factor-like ligand 1A, *UC* ulcerative colitis

### Characteristics of Completed Trials

A total of 123 completed trials (2 trials from ClinicalTrialsRegister.eu and 121 trials from ClinicalTrials.gov) were included (ESM, Supplementary Table 2). There were 77 (63%) RCTs and 46 (37%) non-RCTs. Fifty-four trials (44%) included patients with CD; 43 trials (35%) included patients with UC; and 26 trials (21%) included patients with IBD or unclassified IBD. Seventy-six trials (62%) were multi-centre; and 47 (38%) were single-centre. Most studies (87/123, 71%) recruited both paediatric and adult patients, while 36 studies (29%) included only paediatric patients (0–17 years of age). A total of 41 trials investigated therapies for both induction and maintenance periods; 39 investigated induction phase only; and 14 investigated maintenance phase only. The remaining 29 studies did not report the length of treatment. Comparators included placebo (41/123, 33%), alternate dosing regimens (23/123, 19%), other drugs or treatments (18/123, 15%), and no comparators (40/123, 33%). One study (1%) had both placebo and other drugs/treatments as comparators. For primary outcomes, 30 studies (24%) assessed clinical remission; 13 studies (11%) assessed clinical response; 3 (2%) assessed clinical remission or response; and 19 (15%) assessed other clinical outcomes (e.g. treatment success/failure and changes or improvements in clinical scores). One study (1%) assessed endoscopic outcomes. Thirteen studies (11%) assessed composite or coprimary outcomes, including clinical remission and clinical response, clinical and endoscopic outcomes, clinical and safety outcomes, clinical with biomarker outcomes, and endoscopic with microbiological outcomes. Twenty-five studies (20%) assessed safety/tolerability outcomes; and 19 studies (16%) assessed other outcome measures (e.g. biochemical, histological, PK, microbiological, and patient-reported outcomes).

#### Biologics

Forty-two trials (34%), 27 RCTs and 15 non-RCTs, investigated biologics. Twenty-three of these trials enrolled patients with CD; 13 enrolled patients with UC; and six enrolled patients with IBD. Biologics investigated among studies of CD included anti-tumour necrosis factor (TNF) agents (adalimumab, *n* = 6; certolizumab pegol, *n* = 4; infliximab, *n* = 5; infliximab combined with azathioprine, *n* = 1); anti-interleukin (IL)-18 agent (GSK1070806, *n* = 1); anti-IL-23 agents (mirikizumab, *n* = 1; risankizumab, *n* = 2); anti-IL-12/23 agent (ustekinumab, *n* = 2); and anti-α4β7 integrin agent (vedolizumab, *n* = 1). Biologics investigated among studies of UC included anti-TNF (adalimumab, *n* = 3; golimumab, *n* = 1; infliximab, *n* = 2); anti-IL-23 (mirikizumab, *n* = 2); anti-IL-12/23 (ustekinumab, *n* = 1); anti-mucosal addressin cell adhesion molecule 1 (ontamalimab, *n* = 1); anti-α4β7 integrin (vedolizumab, *n* = 2); and anti-CD3 (visilizumab, *n* = 1). Biologics investigated in studies enrolling both CD and UC included infliximab (*n* = 2), ontamalimab (*n* = 1), and vedolizumab (*n* = 3). Twenty-one trials (14 CD, 4 UC, and 3 IBD) included both paediatric and adult patients. The most common age range for these studies was 16–80 years. Twenty-one trials (9 CD, 9 UC, and 3 IBD) included paediatric patients only. The most common age range for these studies was 6–17 years.

#### Small Molecules

Nine RCTs (7%) investigated small molecules. All of these trials included both paediatric and adult patients with UC. The most common age range was 16–80 years. Small molecules that were investigated in these trials included an α4-integrin antagonist (AJM300, *n* = 1); spingosine-1-phosphate (S1P) receptor modulators (etrasimod, *n* = 3; ozanimod, *n* = 1); microRNA-124 modulator (obefazimod, *n* = 2); and a Janus kinase (JAK) inhibitor (upadacitinib, *n* = 2).

#### 5-Aminosalicylates

Six trials (5%), five RCTs and one non-RCT, investigated mesalamine. Five of these trials evaluated various brands of mesalamine, including Asacol (*n* = 1), Pentasa (*n* = 2), and Lialda (MMX mesalamine, *n* = 2), in patients with UC. One trial evaluated Pentasa sachets in patients with CD. Four trials included paediatric patients with UC, and two trials (1 CD and 1 UC) included both paediatric and adult patients. The most common age range for the paediatric studies was 5–17 years. The common age range for studies that included both adults and children was 6–18 years.

#### Corticosteroids and Immunosuppressants

Eleven trials (9%), five RCTs and six non-RCTs, investigated corticosteroids and immunosuppressants. Six of these trials evaluated budesonide (*n* = 3), methotrexate (*n* = 2), and tacrolimus (*n* = 1) in patients with CD. Three trials evaluated budesonide (*n* = 1) and tacrolimus (*n* = 2) in patients with UC. Two trials evaluated methylprednisolone (*n* = 1) and thalidomide (*n* = 1) in patients with IBD. Two trials included paediatric patients with CD (5–17 years). Nine trials (4 CD, 3 UC, and 2 IBD) included both paediatric and adult patients. The most common age range for studies that included both adults and children was 16–64 years.

#### Nutritional and Dietary Therapies

Twenty-five trials (20%), 15 RCTs and 10 non-RCTs, investigated nutritional and dietary therapies. Five trials evaluated dietary supplements in patients with CD, including glutamine (*n* = 1), vitamin D (*n* = 2), vitamin D combined with EEN or corticosteroids (*n* = 1), and whey protein (*n* = 1). Nine trials evaluated dietary therapies in patients with CD, including CDED (*n* = 2), EEN (*n* = 3), partial enteral nutrition (PEN) combined with a whole-food diet (*n* = 1), specific carbohydrate diet (*n* = 1), whole-food diet (*n* = 1), and whole-food smoothie (*n* = 1). Two trials evaluated UC diet in patients with UC. Nine trials evaluated nutritional or dietary therapies in patients with IBD, including buttermilk powder (*n* = 1), curcumin (*n* = 1), IBD anti-inflammatory diet (IBD-AID, *n* = 1), low fermentable oligosaccharides, disaccharides, monosaccharides and polyols (FODMAP) diet (*n* = 1), mare’s milk (*n* = 1), oligofructose-enriched inulin (*n* = 1), sodium butyrate (*n* = 1), and specific carbohydrate diet (*n* = 2). Twenty-three trials (12 CD, 2 UC, and 9 IBD) included both paediatric and adult patients. The most common age range for these studies was 8–18 years. Two trials included paediatric patients with CD (e.g. aged 1–17 years).

#### Microbiological and Microbiota Interventions

Thirteen trials (11%), six RCTs and seven non-RCTs, investigated microbiological or microbiota interventions. Two trials evaluated faecal microbiota transplant (FMT); one trial evaluated live biotherapeutic product (e.g. *Bacteroides thetaiotaomicron* [Thetanix]); and two trials evaluated a multi-strain probiotic formulation (e.g. VSL#3) in patients with CD. Two trials evaluated FMT in patients with UC and six trials evaluated FMT in patients with IBD. Three trials (1 CD and 2 IBD) included paediatric patients only. The most common age range for the paediatric studies was 3–17 years. Ten trials (4 CD, 2 UC, and 4 IBD) included both paediatric and adult patients. The most common age range for these studies was 7–21 years.

#### Antibiotic and Antifungal Agents

Four RCTs (3%) investigated antibiotics or antifungals. One trial evaluated a combination of azithromycin and metronidazole in patients with CD. One trial evaluated a combination of corticosteroids and antibiotics (e.g. methylprednisolone with oral vancomycin, amoxicillin, metronidazole, doxycycline, or ciprofloxacin); and one trial evaluated ceftriaxone and metronidazole in patients with UC. One trial evaluated fluconazole in patients with IBD. One trial only included paediatric patients with CD (aged 5–17 years). Three trials included both paediatric and adult patients with UC (aged 2–75 years and ≥13 years) and IBD (aged ≥6 years).

#### Procedural Interventions or Tissue Transfer Therapy

Three non-RCTs (2%) investigated procedural interventions or cell/tissue transfer therapy. One trial evaluated autologous mesenchymal stromal cell coated fistula plug and one trial evaluated autologous adipose tissue therapy in patients with CD. One trial evaluated allogeneic bone marrow-derived mesenchymal stromal cell therapy in IBD. One trial included paediatric patients with CD (aged 12–17 years), one trial included children and young adults with IBD (aged 12–22 years), and the other trial included patients of all age groups with CD (0 to ≥65 years of age).

#### Miscellaneous Therapies

Ten trials (8%), six RCTs and four non-RCTs, investigated miscellaneous therapies. One trial evaluated growth hormone and one trial evaluated naltrexone in CD. Seven trials enrolled patients with UC, investigating herbal treatment (SA100, *n* = 1; Qing Dai, *n* = 1), rebamipide (*n* = 1), retarded release phosphatidylcholine (*n* = 2), roflumilast (*n* = 1), and thermosensitive gel rectal formulation (*n* = 1). One trial evaluated ferrous sulphate in patients with IBD. One trial included paediatric patients with CD (aged 6–17 years), and another included paediatric patients with UC (aged 4–17 years). The other eight trials (1 CD, 6 UC, and 1 IBD) included both paediatric and adult patients (e.g. CD: ≥5 years; UC: ≥16 years; IBD: 13–80 years).

### Characteristics of Active Trials

A total of 90 trials (5 trials from ClinicalTrialsRegister.eu and 85 trials from ClinicalTrials.gov) are either in the pre-recruitment phase, actively recruiting paediatric patients with IBD, or have completed recruitment with ongoing follow-up (Supplementary Table 3, see ESM). Fifty-five studies (61%) are RCTs and 35 (39%) are non-RCTs. Twenty-nine studies (32%) include patients with CD; 36 (40%) include patients with UC; and 25 (28%) include patients with IBD. Forty-one studies (46%) recruit exclusively paediatric patients, while 49 studies (54%) enrolled both adult and paediatric patients, with four studies (4%) not specifying the age criteria. Comparators within these studies include placebo (22/90, 24%), other drugs/treatments (23/90, 26%), alternate dosing regimens or other routes of administration (19/90, 21%), and no comparators (24/90, 27%). Two studies (2%) have both placebo and other drugs/treatments as comparators. The median expected number of patients enrolled in these active trials is 100 (interquartile range 50–155).

#### Biologics

Thirty-nine trials (43%), 25 RCTs and 14 non-RCTs, investigate biologics. Fifteen of these trials enrol patients with CD; 11 enrol patients with UC; and 13 enrol patients with IBD. Biologics investigated in studies of CD include anti-TNF (infliximab, *n* = 4; combination of infliximab with azathioprine or methotrexate, *n* = 2); anti-IL-23 (guselkumab, *n* = 1; mirikizumab, *n* = 1; risankizumab, *n* = 3), oral immunoglobulin (Polyglobin, *n* = 1); anti-TNF-like ligand 1A (TL1A) agent (duvakitug, *n* = 2); and anti-α4β7 integrin (vedolizumab, *n* = 1). Biologics investigated in studies of UC include anti-TNF (golimumab, *n* = 1; infliximab, *n* = 1); anti-IL-23 (guselkumab, *n* = 1; risankizumab, *n* = 2); IL-23 receptor antagonist peptide (icotrokinra, *n* = 1); anti-TL1A (afimkibart, *n* = 1; duvakitug, *n* = 2; tulisokibart, *n* = 1); and anti-α4β7 integrin (vedolizumab, *n* = 1). Biologics investigated in studies enrolling both CD and UC include anti-TNF (adalimumab, *n* = 1; infliximab, *n* = 3; combination of adalimumab and infliximab, *n* = 1; and combination of infliximab and modified CDED, *n* = 1); anti-IL-23 (guselkumab, *n* = 1; mirikizumab, *n* = 1); anti-IL-12/23 (ustekinumab, *n* = 2); and anti-α4β7 integrin (vedolizumab, *n* = 3). Twenty-one trials (8 CD, 5 UC, and 8 IBD) enrol paediatric patients only (e.g. aged 2–17 years). Four trials (3 CD and 1 UC) enrol children and adolescents aged 6–18 years. Ten trials (3 CD, 5 UC, and 2 IBD) enrol adolescents and adults (e.g. aged 16–80 years). Four trials (1 CD and 3 IBD) enrol children, adolescents, and adults (e.g. ≥2 years of age).

#### Small Molecules

Eleven trials (12%), four RCTs and seven non-RCTs, investigate small molecules. One trial evaluates upadacitinib in patients with CD. Ten trials enrol patients with UC, including five trials evaluating JAK inhibitors (filgotinib, *n* = 1; tofacitinib, *n* = 1; upadacitinib, *n* = 2), four trials evaluating S1P receptor modulators (etrasimod, *n* = 3; ozanimod, *n* = 1), and one trial evaluating obefazimod. Seven trials (1 CD and 6 UC) enrol paediatric patients only (e.g. aged 2–17 years); and four trials enrol adolescents and adults with UC (e.g. aged 16–80 years).

#### Corticosteroids and Immunosuppressants

Five RCTs (6%) investigate corticosteroids and immunosuppressants. One trial evaluates budesonide and one trial evaluates methotrexate in patients with CD. Two trials evaluate budesonide and one trial evaluates azathioprine in patients with UC. Four trials (1 CD and 3 UC) enrol paediatric patients only (e.g. aged 2–17 years). One trial enrols children and adolescents aged 6–18 years with CD.

#### Nutritional and Dietary Therapies

Seventeen trials (19%), 15 RCTs and two non-RCTs, investigate nutritional and dietary therapies. Six trials evaluate dietary therapies in patients with CD, including catered anti-inflammatory diet (*n* = 1), Nordic CDED (*n* = 1), CDED with standard therapy (*n* = 1), enteral nutrition (*n* = 1), PEN (*n* = 1), and whole-food smoothie (*n* = 1). Three trials evaluate dietary supplements (e.g. Acia gum, Aquamin, and nicotinamide riboside chloride); one trial evaluates UC exclusion diet with PEN and oral budesonide; and one trial evaluates EEN in patients with UC. Three trials evaluate resistant starch, one trial evaluates 2'-fucosyllactose, one trial evaluates antioxidant diet, and one trial evaluates B-fructan diet in patients with IBD. Four trials (1 CD and 3 IBD) enrol paediatric patients only (e.g. 5–17 years). Four trials (2 CD and 2 UC) enrol children and adolescents up to 18 years of age. Four trials (2 CD and 2 UC) enrol adolescents and adults (e.g. 16–75 years). Five trials (1 CD, 1 UC, and 3 IBD) enrol children, adolescents and adults (e.g. 5–80 years).

#### Microbiological and Microbiota Interventions

Eleven trials (12%), four RCTs and seven non-RCTs, investigated microbiological or microbiota interventions. Seven trials (2 CD, 3 UC, and 2 IBD) investigate FMT. Three trials investigate a single-strain probiotic (e.g. Lactobacillus rhamnosus CALM 607 [LR607]) or multi-strain probiotics (e.g.VSL#3) in patients with IBD. One trial assesses live biotherapeutic product (MH002) in patients with UC. Four trials (2 CD and 2 UC) enrol paediatric patients only (e.g. aged 2–16 years). One trial enrols children and adolescents with IBD (e.g. aged 5–18 years). One trial enrols adolescents and adults with UC (e.g. ≥16 years of age). Two trials enrol children, adolescents, and adults (e.g. UC: 10–80 years; IBD: 3–21 years). Three studies did not specify their age eligibility criteria.

#### Antibiotic and Antifungal Agents

One RCT (1%) investigates oral vancomycin in paediatric patients with UC (e.g. aged 6–15 years).

#### Procedural Interventions or Tissue Transfer Therapy

Four non-RCTs (4%) investigate procedural or tissue transfer therapies. Two trials evaluate autologous stem cell transplant and one trial evaluates hematopoietic stem cell transplant in patients with CD. One trial evaluates mesenchymal stem cells in patients with IBD. One trial enrols adolescents and adults with CD (e.g. aged 13–28 years) and two trials enrol children, adolescents and adults with CD (e.g. aged 10–60 years). One study did not publish their age eligibility criteria. No paediatric exclusive populations were investigated during this period of study.

#### Miscellaneous Therapies

Two trials (2%), one RCT and one non-RCT, investigate butyrate enema and dapagliflozin (sodium-glucose cotransporter 2 inhibitor) in patients with UC. One study enrols children and adults (e.g. aged 7–21 years), and the other study enrols children, adolescents, and adults with UC (e.g. 18 months to 60 years of age).

## Discussion

Despite substantial therapeutic advances in adult IBD, delays in drug approval for paediatric patients persist, limiting timely access to effective therapies for children. Prior to 2025, infliximab and adalimumab were the only biologics approved by both the FDA and EMA for pIBD, resulting in the widespread off-label use of other agents without robust paediatric-specific PK, safety, and efficacy data. Recently, golimumab has received approval from both the FDA and EMA for the treatment of paediatric UC, supported by the efficacy, safety, and PK findings from the phase I (NCT01900574) and phase III PURSUIT studies (ClinicalTrials.gov identifier: NCT03596645) [13, 14]. Ustekinumab has also been approved by both the FDA and EMA for the treatment of paediatric CD based on findings from the phase III UNITI Jr study (NCT04673357) [15]. Approval of ustekinumab for the treatment of paediatric UC is currently pending [16].

In this cross-sectional review of the therapeutic landscape in pIBD, we identified 123 completed and 90 active pIBD trials, nearly half of which investigated biologic therapies, highlighting increasing research activity in this area. Among studies assessing biologics, 39 studies (23 completed and 16 active) evaluate approved agents, including infliximab (*n* = 22), adalimumab (*n* = 10), golimumab (*n* = 2), and ustekinumab (*n* = 5). Forty-two studies (19 completed and 23 active) investigate non-approved agents, such as vedolizumab (*n* = 11), risankizumab (*n* = 7), mirikizumab (*n* = 5), guselkumab (*n* = 3), certolizumab pegol (*n* = 4), duvakitug (*n* = 4), afimkibart (*n* = 1), tulisokibart (*n* = 1), GSK1070806 (*n* = 1), icotrokinra (*n* = 1), ontamalimab (*n* = 2), Polyglobin (*n* = 1), and visilizumab (*n* = 1). While several phase IV trials of anti-TNF agents remain ongoing, recent drug development efforts in pIBD have increasingly focused on other investigational therapeutic targets, particularly agents targeting α4β7 integrin, IL-23, and TL1A.

Of the investigational biologics, vedolizumab was the most extensively studied across both completed (*n* = 6) and active trials (*n* = 5). It is commonly used off-label in pIBD, accounting for up to 9% of biologic use in paediatric UC and 6% in paediatric CD [17]. A prospective cohort study (VEDOKIDS, NCT02862132) [18, 19], as well as phase II (HUBBLE, NCT03138655) [20] and phase III RCTs (KEPLER, NCT04779307) [21] have demonstrated favourable efficacy and safety of vedolizumab as both induction and maintenance therapy in pIBD, particularly in children with UC. Both the HUBBLE and KEPLER trials reported comparable remission rates across the weight-adjusted low- versus high-dosing regimens in paediatric patients [21]. Furthermore, the overall safety profile, PK parameters, and clinical response patterns were consistent with those observed in adults in the GEMINI trials [22, 23], supporting the extrapolation of adult data to paediatric patients. Multiple phase III trials (e.g. NCT04779320, NCT05442567, NCT06405087, NCT06100289) are underway to further characterise vedolizumab’s PK, efficacy and long-term safety in pIBD. Findings from these studies may support future approval of vedolizumab for paediatric use.

Trials evaluating anti-IL-23 agents in pIBD, such as risankizumab (e.g. UC: NCT07071519; CD: NCT05995353 and NCT06298188), guselkumab (e.g. UC: NCT06260163; CD: NCT05923073; IBD: NCT06663332) and mirikizumab (e.g. CD: NCT05509777; IBD: NCT04844606) are in progress, with the FDA granting orphan drug designation to risankizumab for paediatric CD [24]. Phase II (SHINE-1, NCT04004611) and phase III (SHINE-2, NCT05784246) trials evaluating mirikizumab in paediatric UC have been completed, with the phase II trial demonstrating favourable efficacy and safety in paediatric UC [25]. More recently, several phase III trials of anti-TL1A agents are being conducted in adolescents and adults (aged 16–80 years) with CD (e.g. duvakitug, NCT07184931 and NCT07184944) and UC (e.g. duvakitug, NCT07184996 and NCT07185009; tulisokibart, NCT06052059). One trial (NCT07158242) is exclusively assessing the PK, safety, and efficacy of afimkibart in children aged 2–17 years with moderately to severely active UC.

In addition to biologics, small molecules represent another emerging treatment option for pIBD, encompassing 7% of all completed trials and 12% of all active trials included in this review. Most studies evaluate JAK inhibitors (*n* = 8), including upadacitinib, tofacitinib, and filgotinib, and S1P receptor modulators (*n* = 8) such as etrasimod and ozanimod. Real-world evidence increasingly supported the effectiveness and safety of tofacitinib and upadacitinib in paediatric UC and CD, including those with refractory disease [26–29]. An international multicentre retrospective study by the Paediatric IBD Porto group of ESPGHAN recently demonstrated promising results for upadacitinib as an induction therapy in both paediatric CD [30] and UC [31, 32]. Although data on S1P receptor modulators in pIBD are limited, studies are underway to evaluate the safety, efficacy, and PK or PD of ozanimod (NCT05076175) and etrasimod (NCT05287126 and NCT07470879) in paediatric UC. Since these small molecules are orally administered, they offer an attractive alternative for children who are intolerant or unresponsive to parenteral biologics and conventional therapies. Although these small molecules are currently used off-label in refractory pIBD, their use in clinical practice is likely to expand as additional safety and efficacy data become available.

Several trends emerged from our study. First, the majority of completed studies (71%) enrolled a mixed adult–paediatric population, with participant ages spanning 0–80 years. Among completed biologic trials, approximately half recruited only paediatric patients, whereas none of the studies evaluating oral small molecules exclusively recruited paediatric patients. Most of these studies enrolled participants aged 16 years or older. The lack of standardisation in age eligibility along with limited representation of younger children greatly reduces the generalisability and real-world applicability of study findings to the pIBD population. Nonetheless, some agents, such as risankizumab and etrasimod, have been approved in the United Kingdom and Israel for patients >16 years of age based on adult RCTs that enrolled adolescents (e.g. aged 16–80 years) [33–35]. Ongoing studies are evaluating these therapies and other biologics (e.g. vedolizumab, mirikizumab, and guselkumab) along with small molecules (e.g. upadacitinib and ozanimod) in paediatric patients aged 2–17 years, indicating a shift toward more paediatric-focused drug development. Additionally, multiple dosing regimens are explored for these investigational agents, which may help inform age-specific dosing strategies and support future approval for paediatric patients.

Regulatory requirements continue to pose challenges for the approval of new drugs in pIBD. Recently, the FDA released a draft guidance outlining several recommendations for drug development in pIBD [12]. Many of these were well accepted by the international pIBD community, including the extrapolation of adult efficacy data, early corticosteroid tapering, shortened washout periods, minimisation of placebo use, and incorporation of Bayesian approaches for analysis [36–38]. However, concerns remain regarding the lack of emphasis on dose-finding (e.g. PK/PD) studies in younger children, the requirement for repetitive ileocolonoscopies, and ethical challenges of testing regimens known to be suboptimal in adults. These barriers may contribute to the delayed access to therapies that have long been established as safe and effective in adult IBD patients.

Efforts to expedite the approval of novel agents for pIBD have been made by international stakeholders, such as the conect4children consortium [39]. Encouraging collaboration among research networks and integrating innovative trial designs may help advance paediatric drug development. A notable example is the phase III MACARONI-23 study, which employs platform trial design to evaluate two IL-23p19 inhibitors, guselkumab (NCT05923073) and mirikizumab (NCT05509777), in paediatric CD [40, 41]. Platform trials allow potential extrapolation of adult efficacy and safety data and facilitate the addition of new interventions such that multiple treatments can be simultaneously or sequentially evaluated over time without a defined end date [40, 41]. Furthermore, they can be adapted in response to accumulating trial data to improve efficiency, save time, and reduce costs [42]. Adopting Bayesian adaptive designs and other master protocols along with validated surrogate endpoints offer further opportunities to streamline pIBD trials while generating high quality and interpretable data for regulatory authorities [40]. A landmark ESPGHAN-NASPGHAN position paper recently outlined several strategies to accelerate pIBD drug development, including extrapolation of adult efficacy and safety data to children >2 years of age and PK data to children >12 years of age and weighing ≥30–40 kg; enrolment of adolescents (≥12 years) in adult trials to support dosing and PK modelling in younger children; enabling adolescents to access therapies approved for adults; limiting full colonoscopies in paediatric trials by replacing with non-invasive biomarkers or imaging; and leveraging existing real-world evidence and registries to support drug approval in pIBD [35].

Extrapolation from adult trials to facilitate paediatric approval has been supported in rheumatology. For instance, upadacitinib was approved by the FDA for the treatment of active polyarticular juvenile idiopathic arthritis (pJIA) and psoriatic arthritis (PsA) in children >2 years of age based on PK-matching efficacy extrapolation from adult patients with rheumatoid arthritis or PsA [43]. Similarly, the approval of guselkumab for paediatric plaque psoriasis (PsO) and PsA was supported by findings from the phase III PROTOSTAR study in paediatric PsO and PK extrapolation from adult studies VOYAGE 1 and 2 (PsO) and DISCOVER-1 and 2 (PsA) [44]. These extrapolation approaches may be applied to expedite drug approval in pIBD. Although children with IBD often experience more extensive disease and a greater disease burden than adults, the underlying pathophysiology and treatment responses are largely similar between paediatric (>2 years) and adult patients [35, 45]. Nevertheless, given the physiological and PK differences between younger children (<12 years) and adults, including the non-linear relationship between body weight and drug clearance [46], extrapolation of adult efficacy and safety data alone may be insufficient.

Turner and colleagues have proposed an extrapolation framework for drug development in pIBD [35]. Specifically, they recommend extrapolating efficacy and safety data from adults or adolescents to children aged 2–12 years or weighing <30–40 kg, while emphasising the need for at least one open-label, single-arm PK study to establish dosing regimen and validate dose-exposure relationship in this population. Interim safety data may be extrapolated from adult studies or evaluated in paediatric trials, while long-term safety data should be collected through large post-authorisation real‐world registries. Extrapolation is not recommended for infantile-onset (<2 years) or monogenic IBD due to their distinct pathogenic mechanisms relative to paediatric- and adult-onset IBD.

We acknowledge several limitations of this study. First, our search was restricted to two major clinical trial registries (ClinicalTrials.gov and ClinicalTrialsRegister.eu) which were established in the early 2000s. As a result, studies conducted prior to the launch of these platforms may not have been captured. However, the aim of this review was to characterise the contemporary therapeutic landscape in pIBD, with a focus on recent research trends. Second, although we sought comprehensive identification of pIBD trials, registry data are often incomplete or inconsistently updated. Trials with unknown, withdrawn, or suspended statuses, as well as those with insufficient information, could not be fully characterised and were therefore excluded. Third, substantial heterogeneity in study design, age eligibility criteria, and outcome definitions limited direct comparisons across trials. Lastly, our study provides a cross-sectional overview of the current pIBD pipeline rather than an exhaustive assessment of all emerging therapies. Given the rapid growth of novel IL-23 inhibitors, JAK inhibitors, and S1P receptor modulators, recently initiated studies or trial status changes may not have been fully captured due to delays in reporting.

## Conclusion

Despite major advances in the therapeutic landscape for adult IBD, paediatric patients continue to experience significant delays in accessing newly approved therapies. Our review highlights increasing research activity in pIBD, particularly with targeted biologics and oral small molecules. Emerging data for vedolizumab, IL-23 inhibitors, JAK inhibitors, and S1P receptor modulators suggest that several of these agents may soon become available for paediatric use. Continued adoption of modern trial designs and strengthened collaboration among research networks will be essential to ensure timely access to effective therapies and improve outcomes for pIBD.

## Supplementary Information

Below is the link to the electronic supplementary material.

Supplementary file1 (PDF 1688 KB)

## Declarations

### Availability of Data and Materials

All relevant data are included within the article and its supplementary material.
