# Supplementary material for: Novel Agents on the Therapeutic Horizon For Paediatric Inflammatory Bowel Diseases: An Analysis of Clinical Trials Registries
Source: Paediatr Drugs. 2026 Aug 13;28(5):547–61. doi: 10.1007/s40272-026-00766-1 (PMC13624012; doi:10.1007/s40272-026-00766-1)
Supplement: Supplementary file 1 [file 40272_2026_766_MOESM1_ESM.pdf]

Supplemental Material for:
Novel agents on the therapeutic horizon for paediatric inflammatory bowel diseases: A review of clinical trials registries

Journal: Pediatric Drugs

Authors: Dhruv Gupte[1,2], Nidhi Rashmikant Suthar[2], John K MacDonald[3], Jessica Le[3], Ruben J Colman[4], Brian G Feagan[3,5,6,†], Camilyn Cheng[2], Jurij Hanzel[3,7], Christopher Ma,[3,8,9] Vipul Jairath,[3,5,6] Eileen Crowley[2,3]

Affiliations:
[1]Department of Paediatrics, McMaster University, Hamilton, ON, Canada.
[2]Division of Paediatric Gastroenterology, Hepatology and Nutrition, Department of Paediatrics, Children's Hospital Western Ontario, Western University, London Health Sciences Centre, London, ON, Canada.
[3]Alimentiv Inc., London, ON, Canada.
[4] Division of Pediatric Gastroenterology, Hepatology and Nutrition, Department of Pediatrics, Stanford University School of Medicine, Stanford, California, USA.
[5]Department of Medicine, Division of Gastroenterology, Schulich School of Medicine, Western University.
[6]Department of Epidemiology and Biostatistics, Western University, London, ON, Canada.
[7]Department of Gastroenterology and Hepatology, University Medical Center Ljubljana, University of Ljubljana, Ljubljana, Slovenia.
[8]Division of Gastroenterology and Hepatology, Department of Medicine, University of Calgary, Calgary, AB, Canada.
[9]Department of Community Health Sciences, Cumming School of Medicine, University of Calgary, Calgary, AB, Canada.
[†]Deceased.

Correspondence: Eileen Crowley, MBChBAO, MRCPI, MSc, Assistant Professor Department of Pediatrics, Division of Pediatric Gastroenterology, Western University, London, Ontario, Canada. Email: eileen.crowley@lhsc.on.ca

Table of Contents

Supplementary Appendix 1: Search Strategy 3
Supplementary Table 1a: Excluded Studies – ClinicalTrials.gov (n=572)..4
Supplementary Table 1b: Excluded Studies – ClinicalTrialsRegister.eu (n=67)......................................20
Supplementary Table 2: Characteristics of Completed Trials in Paediatric IBD 24
Supplementary Table 3: Characteristics of Active/Ongoing Trials in Paediatric IBD 49

Supplementary Appendix 1: Search Strategy

The search was run without language restriction. The following key words were used to extract relevant studies from the databases.

ClinicalTrials.gov (Search dates: April 10, 2023/ May 13, 2024/ July 22, 2025/ July 2, 2026)

Pediatric and inflammatory bowel diseases

Paediatric and inflammatory bowel diseases

Pediatric and Crohn

Paediatric and Crohn

Pediatric and ulcerative colitis

Paediatric and ulcerative colitis

Child* and inflammatory bowel diseases

Child* and Crohn

Child* and ulcerative colitis

Adolescent and inflammatory bowel diseases

Adolescent and Crohn

Adolescent and ulcerative colitis

ClinicalTrialsRegister.eu (Search dates: April 10, 2023/ May 13, 2024/ July 22, 2025/ July 2, 2026)

Pediatric and inflammatory bowel diseases

Paediatric and inflammatory bowel diseases

Pediatric and Crohn

Paediatric and Crohn

Pediatric and ulcerative colitis

Paediatric and ulcerative colitis

Child* and inflammatory bowel diseases

Child* and Crohn

Child* and ulcerative colitis

Adolescent and inflammatory bowel diseases

Adolescent and Crohn

Adolescent and ulcerative colitis

Supplementary Table 1a: Excluded Studies – ClinicalTrials.gov (n=572)

No.#	NCT Number	Title (short)	Reason for exclusion
1.	NCT00001184	Immune Regulation in Ulcerative Colitis or Crohn's Disease	Observational study
2.	NCT00061282	Clotrimazole Enemas for Pouchitis in Children and Adults	Terminated
3.	NCT00061737	Quality of Life in Pediatric Inflammatory Bowel Disease	Observational study
4.	NCT00086554	Collection of Specimens for Study of Inflammatory Bowel Disease	Observational study
5.	NCT00098111	Imuran (Azathioprine) Dose-Ranging Study in Crohn's Disease"	Terminated
6.	NCT00113503	Imuran Dosing in Crohns Disease Study"	Terminated
7.	NCT00114803	Nasal Calcitonin in the Treatment of Bone Mineral Loss in Children and Adolescents With Inflammatory Bowel Disease	Non-IBD/peripheral treatment
8.	NCT00152620	Dexamethasone Versus Methylprednisolone for the Treatment of Active Inflammatory Bowel Disease	Terminated
9.	NCT00179842	Immune Ablation and Stem Cell Support for Crohns Disease"	Withdrawn
10.	NCT00206661	Safety and Efficacy Study in the Treatment of Pediatric Crohns Disease"	Terminated
11.	NCT00207688	A Long Term Safety Study of Infliximab (Remicade) in in Ulcerative Colitis Patients	Observational study
12.	NCT00215020	Evaluation of Urinary Isoprostanes in the Assessment of Children With Inflammatory Bowel Disease	Observational study
13.	NCT00237055	Infectious Agents in Pediatric Crohns"	Observational study
14.	NCT00257699	Study of Antibiotics in the Treatment of Colonic Crohn's Disease	Terminated
15.	NCT00259571	Retarded Phosphatidylcholine Versus Mesalazin in Remission of Ulcerative Colitis.	Withdrawn
16.	NCT00261976	A Long-term Safety Study of Infliximab (Remicade)	Observational study
17.	NCT00265772	Comparison of a Nutritional Anti-Inflammatory Treatment to Steroids for Pediatric Crohns Disease - the Molecular Basis	Unknown status
18.	NCT00275418	Beta Carotene From Natural Source for Patients With Non-Active Crohn's Disease	Unknown status
19.	NCT00277251	Alendronate Osteoporosis Study	Non-IBD/peripheral treatment
20.	NCT00278577	Hematopoietic Stem Cell Support in Patients With Severe Crohns Disease	Terminated
21.	NCT00286689	Effects of Growth Hormone in Chronically Ill Children	Withdrawn
22.	NCT00289328	Glucocorticoid-induced Osteopenia in Children	Observational study
23.	NCT00291681	Differences in Human Germinal Center B Cell Selection Revealed by Analysis of IgVH Gene Hypermutation and Lineage Trees in Inflammatory Bowel Disease	Unknown status
24.	NCT00317369	A Dose-finding Study of OPC-6535 in Patients With Active Crohn's Disease	Terminated
25.	NCT00340444	Genetic Studies of Inflammatory Bowel Disease	Observational study
26.	NCT00349388	Safety and Compliance of Taking Mesalamine Once a Day in Pediatric Patients	Terminated
27.	NCT00353639	Telomere Repair Gene Mutation in Inflammatory Bowel Disease	Observational study
28.	NCT00360022	Transition of Inflammatory Bowel Disease (IBD) Patients From Pediatric to Adult Gastroenterologist (GI)	Terminated
29.	NCT00364130	Bone Health in Pediatric Crohns Disease: A Low Magnitude Mechanical Stimulus Trial	Non-IBD/peripheral treatment
30.	NCT00367705	VSL#3 Treatment in Children With Crohn's Disease	Unknown status
31.	NCT00392951	Sirolimus for Autoimmune Disease of Blood Cells	Non-IBD/peripheral treatment
32.	NCT00434551	MACCS: Multicenter Australian CE in Patients With Suspected Crohns Disease Study"	Observational study
33.	NCT00440817	An Observational Study of Lymphoma in Patients With Rheumatoid Arthritis or Crohns Disease.	Observational study
34.	NCT00446238	Treatment of Depressed Adolescents With Physical Illness	Non-IBD/peripheral treatment
35.	NCT00462072	Centocor Microarray Study of Patients	Non-IBD/peripheral treatment
36.	NCT00463619	Follow-up Study With Retarded Release Phosphatidylcholine in Ulcerative Colitis	Observational study
37.	NCT00472992	Pregnancy Exposure Registry for TysabriÂ®	Observational study
38.	NCT00487396	Evaluation of Capsule Endoscopy in Patients With Suspected Crohns Disease"	Diagnostic study
39.	NCT00495521	High Dose Oral 4-Aminosalicylic Acid (PASERÂ®) to Control Acute Flares of Mild to Moderate Crohns Disease in Children"	Terminated
40.	NCT00511329	Growth Hormone in Children With Juvenile Rheumatoid Arthritis (JRA) and With Crohns Disease"	Terminated
41.	NCT00542776	Impact of Immunosuppression in IBD Patients on Response to Influenza Vaccine	Observational study

No.#	NCT Number	Title (short)	Reason for exclusion
42.	NCT00553163	Hypnotherapy for Prevention of Relapse in Ulcerative Colitis: a Randomised, Single-blind, Controlled Clinical Trial	Terminated
43.	NCT00573846	Phenotypic Characteristics of Inflammatory Bowel Disease in Southeast Asian Population - A Multi-centered US Study	Withdrawn
44.	NCT00586599	Levels of the Stat4 Alpha and Stat4 Beta Isoforms in PBMCs From Patients With Crohns Disease and Ulcerative Colitis"	Non-IBD/peripheral treatment
45.	NCT00586807	Metabolic Response to Infliximab in Pediatric Ulcerative Colitis	Terminated
46.	NCT00606346	A Multicenter, Prospective, Long-term, Observational Registry of Pediatric Patients With Inflammatory Bowel Disease	Observational study
47.	NCT00609752	Adverse Effects of Glucocorticoid Therapy on Bone in Childhood Crohns Disease"	Unknown status
48.	NCT00621257	Vitamin D Levels in Children With IBD	Terminated
49.	NCT00711945	Growth Relapse and Outcomes With Therapy 1	Observational study
50.	NCT00723905	Remicade Infusion Management Program	Observational study
51.	NCT00727636	Immunogenicity to Human Papillomavirus Vaccine (Gardasil) Among IBD Patients on Immunosuppressive Therapy	Non-IBD/peripheral treatment
52.	NCT00748410	Study to Evaluate the Pharmacodynamics of SB-656933 in Patients With Ulcerative Colitis	Terminated
53.	NCT00755937	RemicadeÂ® Crohn's Disease Registry Across Canada (Study P02793)	Observational study
54.	NCT00764699	Effect of IncrelexÂ® on Children With Crohn Disease	Terminated
55.	NCT00769353	Understanding and Treating Neuropsychiatric Symptoms of Pediatric Physical Illness	Non-IBD/peripheral treatment
56.	NCT00780949	Immunological Consequences of CARD15/NOD2 Mutations in Crohns Disease"	Non-interventional
57.	NCT00783575	Genetic Markers as Predictors of Phenotypes in Pediatric Onset Crohns Disease	Withdrawn
58.	NCT00790543	Risk Stratification of Rapid Disease Progression in Children With Crohns Disease	Observational study
59.	NCT00798473	Zoledronate for Osteopenia in Pediatric Crohns	Non-IBD/peripheral treatment
60.	NCT00815061	Epidemiology of Ulcerative Colitis and Crohns Disease in Non Jewish Israel Population"	Withdrawn
61.	NCT00833170	Pediatric Inflammatory Bowel Disease Collaborative Research Group Registry	Observational study
62.	NCT00895336	Lactobacillus GG in Pediatric Ulcerative Colitis (UC)	Withdrawn
63.	NCT00897312	Effect of Biological Therapy on Biomarkers in Patients With Untreated Hepatitis C, Metastatic Melanoma, or Crohn Disease	Terminated
64.	NCT00899678	The Use of Certolizumab Pegol for Treatment of Active Crohns Disease in Children and Adolescents"	Withdrawn
65.	NCT00915044	Interactions Between Immune Cells of Intestinal Mucosa or Peripheral Blood With the Extracellular Matrix in Inflammatory Bowel Disease (IBD)	Observational study
66.	NCT00946361	Impaired Insulin-like Growth Factor-1 (IGF-1) Generation Causes Protein Catabolism and Poor Growth in Children With Crohn Disease	Terminated
67.	NCT00964210	Protecting Young Special Risk Females From Cervical Cancer Through Human Papilloma Virus (HPV) Vaccination	Non-IBD/peripheral treatment
68.	NCT00972088	Prevalence of Inflammatory Bowel Disease in Patients With Perianal Disease	Diagnostic study
69.	NCT00979485	Microparticles in Pediatric Inflammatory Disease	Terminated
70.	NCT01000272	Respiratory Tract Inflammation in Children With Inflammatory Bowel Disease (IBD)	Observational study
71.	NCT01004185	Assessing the Safety/Efficacy of AsacolÂ® Given Every 12 Hours to Children and Adolescents for the Maintenance of Remission of Ulcerative Colitis	Terminated
72.	NCT01019460	A Comparative Study of Capsule Endoscopy, Magnetic Resonance Imaging and Computer Tomography Scanning of the Small Bowel in Crohn's Disease	Diagnostic study
73.	NCT01026480	Ileal Pouch-Anal Anastomosis Registry	Terminated
74.	NCT01032525	Monroe County Inflammatory Bowel Disease (IBD) Registry	Observational study
75.	NCT01034358	Immune Response to the Human Papillomavirus Vaccine in Young Women With Inflammatory Bowel Disease	Non-IBD/peripheral treatment
76.	NCT01046773	Vitamin D Supplementation as Non-toxic Immunomodulation in Children With Crohns Disease"	Terminated
77.	NCT01086059	OTIS Autoimmune Diseases in Pregnancy Project	Observational study
78.	NCT01088646	Evaluation of PillCam® Express Capsule Endoscopy Delivery System	Diagnostic study
79.	NCT01099111	Advanced Metagenomic Analysis of Human Colonic Microbiota in Patients With Chronic GI Disorders	Observational study
80.	NCT01169194	Multi-Center African-American Inflammatory Bowel Disease Study (MAAIS)	Observational study
81.	NCT01169207	Johns Hopkins Crohns Disease and Ulcerative Colitis Study	Observational study

No.#	NCT Number	Title (short)	Reason for exclusion
82.	NCT01171872	Gene Expression in Inflammatory Bowel Disease	Observational study
83.	NCT01241058	Registry of Pediatric Patients Diagnosed With Crohns Disease in Belgium	Observational study
84.	NCT01253733	MD2Me - Texting to Promote Chronic Disease Management	Non-interventional
85.	NCT01277419	German Spondyloarthritis Inception Cohort	Observational study
86.	NCT01297907	Bronchial Hyperreactivity in Children With Crohn Disease	Observational study
87.	NCT01298648	Special Investigation in Patients With Crohn's Disease (All Patients Investigation)	Observational study
88.	NCT01392365	Development of a Prediction Model for Differential Diagnosis Between Intestinal Tuberculosis and Crohns Disease	Observational study
89.	NCT01438151	Understanding the Relationship Between Infliximab Levels to Clinical Response of Remicade in Crohn's Disease	Terminated
90.	NCT01438372	IV Iron Sucrose vs Oral FeSO4 in Treating IDA in Pediatric IBD	Withdrawn
91.	NCT01464333	Special Investigation (Long-term Investigation) in Patients With Crohn's Disease	Observational study
92.	NCT01468090	Disease Course in an IBD Cohort in the Era of Biological Treatment	Observational study
93.	NCT01490528	Durability and Tolerability of Infliximab in Pediatric Inflammatory Bowel Disease Patients: A 10 Year Single Center Experience	Observational study
94.	NCT01548014	The Effect of a Probiotic Preparation (VSL#3) Plus Infliximab in Children With Crohns Disease"	Unknown status
95.	NCT01551563	Stavanger University Hospital Inflammatory Bowel Disease Trial	Observational study
96.	NCT01554007	Clinical Course of Korean Crohn's Disease Cohort	Observational study
97.	NCT01555437	Assessment of Capsule Endoscopy Scoring Index (CESI), Harvey-Bradshaw Index (HBI) and Biological Markers in Small Bowel Crohn's Disease (SBCD)	Observational study
98.	NCT01559142	Efficacy and Safety of Two Regimens of Maintenance Therapy in Children With Crohn Disease	Unknown status
99.	NCT01590043	Identifying Saliva Markers in Inflammatory Bowel Disease	Unknown status
100.	NCT01593462	Comparative Effectiveness of MR Enterography	Diagnostic study
101.	NCT01604564	Registry With Information About Colitis Ulcerosa and Familial Adenomatous Polyposis Patients	Observational study
102.	NCT01605175	Predictors of Mucosal Healing in Inflammatory Bowel Disease (IBD) Patients Treated With Anti-TNF	Observational study
103.	NCT01605188	Predictors of Non-response and Loss of Response in IBD Patients Treated With Anti-TNF	Observational study
104.	NCT01632462	A Prospective, Placebo Controlled, Double-Blind, Cross-over Study on the Effects of a Probiotic Preparation (VSL#3) on Metabolic Profile, Intestinal Permeability, Microbiota, Cytokines and Chemokines Expression and Other Inflammatory Markers in Pediatric Patients With Crohns Disease"	Unknown status
105.	NCT01647412	Growth Hormone and Exclusion Diet Therapy in Juvenile Crohns Disease"	Withdrawn
106.	NCT01671579	Comparison of Bowel Ultrasound & MR Enterography in the Follow-up of Previously Diagnosed Pediatric Small Bowel Crohn Disease	Terminated
107.	NCT01731665	Epidemiology of Inflammatory Bowel Disease in the Songpa-Kangdong District, Seoul, Korea.	Observational study
108.	NCT01740349	Evaluation Of The Pillcam Colon Capsule Endoscopy (Pcce-2) In Pediatric Ulcerative Colitis	Diagnostic study
109.	NCT01749813	Specific Carbohydrate Diet as Maintenance Therapy in Crohns Disease	Observational study
110.	NCT01752790	Efficacy and Safety of Top-down Therapy in Pediatric Crohns Disease	Withdrawn
111.	NCT01781481	The Pediatric Intermed: A New Clinical Decision Making Tool	Observational study
112.	NCT01793831	Standardized Fecal Microbiota Transplantation for Crohn’s Diseases	Observational study
113.	NCT01797224	OTIS Autoimmune Diseases in Pregnancy Project	Observational study
114.	NCT01798953	Surgical Reconstruction in Ulcerative Colitis With Primary Sclerosing Cholangitis	Observational study
115.	NCT01802593	Comparison of 2 Immunomodulator Withdrawal Schemes for Infliximab Monotherapy in Active Pediatric Crohns Disease After Thiopurine Failure	Terminated
116.	NCT01804166	A Research Study to Bank Samples for Future Evaluation to Identify Biomarkers That Predispose Crohns Disease and Ulcerative Colitis Patients to Develop Hepatosplenic T-Cell Lymphoma (HSTCL)"	Non-IBD/peripheral treatment
117.	NCT01854242	Study of the Relationship Between Glycogen Storage Disease Type Ia and Inflammatory Bowel Disease	Observational study
118.	NCT01861288	Deep Endoscopic Remission Assessed by a Surrogate Biomarker in Patients With Inflammatory Bowel Disease	Observational study
119.	NCT01880307	Infliximab Top-down in Pediatric Crohn	Terminated
120.	NCT01881490	The ImageKids Study: Developing the pMEDIC and the PICMI	Diagnostic study

No.#	NCT Number	Title (short)	Reason for exclusion
121.	NCT01896986	HPV Vaccination in Special Risk Groups: 5 Year Follow-up	Observational study
122.	NCT01918813	Risk of Methicillin-resistant S.Aureus (MRSA) Infections in MRSA Carrier Under Introduction of Rapid MRSA Screening	Non-IBD/peripheral treatment
123.	NCT01932658	Autologous Hematopoietic Stem Cell Transplantation for Crohn's Disease Treatment	Withdrawn
124.	NCT01947036	T and B Cell Responses in Autoimmune Diseases	Terminated
125.	NCT01947816	Special Investigation in Patients With Ulcerative Colitis	Observational study
126.	NCT01961492	Fecal Microbiota Transplantation in Patients With Ulcerative Colitis	Withdrawn
127.	NCT01966744	Self-Management Assistance for Recommended Treatment (SMART) Portal	Observational study
128.	NCT01971970	Biomarkers of Anti-TNF Treatment in Inflammatory Bowel Disease (IBD)	Observational study
129.	NCT01974869	Anti Tumor Necrosis Factor Alpha Agents and Surgical Stress Response	Observational study
130.	NCT02003859	Predictive Value of pCLE Findings in Pediatric IBD	Observational study
131.	NCT02009202	Preparation Prior to Colonoscopy in Children	Diagnostic study
132.	NCT02020382	Evaluation of the Diagnostic Value of microRNAs for Inflammatory Bowel Diseases	Diagnostic study
133.	NCT02027727	Individualized Infiximab Dosing-Proof of Concept Study	Unknown status
134.	NCT02057016	Long-term Scheduled Therapy With Infliximab in Inflammatory Bowel Disease	Observational study
135.	NCT02061163	Contrast-Enhanced Ultrasound in Human Crohns Disease	Withdrawn
136.	NCT02065089	Development of Emma--a Pediatric IBD Quiz	Non-IBD/peripheral treatment
137.	NCT02066272	Safety of Anti-tumor Necrosis Factor (TNF) Monoclonal Antibodies in Inflammatory Bowel Disease	Unknown status
138.	NCT02076750	Weekly Vitamin D in Pediatric IBD	Non-IBD/peripheral treatment
139.	NCT02079441	Immune Function in Offsprings of Females With Inflammatory Bowel Disease (IBD) Treated With Anti-TNF Medications During Pregnancy	Unknown status
140.	NCT02103361	Stelara and Tremfya Pregnancy Exposure Registry OTIS Autoimmune Diseases in Pregnancy Project	Observational study
141.	NCT02129972	Colon Capsule Endoscopy in Children	Diagnostic study
142.	NCT02130362	A Non-Interventional Clinical Study to Evaluate Long-Term Safety and Effectiveness of HUMIRA (Adalimumab) in Pediatric Patients With Moderately to Severely Active Crohns Disease (CD)	Observational study
143.	NCT02144129	Effects of WB-EMS in Ulcerative Colitis Patients	Withdrawn
144.	NCT02152241	Long-term Functional Outcome of Children With Inflammatory Bowel Disease	No pediatric patients
145.	NCT02161640	Vascular Dysfunction in Paediatric IBD	Observational study
146.	NCT02162862	Treating Disrupted Sleep in Individuals With Inflammatory Bowel Disease	Observational study
147.	NCT02164877	Effect of Soluble Dietary Fiber on Bacterial Translocation in Crohns Disease	Terminated
148.	NCT02181153	Genetic, Serological Fecal and Clinical Markers in Siblings of Children With Inflammatory Bowel Disease	Non-IBD/peripheral treatment
149.	NCT02182947	Capsule Endoscopy in Inflammatory Bowel Disease (IBD) in Children	Diagnostic study
150.	NCT02193685	Identification Genetic, Immunologic and Microbial Markers of Hirschsprung Associated Enterocolitis in Children With Hirschsprung Disease	Observational study
151.	NCT02197780	Head-to-head Comparison of Two Fecal Biomarkers to Screen Children for IBD	Observational study
152.	NCT02199626	SECOND GENERATION COLON CAPSULE ENDOSCOPY (CCE-2) IN PEDIATRIC CROHNS DISEASE (CD).	Diagnostic study
153.	NCT02217722	Use of the Ulcerative Colitis Diet for Induction of Remission	Terminated
154.	NCT02225795	Pilot Study of Stem Cell Transplantation for Children and Young Adults With Refractory Crohns Disease.	Withdrawn
155.	NCT02229344	Study of Clinical Course of Newly Diagnosed Moderate to Severe Ulcerative Colitis in Korea	Observational study
156.	NCT02247258	Azathioprine in the Prevention of Ileal Crohns Disease Postoperative Recurrence."	Terminated
157.	NCT02256605	Vitamin D3 Supplementation in Pediatric IBD: Weely vs Daily Dosing Regimens	Observational study
158.	NCT02263703	Immunogenicity of HPV Vaccine in Immunosuppressed Children	Non-IBD/peripheral treatment
159.	NCT02272868	Fecal Microbial Transplant in Pediatric Crohns Disease	Terminated
160.	NCT02277223	Curcumin in Pediatric Ulcerative Colitis	Withdrawn
161.	NCT02281799	Thiopurine Induced Pancreatitis in IBD Patients	Withdrawn
162.	NCT02291523	The Effect of Therapeutic Fecal Transplant on the Gut Microbiome in Children With Ulcerative Colitis	Unknown status
163.	NCT02308917	The Canadian Children Inflammatory Bowel Disease Network (CIDsCaNN)	Observational study

No.#	NCT Number	Title (short)	Reason for exclusion
164.	NCT02319798	Telephone Consultation as a Substitute for Routine Out-patient Face-to-face Consultation for Children With Inflammatory Bowel Disease	Non-IBD/peripheral treatment
165.	NCT02326155	To Evaluate the Safety and Efficacy of Remsimaâ„¢ in Patients With Crohns Disease (CD) or Ulcerative Colitis (UC)	Terminated
166.	NCT02330458	The Role of Small Bowel Ultrasound in Initiation of Infliximab in Crohns Disease Patients"	Unknown status
167.	NCT02332356	Therapeutic Optimization Study Based on MR Enterocolonography in Patients With Crohns Disease"	Unknown status
168.	NCT02335281	Standardized Fecal Microbiota Transplantation for Inflammatory Bowel Disease	Unknown status
169.	NCT02341248	Bacteria & Inflammation in the Gut (BIG) Study	Unknown status
170.	NCT02341742	Physical Activity and Bone Mineral Density in Children and Adolescents With Chronic Inflammatory Bowel Disease	Observational study
171.	NCT02379117	Eating Behaviour in Crohn's Disease	Observational study
172.	NCT02392221	Impact of Therapeutic Strategies in the Pediatric Inflammatory Bowel Disease: a Population Based Study (1988-2011).	Observational study
173.	NCT02426762	Quick Skin Sealant in Closure of Surgical Wound After Laparoscopic Surgery	Unknown status
174.	NCT02439372	Assessment of CMV-specific ELISPOT Assay for Predicting CMV Colitis in Patient With Ulcerative Colitis	Non-IBD/peripheral treatment
175.	NCT02453880	Web-based CBT for Symptoms of Mild-to-Moderate Anxiety and Depression in Youth With Chronic Illness	Non-IBD/peripheral treatment
176.	NCT02470663	Bone Density in Children With IBD Treated With Amorphous Calcium or Commercial Crystalline Calcium	Withdrawn
177.	NCT02517684	Top-down Infliximab Study in Kids With Crohns Disease"	Duplicate study
178.	NCT02521064	Effects of Exclusive Enteral Nutrition on the Microbiome in Pediatric Inflammatory Bowel Disease	Observational study
179.	NCT02560727	To Evaluate the Feasibility, Safety, and Efficacy of TET for FMT Via Colonic Approach	Observational study
180.	NCT02566889	An Efficacy and Safety Study of Infliximab Dose Escalation in Pediatric Participants With Inflammatory Bowel Disease	Terminated
181.	NCT02578238	Post-Marketing Surveillance of Humira in Korean Pediatric Crohns Disease (CD) Patients Under the \New-Drug Re-examination\"	Observational study
182.	NCT02578576	The Diagnostic Methods of Early Postoperative Flare-up of Crohn's Disease	Unknown status
183.	NCT02588222	GRINCH: Groningen Initiative on Reference Intervals in Children	Observational study
184.	NCT02614911	Characterization of Phenotype and Genotype of Early Onset Enteropathies	Observational study
185.	NCT02629211	Initial Experience With a Novel Single-Balloon Enteroscopy System in Children With IBD	Diagnostic study
186.	NCT02641392	A Long-term Active Treatment Study of Mongersen (GED-0301) in Subjects With Crohn's Disease	Terminated
187.	NCT02676622	Autologous Stem Cell Transplant for Refractory Crohns Disease	Withdrawn
188.	NCT02694042	Mission is RemissionÂ®: How Can a Disease Self-management Website Change Care?	Terminated
189.	NCT02704026	Nutritional Status and Body Composition in Childhood Inflammatory Bowel Disease	Observational study
190.	NCT02707289	Retrospective Collection of PillCam SB Movies	Observational study
191.	NCT02727959	Inflammatory Bowel Disease in South Eastern Norway	Observational study
192.	NCT02734589	Fecal Transplantation Using a Diet for Donor and Recipient in Refractory Colitis	Terminated
193.	NCT02740309	Integrated Behavioral Health Innovations in Childhood Chronic Illness Care Delivery Systems	Withdrawn
194.	NCT02745457	Body Composition in Children With Inflammatory Bowel Disease	Observational study
195.	NCT02772458	Reduced Appetite in Crohn's Disease: The Role of the Brain in the Control of Food Intake	Non-IBD/peripheral treatment
196.	NCT02799615	Anti-TNF Therapy for Refractory Colitis in Hospitalized Children	Observational study
197.	NCT02822495	Expanded Access Protocol for Tabelecleucel for Patients With Epstein-Barr Virus-Associated Viremia or Malignancies	Unknown status
198.	NCT02826330	Abnormal Fecal Microbiota in Healthy Subjects at High Risk for Crohn's Disease	Observational study
199.	NCT02839317	COmparison of MicroBiota AccordIng to Age in Crohns Disease (COMeBACk)"	Observational study
200.	NCT02842450	Study of Photographs of Interest in Training to Obtain Good Reproducibility of the Diagnosis of Perianal Lesions of Crohn's Disease Inspection in Internal Gastroenterology	Observational study
201.	NCT02847884	IDeaL Pilot Study - Infliximab Dose to Level: Pilot Study	Observational study

No.#	NCT Number	Title (short)	Reason for exclusion
202.	NCT02851134	Search for New Genetic Mutations Major Effect in Crohn's Disease	Observational study
203.	NCT02852694	Reduce Risk for Crohns Disease Patients	Unknown status
204.	NCT02874365	Intestinal Stem Cells Characterization	Non-interventional
205.	NCT02896530	Arterial Thrombosis and Inflammatory Bowel Disease: a Series of 6 Cases and Literature Review	Observational study
206.	NCT02904590	A Prospective Study on the Incidence and Risk Factors Related to Infection in Patients With Inflammatory Bowel Disease	Observational study
207.	NCT02909244	Study of Gut Microbiota in Primary Immune Deficiency, Possibly Associated With Inflammatory Bowel Disease	Observational study
208.	NCT02925338	National Observational Study On The Use Of Inflectraâ„¢ An Infliximab Biosimilar In Real Life	Observational study
209.	NCT02929706	Pre-genotype NUDT 15 R139C on Reducing Thiopurine-induced Leucopenia in Inflammatory Bowel Disease	Unknown status
210.	NCT02930564	The Challenge Study: A Dietary Personalization Protocol for Patients With Crohns Disease and Deep Remission	Terminated
211.	NCT02942511	Predictors Favoring the Role of Exclusive Enteral Nutrition in Active Crohn's Disease	Terminated
212.	NCT02946203	Comparison of VoLumen and Breeza Oral Contrast Agents in Pediatric Patients	Observational study
213.	NCT02970149	Identifying a Sleep Screener for Disease Relapse in Children With Inflammatory Bowel Disease	Unknown status
214.	NCT02974322	A Study of Efficacy and Safety of Mongersen (GED-0301) for the Treatment of Adult and Adolescent Subjects With Active Crohns Disease	Withdrawn
215.	NCT02984059	Study Assessing Risk Factors for Abdominal Pain in Children With Inflammatory Bowel Disease	Observational study
216.	NCT03014219	Phase 1 Crohns Pediatric Sub-study of MSC AFP"	Withdrawn
217.	NCT03017014	A Study to Assess Safety and Effectiveness of Adalimumab for Treating Children and Adolescents With Crohns Disease in Real Life Conditions	Terminated
218.	NCT03026582	Intestinal Ultrasound in Pediatric Inflammatory Bowel Disease	Observational study
219.	NCT03051204	OptiCal Study -Optimizing Fecal Calprotectin Monitoring: a Clinical Study Comparing CalproLab Against PhiCal and Evaluating Its Association With the Gut Microbiome	Unknown status
220.	NCT03059186	A Gratitude Intervention in Improving Well-being and Coping in People Living With Inflammatory Bowel Disease	Non-IBD/peripheral treatment
221.	NCT03088449	Personalising Anti-TNF Therapy in Crohns Disease (PANTS)	Unknown status
222.	NCT03097731	Epidemiological Screening of IL10RA Mutation Rate in China	Withdrawn
223.	NCT03131414	The IMAGINE-SPOR CIHR Chronic Disease Network	Observational study
224.	NCT03134508	Population Based Evaluation of the Use of TNFÎ± Antagonists in Pregnant Women With Inflammatory Bowel Diseases	Observational study
225.	NCT03134586	Advanced Non-Invasive Diagnostics in Inflammatory Bowel Disease	Non-IBD/peripheral treatment
226.	NCT03143517	Fecal Calprotectin Collection Protocol	Observational study
227.	NCT03148028	Immunological Characteristics of Patients With PID and IBD	Unknown status
228.	NCT03170622	Evaluation of Faecal Volatile Organic Compounds in the Diagnosis of Paediatric Inflammatory Bowel Disease	Observational study
229.	NCT03171246	CD-TREAT Diet: a Novel Therapy for Active Luminal Crohns Disease"	Unknown status
230.	NCT03172000	Detection of Helicobacter Pylori in Ulcerative Colitis Patients	Unknown status
231.	NCT03176875	Comparison of Partial and Exclusive Enteral Nutrition in the Treatment of Active Childhood-onset Crohns Disease"	Unknown status
232.	NCT03179865	Evaluation of Early Mucosal Healing During Clinical Remission in Pediatric Ulcerative Colitis	Observational study
233.	NCT03204136	Tacrolimus Versus Methotrexate as Rescue Therapy for Refractory Inflammatory Bowel Disease	Observational study
234.	NCT03209232	Infliximab Accelerated Induction in Moderate to Severe Pediatric UC	Withdrawn
235.	NCT03221166	Thalidomide Versus Infliximab in New Onset Crohns Disease With Poor Prognostic Factors	Terminated
236.	NCT03228615	IBD Shared Decision Making Intervention	Non-IBD/peripheral treatment
237.	NCT03251118	A 5-year Longitudinal Observational Study of Patients Undergoing Therapy for Inflammatory Bowel Disease	Observational study

No.#	NCT Number	Title (short)	Reason for exclusion
238.	NCT03259308	Efficacy and Safety Study of SHP647 as Induction Therapy in Participants With Moderate to Severe Ulcerative Colitis	Terminated
239.	NCT03259334	Efficacy and Safety Study of SHP647 as Induction Therapy in Participants With Moderate to Severe Ulcerative Colitis	Terminated
240.	NCT03269916	Fertility and Ovarian Reserve Function in the Patient With Inflammatory Bowel Disease	Unknown status
241.	NCT03270267	Chronic Inflammatory Bowel Diseases (IBD) and School Absenteeism	Observational study
242.	NCT03277703	Flu Vaccine Response in Patients on Biologic Therapies	Observational study
243.	NCT03278912	Natural History of Intestinal Inflammation in People With Primary Immune Dysregulations	Observational study
244.	NCT03282903	The PRognostic Effect of Environmental Factors in Crohns and Colitis	Observational study
245.	NCT03288961	Self Screening Tool for Crohn's Perianal Fistula	Unknown status
246.	NCT03325582	MR Assessment of Perianal Crohn's Disease	Observational study
247.	NCT03369353	Precision Diagnostics in Inflammatory Bowel Disease, Cellular Therapy and Transplantation (The PREDICT Trial)	Observational study
248.	NCT03393247	The Therapy Effect of Azathioprine Initial or 14 Weeks After Combined With Infliximab on CD	Unknown status
249.	NCT03394586	Real-world Data Regarding Treatment of Ulcerative Colitis Patients With Golimumab	Observational study
250.	NCT03408249	IBDocÂ® Canadian User Performance Evaluation	Non-IBD/peripheral treatment
251.	NCT03436966	Role of Multislice CT in Diagnosis of Inflammatory Bowel Disease	Observational study
252.	NCT03445624	Abdominal Ultrasound With Doppler and Peripheral Hemogram in Assesment Inflammatory Bowel Disease	Observational study
253.	NCT03486652	Rheumatological Manifestations in Inflammatory Bowel Diseases	Unknown status
254.	NCT03492944	Contrast Enhanced Ultrasound in Human Crohns Disease-Lumason"	Observational study
255.	NCT03504930	COLISURG : Exploratory Analysis of Sexual Function and the Impact of Biotherapies on Postoperative Morbidity.	Observational study
256.	NCT03515070	Microbiome and Genetic Analysis of Familial IBD	Observational study
257.	NCT03522623	Estimating Indirect and OOP Costs in Pediatric Inflammatory Bowel Disease: a National Study	Observational study
258.	NCT03541733	Bowel Preparation for Magnetic Resonance Enterography	Non-IBD/peripheral treatment
259.	NCT03559517	Efficacy and Safety Study of Ontamalimab as Induction Therapy in Participants With Moderate to Severe Crohn's Disease (CARMEN CD 305)	Terminated
260.	NCT03565263	Functional Gastrointestinal Disorders in Pediatric IBD	Observational study
261.	NCT03565432	Sick Leave, Work Disability and Quality of Life in Korean Patients With Inflammatory Bowel Diseases	Observational study
262.	NCT03566823	Efficacy and Safety Study of Ontamalimab as Induction Therapy in Participants With Moderate to Severe Crohn's Disease (CARMEN CD 306)	Terminated
263.	NCT03571373	PIBD-SETQuality; the PIBD-NET Inception Cohort and Safety Registry	Unknown status
264.	NCT03589183	Gut Microbiome in Inflammatory Bowel Disease	Observational study
265.	NCT03593252	Bowel Preparation in Elective Pediatric Colorectal Surgery	Non-IBD/peripheral treatment
266.	NCT03627091	Efficacy and Safety Study of Ontamalimab as Maintenance Treatment in Participants With Moderate to Severe Crohns Disease (CARMEN CD 307)	Terminated
267.	NCT03630146	iPeer2Peer Program for Adolescents With Inflammatory Bowel Disease	Non-IBD/peripheral treatment
268.	NCT03640637	The Role of PET/MRI in the Diagnosis and Treatment of Children and Adolescents With Inflammatory Bowel Diseases	Observational study
269.	NCT03662646	Circadian Rhythms in Patients With IBD or Celiac Disease Upon Diagnosis and Medical Follow-up	Observational study
270.	NCT03668249	A Study to Characterize Multidimensional Model to Predict the Course of Crohns Disease (CD)	Observational study
271.	NCT03681652	Post-Operative Crohns Disease Outcome in Children	Observational study
272.	NCT03698812	A Multicenter Prospective Cohort Study on the Clinical Prognosis of Ulcerative Colitis	Unknown status
273.	NCT03706664	Small Bowel Deep Learning Algorithm Project	Non-IBD/peripheral treatment
274.	NCT03719209	Using Virtual Reality for Patients With Gastrointestinal Disease	Unknown status

No.#	NCT Number	Title (short)	Reason for exclusion
275.	NCT03747718	Randomized Trial Comparing Single vs. Maintenance Fecal Microbiota Transplant for Refractory Crohns Disease in Children"	Withdrawn
276.	NCT03751722	Genetic an Functional Studies of Patient With Inflammatory Bowel Disease	Observational study
277.	NCT03752372	Microbiome Alterations in IL10RA-deficient Patients After HSCT	Terminated
278.	NCT03769922	Postoperative Progression of the Disease Following Extensive Versus Limited Mesenteric Excision for Crohns Disease	Non-IBD/peripheral treatment
279.	NCT03774329	Impact of a Physical Activity Program on Bone Mineral Density in Children and Adolescents With Chronic Inflammatory Bowel Disease	Non-IBD/peripheral treatment
280.	NCT03813862	Observational Pharmaco-Epidemiology Research & Analysis	Observational study
281.	NCT03820284	Association Between Helicobacter Pylori and Inflammatory Bowel Disease	Observational study
282.	NCT03824561	Specified Drug-Use Survey on Vedolizumab for IV Infusion 300 mg [Ulcerative Colitis]	Observational study
283.	NCT03827109	Peer Mentoring to Improve Self-management in Youth With IBD	Non-IBD/peripheral treatment
284.	NCT03833310	Novel Biomarkers in Inflammatory Bowel Disease	Observational study
285.	NCT03841045	Unraveling a Potential Connection Between Bilirubin Metabolism, Gut Microbiota and Inflammatory Bowel Diseases	Unknown status
286.	NCT03847753	Exploring the Comorbidity Between Mental Disorders and General Medical Conditions	Observational study
287.	NCT03860012	Folic Acid in Pediatric Inflammatory Bowel Disease	Non-IBD/peripheral treatment
288.	NCT03863704	Transcutaneous VNS to Treat Pediatric IBD	Non-IBD/peripheral treatment
289.	NCT03868332	Predictors of Subclinical Atherosclerotic Cardiovascular Diseases (ASCVD) in Patients With Inflammatory Bowel Disease	Observational study
290.	NCT03884439	Infliximab Biosimilar Pfizer Drug Use Investigation (Crohns Disease or Ulcerative Colitis)	Observational study
291.	NCT03884686	Promote Food. Improving Food-related Quality of Life in Inflammatory Bowel Disease	Unknown status
292.	NCT03886532	GI Medical Cannabis Study on IBD	Withdrawn
293.	NCT03886753	Pharmacokinetic (PK) and Pharmacodynamics (PD) Study of Ilera Specific Products	Terminated
294.	NCT03909542	Malnutrition Associated With Complications After Ileostomy Reversal	Observational study
295.	NCT03913962	Health and Exercise Response in Children With Chronic and Auto-immune Pathologies	Observational study
296.	NCT03938324	Peer i-Coaching for Activated Self-Management Optimization in Adolescents and Young Adults With Chronic Conditions	Non-IBD/peripheral treatment
297.	NCT03949257	Dynamic Changes of Gut Microbiota in UC After FMT	Non-IBD/peripheral treatment
298.	NCT03950726	Ileocolic Resection for Complex Crohn's Disease	Observational study
299.	NCT03952195	Complications and Outcomes of Pouch Excision	Observational study
300.	NCT03952364	The PRECIOUS Study: Predicting Crohns & ColitIs Outcomes in the United States	Observational study
301.	NCT03953768	VNS Prospective Neuromodulation of Autonomic, Immune and Gastrointestinal Systems	Non-IBD/peripheral treatment
302.	NCT03958266	MyIBD Care - Feasibility Study	Withdrawn
303.	NCT03967236	Impact of Intestinal Virome on Pediatric Inflammatory Bowel Disease	Observational study
304.	NCT03980405	Combination of Diet and 5ASA (5-aminosalicylic Acid) for Ulcerative Colitis	Terminated
305.	NCT03983837	Elemental Diet for Treatment of Inflammatory Bowel Disease in Patients With Chronic Granulomatous Disease	Non-IBD/peripheral treatment
306.	NCT03986996	Antimicrobial Therapy for Ulcerative Colitis (UC)	Terminated
307.	NCT03998215	Diphtheria Vaccination in Adolescents With Inflammatory Bowel Disease	Observational study
308.	NCT03998449	Immunogenicity of Cholera Vaccine in Children With IBD	Observational study
309.	NCT04002180	Specified Drug-Use Survey on Vedolizumab for IV Infusion 300 mg [Crohn's Disease]	Observational study
310.	NCT04017637	Resistance Training Intervention to Promote Lean Mass in Youth With IBD	Withdrawn
311.	NCT04060303	ENhanced Recovery in CHildren Undergoing Surgery	Non-IBD/peripheral treatment
312.	NCT04067934	JUMP Inflammatory Bowel Disease Study	Non-IBD/peripheral treatment
313.	NCT04088773	Biomarkers of Intestinal Fibrosis in Small Bowel Crohns Disease"	Observational study

No.#	NCT Number	Title (short)	Reason for exclusion
314.	NCT04089501	The Role of the Pregnane X Receptor (PXR) in Indole Signaling and Intestinal Permeability in Inflammatory Bowel Disease	Observational study
315.	NCT04100239	A Pilot and Feasibility Trial of a Group Mindfulness and Self-compassion Intervention for Young People Diagnosed With IBD	Non-IBD/peripheral treatment
316.	NCT04105348	Diabetes Mellitus and Inflammatory Bowel Disease	Observational study
317.	NCT04119635	Pediatric Penetrating Crohns Disease"	Observational study
318.	NCT04131504	Precision Crohns Disease Management Utilizing Predictive Protein Panels (ENvISION)	Observational study
319.	NCT04132024	Feasibility of Insomnia Tracking and Treatment in IBD	Non-IBD/peripheral treatment
320.	NCT04135027	Study of Factors and Mechanisms Influencing the Effects of Treatments in Crohn's Disease	Observational study
321.	NCT04148274	A Non-Interventional Pilot Study to Explore the Role of Gut Flora in Ulcerative Colitis	Observational study
322.	NCT04170192	Multi-center Clinical Study of Cord Blood Stem Cell Transplantation for IBD Caused by IL-10R Gene Deficiency	Observational study
323.	NCT04178408	IBD Registry in a Sub-Saharan African Population	Observational study
324.	NCT04179201	Study on Clostridium Difficile Infection in Chinese Patients With Inflammatory Bowel Disease	Unknown status
325.	NCT04187820	Effect of Acupuncture on Mild to Moderate Active Crohns Disease"	Non-IBD/peripheral treatment
326.	NCT04198831	Effect of Acupuncture on Quiescent Crohns Disease	Non-IBD/peripheral treatment
327.	NCT04205734	Methotrexate Polyglutamate Measurements in Patients With Inflammatory Bowel Disease	Suspended
328.	NCT04207008	Trial of a Decision Support Intervention for Adolescents and Young Adults With Ulcerative Colitis	Non-IBD/peripheral treatment
329.	NCT04211129	Effect of Acupuncture on Patients With Crohns Disease in Remission	Non-IBD/peripheral treatment
330.	NCT04256538	Identify Specific Peripheral Blood Mononuclear Cell (PBMC) MiRNA as Biomarkers for Inflammatory Bowel Disease (IBD)	Observational study
331.	NCT04269720	Biofeedback in Pediatric Inflammatory Bowel Disease	Withdrawn
332.	NCT04283864	Decoding Personalized Nutritional, Microbiome and Host Patterns Impacting Clinical and Prognostic Features in Crohns Disease"	Observational study
333.	NCT04286659	Receptor for Advanced Glycation End Products (RAGE) Polymorphisms In Inflammatory Bowel Disease	Observational study
334.	NCT04290156	The Effect of Joint Transition Visits on Quality of Life in Inflammatory Bowel Diseases (TRANS-IBD)	Non-IBD/peripheral treatment
335.	NCT04296500	A Development of Inflammatory Bowel Disease Pattern Identification Algorithm Using Case Series Data	Observational study
336.	NCT04297566	MICISPORT Survey : Questionnaire	Observational study
337.	NCT04306939	Genomic Resources for Enhancing Available Therapies (GREAT1.0) Study	Observational study
338.	NCT04308850	Exploring the Effects of Vitamin D Supplementation on the Chronic Course of Patients With Crohns Disease With Vitamin D Deficiency"	Non-IBD/peripheral treatment
339.	NCT04309058	Observation of the Effect of Vitamin D Supplementation on Chronic Course of Patients With Ulcerative Colitis Based on Vitamin D Receptor Fok I Gene Polymorphism	Non-IBD/peripheral treatment
340.	NCT04312659	A Study of Infliximab in the Treatment of Chinese Children With Crohns Disease	Observational study
341.	NCT04324632	Magnetic Resonance Enterography in Inflammatory Bowel Diseases	Unknown status
342.	NCT04328259	Prevalance of non_ Alcoholic Fatty Liver Diseases in Patient With Inflammatory Bowel Diseases Attending Assiut University Hospitals	Unknown status
343.	NCT04332328	Study of Demographic and Dietary Profile in Patients With Ulcerative Colitis in Upper Egypt	Observational study
344.	NCT04348890	Proof of Concept Trial of Vamorolone in Pediatric Ulcerative Colitis	Withdrawn
345.	NCT04349943	Effect of Standardized Nutritional Therapy on Clinical Prognosis and Cost-effectiveness of Inflammatory Bowel Disease	Unknown status
346.	NCT04408703	PRognOstiC valuE of rEd Density in Ulcerative Colitis	Unknown status
347.	NCT04410484	PREPARE-IBD: Physician Responses to Disease Flares and Patient Adaptation in Relation to Events in Inflammatory Bowel Disease During COVID-19 Pandemic	Observational study

No.#	NCT Number	Title (short)	Reason for exclusion
348.	NCT04411784	PROTECT-ASUC: Covid-19 Pandemic Response Of assessmenT, EndosCopy and Treatment in Acute Severe Ulcerative Colitis	Observational study
349.	NCT04457518	Nutritional Status in Children With IBD	Observational study
350.	NCT04488198	Acupuncture in Pediatrics and Adolescents With Chronic Inflammatory Bowel Diseases	Non-IBD/peripheral treatment
351.	NCT04488471	Impact of Isolation in Patients With IBD During the COVID-19 Crisis	Unknown status
352.	NCT04499495	Assessment of 5-Aminosalicylic Acid Prescription Patterns and Treatment Outcomes in Patients With Ulcerative Colitis in Korea	Observational study
353.	NCT04508088	Skeletal Health and Bone Marrow Composition in Newly Diagnosed Adolescents With Crohn Disease	Non-IBD/peripheral treatment
354.	NCT04528355	Data Collection Study of Patients With Non-Malignant Disorders Undergoing UCBT, BMT or PBSCT With RIC	Observational study
355.	NCT04530877	Exclusive Enteral Nutrition vs. Infliximab in Chinese CD Patients	Withdrawn
356.	NCT04535882	Effectiveness of Serum Leucine-rich Alpha-2 Glycoprotein Levels on IBD	Observational study
357.	NCT04538638	Mesenteric SParIng Versus Central mesenterectomY in Ileocolic Resection for Terminal Ileitis in Crohns Disease	Non-IBD/peripheral treatment
358.	NCT04564638	Long-term Outcome of Patients With Acute Ulcerative Colitis After First Course of Intravenous Corticosteroids	Observational study
359.	NCT04601805	Optical Coherence Tomography Angiography Assessment of Retinal Lesions in Inflammatory Bowel Diseases	Observational study
360.	NCT04631965	Healthcare Transition of Adolescents With Chronic Health Conditions	Observational study
361.	NCT04643483	A Study to Test Efficacy, Safety, and Pharmacokinetics of Certolizumab Pegol in Children and Adolescents With Moderately to Severely Active Crohns Disease	Withdrawn
362.	NCT04647578	Physical Fitness Levels in Children and Adolescents With Chronic Inflammatory Bowel Disease (CIBD): Crohns Disease, Ulcerative Colitis, Chronic Unclassified Colitis	Observational study
363.	NCT04650867	Non Invasive Characterization of Pediatric Inflammatory Bowel Diseases Using Multispectral Optoacoustic Tomography	Observational study
364.	NCT04655729	Biomarkers to Predict and Monitor Response to Infliximab	Observational study
365.	NCT04660136	Usefulness of Contrast-enhanced Ultrasound (CEUS) in Crohns Disease Pediatric Patients"	Non-IBD/peripheral treatment
366.	NCT04760964	Mitochondrial DAMPs as Mechanistic Biomarkers of Mucosal Inflammation in Crohns Disease and Ulcerative Colitis	Observational study
367.	NCT04803916	Pre-stenotic Inflammation Following Endoscopic Balloon Dilatation in Crohn's Disease: A Prospective Study	Observational study
368.	NCT04838834	Monitoring Serologic Response to Severe Acute Respiratory Syndrome-2 (SARS-COV-2)/COVID-19 in Children	Observational study
369.	NCT04867408	Endoscopic Severity Image Recognition to Advance Research and Training in Inflammatory Bowel Disease (EVEREST - IBD)	Observational study
370.	NCT04881370	Quality of Life for Patients With Chronic Paediatric Onset Inflammatory Bowel Disease (IBD)	Terminated
371.	NCT04893928	Role of Information and Communication Technologies for Health Support in Inflammatory Bowel Diseases: The DAMASCO Trial	Observational study
372.	NCT04923100	Therapeutic Effect of New Biologics in Crohns Disease	Unknown status
373.	NCT04933214	Adherence to Exclusive Enteral Nutrition in Patients With Crohns Disease	Observational study
374.	NCT04948723	Vaccination Practices for Inflammatory Bowel Disease Patients in China With Gastroenterologists	Observational study
375.	NCT04956991	CT Diagnoses in Crohns Disease Activity	Observational study
376.	NCT04963725	A Study of Symptom Improvement of Ulcerative Colitis After an Induction Dose of Ustekinumab in Japanese Clinical Practice	Observational study
377.	NCT04965584	Microflora and Metabolomics of Crohns Disease Patients	Observational study
378.	NCT04976491	Based on the Special Disease Management of Crohns Disease Diet Studies	Observational study
379.	NCT04989374	Regulation of Vitamin D Metabolism by Washed Microbiota Transplantation in Patients With IBD	Observational study
380.	NCT04990245	pAtient rePorted Outcomes Linked With histoLogy in Patients With uLcerative cOlitis	Observational study
381.	NCT05002166	Clinical Characteristics and Risk Factors of Children Suffering From Inflammatory Bowel Diseases At Assiut University Children Hospital	Observational study

No.#	NCT Number	Title (short)	Reason for exclusion
382.	NCT05014243	Targeted Metabolomics to Decipher Dietary Contributions to Pediatric Inflammatory Bowel Disease (IBD) - Main Study	Observational study
383.	NCT05071404	Efficacy Assessment and Monitoring of Subcutaneous Infliximab Levels.	Observational study
384.	NCT05112497	Mother-to-Infant Transfer of Bacteriome, Fungome, Virome, and Metabolome in Health and Crohns Disease"	Observational study
385.	NCT05117996	Simplified Bowel Preparation Versus Standard Bowel Preparation Before Capsule Endoscopy in Patients With Crohns Disease"	Non-IBD/peripheral treatment
386.	NCT05135221	Combination Therapy With Two Biologicals	Observational study
387.	NCT05151458	Serum NGAL as a Predictor of Clinical and Endoscopic Activity of Inflammatory Bowel Disease	Observational study
388.	NCT05161442	Minimally Invasive Surgery in Crohn's Disease: Laparoscopic vs Robotic	Observational study
389.	NCT05176795	Host-microbiota-environment Interactions	Observational study
390.	NCT05183256	Cohort of Inflammatory Bowel Disease Patient in Yeouido St. Marys Hospital"	Observational study
391.	NCT05202418	Stress in Inflammatory Bowel Disease	Non-IBD/peripheral treatment
392.	NCT05219656	Efficacy of Cyclosporine Rescue Therapy in Acute Ulcerative Colitis.	Observational study
393.	NCT05221281	Implementing a Multimodal RCT Intervention to Improve the Transition of Patients With Crohns Disease From Pediatric to Adult Care"	Non-IBD/peripheral treatment
394.	NCT05224193	ACTIVE-IBD Study: Physical Activity and Sleep in Paediatric Inflammatory Bowel Disease	Observational study
395.	NCT05242458	A Study of Ustekinumab Treatment in Children With Crohns Disease	Observational study
396.	NCT05251415	Clinico-biological Collection to Investigate the Physiopathology of Systemic Autoimmune Diseases	Observational study
397.	NCT05321485	The Effect of MedicijnWijs on Adherence to Azathioprine Therapy in Adolescents With IBD	Non-IBD/peripheral treatment
398.	NCT05330416	Comparison of Quality of Life With or Without Automatic Seton Placement in Perianal Crohn's Fistula	Non-IBD/peripheral treatment
399.	NCT05332639	Personalized Risk Estimation for Crohns Disease (PRE-CD): Implementation and Feasibility	Non-IBD/peripheral treatment
400.	NCT05386290	A Multicentered Prospective Cohort Study of Chinese IBD Patients	Observational study
401.	NCT05394272	Efficacy of Energy Therapy in the Management of Chronic Pain Medical Condition in Children and Adolescents.	Non-IBD/peripheral treatment
402.	NCT05405465	Effectiveness of Golimumab in Patients With Ulcerative Colitis: Results of a Real-life Study in Switzerland	Observational study
403.	NCT05430412	CT to Assess the Efficacy and Safety of Adding GMA to Infliximab in Paediatric Patients With Steroid-refractory Ulcerative Colitis	Non-IBD/peripheral treatment
404.	NCT05439772	Examining the Effect of Ondansetron on Bowel Prep Success	Non-IBD/peripheral treatment
405.	NCT05447572	FIT Combining FC for Predicting MH and Histology Remission in UC	Observational study
406.	NCT05452187	Evaluation of the Safety and Effectiveness of Direct-acting Antiviral Drugs in the Treatment of Hepatitis C in Patients With Inflammatory Bowel Disease: National Multicenter Study	Observational study
407.	NCT05461014	RxWell in Pediatric IBD for Disease Management and Transition Readiness	Non-IBD/peripheral treatment
408.	NCT05500040	Changes in Faecal Calprotectin Levels and Participation-related Scores Following Three Weeks of Inpatient Rehabilitation	Observational study
409.	NCT05502965	Specific Dietary Pattern for Crohns Disease Patients Following Surgery"	Non-IBD/peripheral treatment
410.	NCT05530122	Prognosis in UC After First Biological	Observational study
411.	NCT05538923	Oral Health in Children With Inflammatory Bowel Disease	Observational study
412.	NCT05542368	Study of Thyroid Status in Patients With IBD	Observational study
413.	NCT05570617	Transition to Adulthood Through Coaching and Empowerment	Non-IBD/peripheral treatment
414.	NCT05572008	ProteIn Nutrition in Crohns Disease"	Non-IBD/peripheral treatment
415.	NCT05598346	Significance of MAIT Cells in Inflammatory Bowel Disease	Observational study
416.	NCT05615116	Clinical Study on the Distribution of Digestive Tract Microbiota Before and After Ileocecal Resection in Crohns Disease"	Observational study

No.#	NCT Number	Title (short)	Reason for exclusion
417.	NCT05636657	Comparative Study of Intestinal Color Ultrasound and Capsule Endoscopy in Monitoring Crohns Disease"	Observational study
418.	NCT05645055	Biologics for Stricturing Crohn's Diseases	Observational study
419.	NCT05645666	The Effectiveness of Well-being Therapy on Mental Health and Self-efficacy of Patients With Ulcerative Colitis	Non-IBD/peripheral treatment
420.	NCT05649462	Effect of Vitamin D Supplementation on Vedolizumab Response in Patients With Ulcerative Colitis	Observational study
421.	NCT05652491	The Role of Gut Microbiome in Predicting Comorbidities and Complications in Children With Inflammatory Bowel Disease	Observational study
422.	NCT05658081	Comparison of Two Stapled Antimesenteric Anastomosis for CD: a Randomized Control Trial Study	Non-IBD/peripheral treatment
423.	NCT05663671	Evaluate Established Anti-DEFA5 mAbs Diagnostic Efficacy and Safety in IBD	Observational study
424.	NCT05673278	Non-Invasive Monitoring Through Bowel Ultrasound in Paediatric Inflammatory Bowel Disease Study	Non-IBD/peripheral treatment
425.	NCT05689892	Shared Decision Making in Paediatric Inflammatory Bowel Disease	Non-IBD/peripheral treatment
426.	NCT05705232	Inflammatory Marker in Ibd and Peripheral Arthritis	Observational study
427.	NCT05709717	Regenerative Therapy With Autologous Stromal Vascular Fraction Derived Mesenchymal Stem Cells and Platelet-rich Plasma to Treat Complex Perianal Diseases	Observational study
428.	NCT05710276	Assessment of Intestinal Inflammation by Infrared Thermography in Pediatric Crohn Disease	Non-IBD/peripheral treatment
429.	NCT05713409	Prediction and Close Monitoring of Postoperative Recurrence by Intestinal Ultrasound After Ileocecal Resection in Crohns Disease Patients	Observational study
430.	NCT05777616	The Impact of Medication Belief on Adherence to Infliximab in Patients With Crohns Disease"	Observational study
431.	NCT05781152	Clinical, Imaging, and Endoscopic Outcomes of Children Newly Diagnosed With Crohns Disease	Observational study
432.	NCT05791526	An Observational Study to Assess Change in Disease Activity and Adverse Events of Rinvoq in Adult Participants With Moderate to Severe Ulcerative Colitis (UC) in Japan	Observational study
433.	NCT05796245	A Study to Learn About the Study Medicine Called Infliximab (Genetical Recombination) [Infliximab Biosimilar 3] in People with Rheumatoid Arthritis, Ulcerative Colitis, Crohns Disease, or Psoriasis	Observational study
434.	NCT00035074	A Pharmacokinetic Study to Determine the Oral Bioavailability of Methotrexate in Patients With Inflammatory Bowel Disease	Insufficient data
435.	NCT00040521	Immunology of Inflammatory Bowel Disease: Molecular Mechanisms and Therapeutics	Insufficient data
436.	NCT00048113	Immunology of Inflammatory Bowel Disease: Molecular Mechanisms and Therapeutics	Insufficient data
437.	NCT00048295	Alicaforsen (ISIS 2302) in Patients With Active Crohn's Disease	Insufficient data
438.	NCT00055367	Safety, Tolerability and Effectiveness of Natalizumab in Adolescents With Active Crohn's Disease	Insufficient data
439.	NCT00073047	Humanized Anti-IL-2 Receptor Monoclonal Antibody in Moderate-to-severe Ulcerative Colitis	Insufficient data
440.	NCT00074542	An Efficacy and Safety Study of Omega-3 Free Fatty Acids (Epanova™) for the Maintenance of Symptomatic Remission in Subjects With Crohn's Disease	Insufficient data
441.	NCT00219414	Study for the Treatment of Ulcerative Colitis With Adacolumn (Companion to US Study 512-04-205)	Insufficient data
442.	NCT00254618	A Study of Asacol Absorption, Metabolism and Excretion in Children and Adolescents With Ulcerative Colitis.	Insufficient data
443.	NCT00325078	Infliximab to Treat Crohn'S-like Inflammatory Bowel Disease in Chronic Granulomatous Disease	Insufficient data
444.	NCT00446433	A Study to Evaluate CC-5013 in the Treatment of Adolescents and Adults With Moderately Severe Crohn's Disease	Insufficient data
445.	NCT00534131	Anterior Perineal Plane for Ultra Low Anterior Resection of the Rectum	Non-IBD/peripheral treatment
446.	NCT00534911	Reducing Depressive Symptoms in Physically Ill Youth	Non-IBD/peripheral treatment

No.#	NCT Number	Title (short)	Reason for exclusion
447.	NCT00583232	Protein and Energy Metabolism in Pediatric Crohn's Disease	Non-IBD/peripheral treatment
448.	NCT00586352	Protein Metabolism in Newly Diagnosed Pediatric Inflammatory Bowel Disease	Non-IBD/peripheral treatment
449.	NCT00679003	Managing Inflammatory Bowel Disease	Non-IBD/peripheral treatment
450.	NCT00724529	Post Marketing Surveillance of Remicade	Observational study
451.	NCT00724958	Program Extension of Real Life Dosing of Remicade in Austria for Crohn's Disease (Study P04052)(COMPLETED)	Observational study
452.	NCT00781638	ADAPT Adacolumn Pediatric Trial in Ulcerative Colitis	Non-IBD/peripheral treatment
453.	NCT00922103	A Study to Investigate the Ileo Neo Rectal Anastomosis Compared to the Ileo Pouch Anal Anastomosis for Patients With Ulcerative Colitis	Non-IBD/peripheral treatment
454.	NCT01111708	Clinical Outcome After Close Rectal Ileo Pouch Anal Anastomosis for Colitis Ulcerosa(CU)	Non-IBD/peripheral treatment
455.	NCT01173250	Is Diverting Ileostomy Necessary in Stapled Ileoanal Pouch?	Non-IBD/peripheral treatment
456.	NCT01237847	A Brief Intervention to Improve Adherence in Teens With Inflammatory Bowel Disease	Non-IBD/peripheral treatment
457.	NCT01345318	B0151005 Open-Label Extension Study	Insufficient data
458.	NCT01764542	Development of Dysplasia in the Pelvic Pouch in Patients With Ulcerative Colitis (UC) and Risk Factors	Non-IBD/peripheral treatment
459.	NCT01860651	Web-based Monitoring in Children and Adolescents With Inflammatory Bowel Disease	Non-IBD/peripheral treatment
460.	NCT01942720	Monitoring Disease Activity Using Video Capsule Endoscopy (VCE) in Crohn's Disease (CD) Subjects	Non-IBD/peripheral treatment
461.	NCT02264275	Influence of Aerobic Exercise Training (AET) on Inflammatory Bowel Disease (IBD) in Children and Adolescents	Non-IBD/peripheral treatment
462.	NCT02265588	Healthy Approach to Physical and Psychological Problems in Youngsters With IBD (HAPPY-IBD).	Non-IBD/peripheral treatment
463.	NCT02488005	The Use of Small Bowel Ultrasound to Predict Response to Remicade Induction	Non-IBD/peripheral treatment
464.	NCT02559037	Acupuncture Treatment for Active Crohn's Disease	Non-IBD/peripheral treatment
465.	NCT02893670	Tailored Transition for IBD Adolescents	Non-IBD/peripheral treatment
466.	NCT03161886	A Treat-to-target Strategy Using Pan-enteric Capsule Endoscopy (PCE) in Paediatric Crohn's Disease (CD).	Non-IBD/peripheral treatment
467.	NCT03189888	Granulocyte-monocyte Apheresis in Ulcerative Colitis	Non-IBD/peripheral treatment
468.	NCT03338894	A Trial of Yoga in Pediatric Inflammatory Bowel Disease	Non-IBD/peripheral treatment
469.	NCT03340519	Early MRI Prediction of Crohns	Non-IBD/peripheral treatment
470.	NCT03576027	Hyperbaric Oxygen Therapy in Children With Inflammatory Bowel Diseases	Non-IBD/peripheral treatment
471.	NCT03643211	Xeljanz Special Investigation for Long-term Use in UC Patients	Observational study
472.	NCT03662919	One-year Persistence to Treatment of Participants Receiving Flixabi or Imraldi: a French Cohort Study	Observational study
473.	NCT03670693	Non-invasive Approaches to Identify the Cause of Fatigue in Inflammatory Bowel Disease Patients.	Non-IBD/peripheral treatment
474.	NCT01536509	Improving Medication Adherence in Pediatric Inflammatory Bowel Disease	Non-IBD/peripheral treatment
475.	NCT02085083	Improving OutcoMes in the Pediatric to Adult Care Transition in Inflammatory Bowel Disease	Non-IBD/peripheral treatment
476.	NCT03809195	Clinical Hypnosis in Pediatric Crohn's Disease	Non-IBD/peripheral treatment
477.	NCT04757935	Yoga for Youth With IBD: a Pilot Feasibility Study	Non-IBD/peripheral treatment

No.#	NCT Number	Title (short)	Reason for exclusion
478.	NCT05591976	Exercise Training in Youth With Inflammatory Bowel Disease	Non-IBD/peripheral treatment
479.	NCT02624037	Precision IFX: Using a Dashboard to Individualize Infliximab Dosage	Non-IBD/peripheral treatment
480.	NCT05817266	Role of FCI in the Detection of Proctosigmoiditis Caused by IBD	Withdrawn
481.	NCT05838196	Service Development of POCUS in the IBD Clinic	Non-IBD/peripheral treatment
482.	NCT05841537	An Observational Study to Assess Change in Disease Activity and Adverse Events In Adult Participants With Moderate to Severe Active Crohn's Disease (CD)	Observational study
483.	NCT05842564	Autophagy in Paediatric Crohn's Disease	Non-IBD/peripheral treatment
484.	NCT05867784	Effect of Vitamin D Supplementation on the Efficacy of Ustekinumab in the Treatment of Crohn's Disease	Observational study
485.	NCT05878639	Efficacy and Safety of Lactulose for Bowel Preparation in Patients With Inflammatory Bowel Disease	Non-IBD/peripheral treatment
486.	NCT05916274	Exploration of the Activity of DNA Located Outside of Cellular Nucleus to Amplify Inflammation in Inflammatory Bowel Disease in Children Through Biological Pathway Cyclic GMP-AMP Synthase (cGAS) - Stimulator of Interferon Genes (STING)	Non-IBD/peripheral treatment
487.	NCT05918562	Efficacy of a Low Fiber Diet for Pediatric Colonoscopy Prep	Non-IBD/peripheral treatment
488.	NCT05953402	A Study of Ozanimod in Pregnant Women With Ulcerative Colitis and Their Offspring	No pediatric patients
489.	NCT05955963	Bowel Sounds Analysis in Paediatric Inflammatory Bowel Disease: Relationship With wPCDAI	Non-IBD/peripheral treatment
490.	NCT05974332	Oncostatin M in Ulcerative Colitis Patients	Diagnostic study
491.	NCT06013696	Bleach and Urine Color in UC Patients Using 5ASA	Non-IBD/peripheral treatment
492.	NCT06023030	An Observational Study to Assess Change in Disease Activity and Adverse Events in Adolescent and Adult Participants With Moderate to Severe Active Crohn's Disease (CD) in Japan	Observational study
493.	NCT06023667	SMART-IBD App Trial	Non-IBD/peripheral treatment
494.	NCT06042387	Diverse Ancestry Study in IBD	Observational study
495.	NCT06065228	Therapeutic Endpoint in Pediatric IBD Conditions	Observational study
496.	NCT06065995	StoMakker Mobile Application	Non-IBD/peripheral treatment
497.	NCT06096987	Teen-to-Teen Videos to Improve Electronic Medical Record Portal Knowledge and Personal Health Information Engagement	Non-IBD/peripheral treatment
498.	NCT06107179	Monitoring of Pediatric Inflammatory Bowel Diseases Using Multispectral Optoacoustic Tomography	Observational study
499.	NCT06109961	Hyperbaric Oxygen Therapy for Perianal Fistulizing Crohn's Disease Not Responsive or Intolerant to Conventional Care: HYPNOTIC Pilot Study	Non-IBD/peripheral treatment
500.	NCT06120322	Machine Learning Approach to Study the Interactions Between Environment and Intestinal Tissue Homeostasis in IBD	Observational study
501.	NCT06124287	MRE Predictors of Disease Relapse After Stopping Biologics	Observational study
502.	NCT06124833	Integrated Gut Microbiome Data in Inflammatory Bowel Disease	Observational study
503.	NCT06126835	A Study to Evaluate the Safety of Ozanimod Exposure During Pregnancy in Women With Ulcerative Colitis and Their Infants	Observational study
504.	NCT06152289	Development of New Diagnostic Tools in Capsule Endoscopy	Observational study
505.	NCT06162936	Neutrophil Oxidative Burst in Early and Late Onset Pediatric Inflammatory Bowel Disease	Observational study
506.	NCT06186752	Feasibility Test of Action Planning in Pediatric IBD	Non-IBD/peripheral treatment
507.	NCT06228352	Role of Bile Acids and Microbiota in Clostridioides Difficile Infection in Ulcerative Colitis	Observational study
508.	NCT06241170	Comparing Surgical Approaches for Crohn's Disease Recurrence	Non-IBD/peripheral treatment
509.	NCT06249243	Affection of Renal and Urinary System in IBD Patients	Observational study

No.#	NCT Number	Title (short)	Reason for exclusion
510.	NCT06270277	Evaluation of Quality of Life and Psychosocial Status in Children With Inflammatory Bowel Disease	Diagnostic study
511.	NCT06315179	Seattle Spatial Transcriptomic Research in Inflammatory Bowel Disease Evaluation (STRIDE)	Observational study
512.	NCT06324591	Observational Prospective Study to Identify Intestinal Microbial Profiles in Newly Diagnosed Crohn's Disease Patients	Observational study
513.	NCT06362174	Capsule & Omics for pRedicting Exacerbation of Crohn's Disease	Observational study
514.	NCT06364722	Effect of Acupuncture on the Microbiota-gut-brain Axis in Crohn's Disease	Non-IBD/peripheral treatment
515.	NCT06371820	ESPRESSO (Epidemiology Strengthened by histoPathology Reports in Sweden)	Observational study
516.	NCT06382519	Thalidomide Therapy for VEOIBD	Observational study
517.	NCT06388057	Intraoperative Ultrasound in Crohn's Disease	Non-IBD/peripheral treatment
518.	NCT03478956	A Phase I Study of Etrolizumab Followed by Open-Label Extension and Safety Monitoring in Pediatric Patients With Moderate to Severe Ulcerative Colitis or Moderate to Severe Crohn's Disease (FENNEL)	Terminated
519.	NCT06954311	Therapeutic Drug Monitoring for Biological Therapy in Pediatric Inflammatory Bowel Disease	Observational study
520.	NCT06946810	Impact of Transition Programs on Readiness and Quality of Life in IBD	Non-IBD/peripheral treatment
521.	NCT06922331	Upadacitinib for Refractory IBD in Asian Children and Elderly: Age-Stratified Analysis	Observational study
522.	NCT06856135	Expanded Access to Vedolizumab for Children and Teenagers With Ulcerative Colitis or Crohn's Disease in the USA	Observational study
523.	NCT06723418	Development and Validation of a New Paediatric Inflammatory Bowel Disease NUTrition Risk Score (PIBD-NUTS)	Observational study
524.	NCT06673134	Prospective Cohort Establishment and Clinical Observation of Children With Crohn's Disease	Observational study
525.	NCT06606756	CEDATA GPGE - a Patient Registry for Children and Adolescents with Inflammatory Bowel Disease	Observational study
526.	NCT02522169	TOPIT; Trough Level Optimized Pediatric Inflammatory Bowel Disease Therapy: A Multicenter Study Comparing Effectiveness of Trough Level Optimized Infliximab Maintenance Therapy With Standard Dosing Regimen of Pediatric Patients With Crohn's Disease (TOPIT)	Unknown status
527.	NCT0427 6636	Exploring the Effects of Caltrate Supplement on the Chronic Course of Crohn's Disease Patients With Vitamin D Deficiency	Unknown status
528.	NCT00510978	Probiotics in GastroIntestinal Disorders (PROGID)	Unknown status
529.	NCT03582969	Capsulized Fecal Microbiota Transplantation in Pediatric Ulcerative Colitis Patients (FMT UC)	Unknown status
530.	NCT04186247	Personalized AZithromycin/metronidAZole Therapy in Pediatric Crohn's Disease (CD) (PAZAZ	Terminated
531.	NCT04974099	Personalized Infliximab Induction Strategy With Model-informed Dosing in Patients With Crohn's Disease (REMODEL)	Terminated
532.	NCT05280405	Early Proactive Therapeutic Drug Monitoring of Infliximab in Children: EPIC Study	Unknown status
533.	NCT05759104	Treatment and Fate in Fistulizing Crohn's Disease in a Pediatric Population	Unknown status
534.	NCT06033469	Therapeutic Monitoring of Infliximab and Adalimumab	Unknown status
535.	NCT05316220	A Study to Assess Adverse Events and Change in Disease Condition of Mesalamine Capsules in Children Aged 5 to 17 Years With Ulcerative Colitis	Withdrawn
536.	NCT05470985	A Study to Evaluate the Efficacy, Safety, and Drug Levels of Oral Ozanimod in Pediatric Participants With Moderately to Severely Active Crohn's Disease With an Inadequate Response to Conventional Therapy	Terminated
537.	NCT03162432	High Dose Interval Vitamin D Supplementation in Patients With IBD Receiving Remicade	Unknown status
538.	NCT03999580	The Vitamin D in Pediatric Crohn's Disease (ViDiPeC-2)	Unknown status
539.	NCT04223518	Serum Bovine Immunoglobulin (SBI) in Children and Young Adults With Inflammatory Bowel Disease (IBD)	Terminated
540.	NCT04225689	The Intensive Post Exclusive Enteral Nutrition Study (iPENS)	Unknown status
541.	NCT05539625	Mini-MARVEL - Mitochondrial Antioxidant Therapy in Ulcerative Colitis (Mini-MARVEL)	Suspended

No.#	NCT Number	Title (short)	Reason for exclusion
542.	NCT03399188	Fecal Microbiota Transplantation and Analysis of Fecal Microbiome in IBD Patients	Unknown status
543.	NCT03000296	Autologous Unselected Hematopoietic Stem Cell Transplantation for Refractory Crohn's Disease (AutoChron)	Unknown status
544.	NCT04791878	Study of Mesenchymal Stem Cells for Pediatric Perianal Fistulizing Crohn's Disease	Unknown status
545.	NCT05239702	Clinical Study of Targeting CD7 CAR-T Cells in the Treatment of Autoimmune Diseases	Unknown status
546.	NCT04701411	A Study of Darvadstrocel for Treating Complex Perianal Fistulas in Children and Teenagers With Crohn's Disease	Terminated
547.	NCT05662059	Respiratory-gated Auricular Vagal Afferent Nerve Stimulation (RAVANS) Study in Ulcerative Colitis	Unknown status
548.	NCT06443502	A Study to Learn About the Safety of Vedolizumab and How Well it Works in Children and Teenagers With Active Chronic Pouchitis	Non-IBD patients
549.	NCT04331639	High Dose Interval Vitamin D Supplementation in Patients With Inflammatory Bowel Disease Receiving Biologic Therapy	Observational study
550.	NCT03950336	Prebiotics and Diet to Reduce "Leaky" Gut in First Degree Relatives of Crohn's Disease Patients	No IBD patients
551.	NCT05284136	Exclusion Diet vs corticosteroIds in patientS With activE Crohn's Disease	Unknown status
552.	NCT06353633	The Effect of Exclusion Diet for Crohn's Disease Plus Enteral Nutrition on Children and Adolescents With Crohn's Disease	Unknown status
553.	NCT06324513	Evaluation of the Efficacy and Tolerability of the Mediterranean Diet Pattern in the Exclusion Diet for Patients With Crohn's Disease.	Unknown status
554.	NCT06116643	Pilot Study of a New Medical Food in the Management of Ulcerative Colitis	Unknown status
555.	NCT01790061	Standardized Fecal Microbiota Transplantation for Ulcerative Colitis	Unknown status
556.	NCT07240649	Outcomes From Hyperbaric Oxygen (HBO2) Treatment for Emerging Indications	Peripheral treatment
557.	NCT07409376	Comparison of the Therapeutic Effects of Balloon Assisted Enteroscopy Assisted Narrow Incision and Balloon Dilation in the Treatment of Crohn's Disease Small Intestinal Stenosis (CD stenosis)	Non-IBD specific treatment
558.	NCT07243639	Efficacy and Safety of Etrasimod in Elderly Patients With Ulcerative Colitis	No pediatric patients
559.	NCT07505030	To Evaluate the Safety, Tolerability, Pharmacokinetics, Pharmacodynamics, and Immunogenicity of LYNC-101 for Injection in Healthy Adult Participants	No pediatric patients
560.	NCT07612267	A Study to Measure Mirikizumab Levels in Breast Milk of Lactating Participants Receiving Mirikizumab Therapeutically for an Approved Indication	No pediatric patients
561.	NCT07136116	Study to Assess Change in Disease Activity of Risankizumab Treatment in Japanese Participants With Moderate to Severe Ulcerative Colitis	Observational study
562.	NCT07198113	COMPARE - Pediatric Inflammatory Bowel Disease (PIBD)	Observational study
563.	NCT07211789	Real World Evidence Study With Restrictive Diet and Otral Nutritional Supplementation on Pediatric Patients With Active Chron Disease	Observational study
564.	NCT07231601	Laparoscopic Right Hemicolectomy for Crohn's Disease of the Terminal Ileum Utilizing the Mesenteric Excision and Exclusion	Observational study
565.	NCT07240168	CurQD as Add-On Therapy in Vedolizumab-Treated Ulcerative Colitis Patients: Retrospective Cohort Study	Observational study
566.	NCT07261826	Real-World Study on Fecal Microbiota Transplantation: Long-Term Effectiveness and Safety Statistics	Observational study
567.	NCT07273760	VedAryoÂ® (Vedolizumab) Effectiveness and Safety Evaluation	Observational study
568.	NCT07385131	Prophylactic Dexamethasone Before Infliximab in Moderate-to-Severe IBD	Observational study
569.	NCT07459686	An Observational Study to Learn About Velsipity After Long Term Use in Patients With Ulcerative Colitis	Observational study
570.	NCT07472309	Effectiveness and Safety of Upadacitinib for Acute Severe Ulcerative Colitis	Observational study
571.	NCT07487311	A Prospective Cohort Study Evaluating the Efficacy and Safety of Guselkumab (GUS) With JAK Inhibitors in Patients With Difficult-To-Treat Inflammatory Bowel Disease (IBD)	Observational study
572.	NCT07660289	Combined Advanced Targeted Therapy for Inflammatory Bowel Disease	Observational study

Supplementary Table 1b: Excluded Studies – ClinicalTrialsRegister.eu (n=67)

No.#	EudraCT Number	Title	Reason for exclusion
1.	2004-001164-41	An exploratory, open, two-arm, match-control, randomised and stratified pilot phase ii study assessing the prevention of the adverse effects of long-term high-dose glucocorticoid therapy by growth	Prematurely ended
2.	2005-005414-20	Randomized controlled double-blind Vs. placebo multicentre study on the safety and effectiveness of thalidomide in the treatment of refractory Crohn s disease and ulcerative colitis	Duplicate study
3.	2008-001252-52	A multicenter, randomized, double-blind, placebo-controlled efficacy and safety trial of intravenous zoledronic acid twice yearly compared to placebo in osteoporotic children treated with glucocort	Prematurely ended
4.	2008-006484-36	Multicenter comparative study between nutritional therapy alone and anti-tnf-alpha monoclonal antibody in inducing and maintaining remission in pediatric Crohn’s disease: a randomized controlled trial	Prematurely ended
5.	2009-015864-32	A Prospective, Randomized, Double-Blind, Multicenter Study to Establish the Safety and Tolerability of Doripenem Compared With Meropenem in Hospitalized Children With Complicated Intra-Abdominal In…	Prematurely ended
6.	2010-020399-41	A 1-year, multicenter, open-label extension to CZOL446H2337 to evaluate safety and efficacy of zoledronic acid twice yearly in osteoporotic children treated with glucocorticoids	Non-IBD/peripheral treatment
7.	2010-022017-26	A pilot study on the effects of an alternate-day corticosteroid regimen in children with active Crohn’s disease	Prematurely ended
8.	2010-022215-19	A Pharmacokinetic Study of The Combined Use Of Recombinant Human GH And IGF-1 In Children With Inflammatory Bowel (Crohn’s) Disease	Prematurely ended
9.	2012-005644-26	Administration of intranvenous ferric carboxymaltose to children with IBD	Observational study
10.	2015-003293-32	Multicentre, Randomised, Placebo-Controlled Trial of Mebeverine in Children with Irritable Bowel Syndrome (IBS) or Functional Abdominal Pain - not otherwise specified (FAP-NOS)	Non-IBD/peripheral treatment
11.	2015-005753-12	Prospective, open-label, randomised pilot study to assess two possible routes of Faecal Microbiota Transplant (FMT) delivery in patients with ulcerative colitis.	Withdrawn
12.	2016-000786-24	Thalidomide, a novel immunological treatment to modify the natural history of paediatric Crohns disease: a new proposal from a well-established paediatric research network	Prematurely ended
13.	2016-002265-60	Efficacy, safety and tolerability of a bowel cleansing preparation (Eziclen®/Izinova®) in paediatric subjects undergoing colonoscopy: a phase iii, multicentre, randomised, comparative study versus	Non-IBD/peripheral treatment
14.	2018-002001-65	A Phase Ib/IIb, Randomised, Double Blind, Placebo-Controlled Trial to Investigate the Safety, Tolerability and Clinical Activity of Humanised Antibody GSK1070806 in the Treatment of Patients with M	Withdrawn
15.	2018-004346-42	A 52-Week, Multicenter, Randomized, Double-blind, Double-dummy, Placebo and Active-controlled, Operationally Seamless Phase 2b/3, Parallel-group Study to Assess the Efficacy and Safety of Brazikumab	Withdrawn/ terminated
16.	2019-001298-96	Protocol I6T-MC-AMBU A Mulitcenter, Open-Label PK Study of Mirikizumab in Pediatric Patients with Moderately to Severely Active Ulcerative Colitis	Duplicate study
17.	2019-001866-14	An Open-label, Long-term Extension Study of Brazikumab in Participants with Moderately to Severely Active Crohn’s Disease	Withdrawn/ terminated
18.	2019-004219-29	‚Personalized AZithromycin/metronidAZole, in combination with standard induction therapy, to achieve a fecal microbiome community structure and metagenome changes associated with sustained remission in pediatric Crohn’s Disease (CD): a pilot study	Insufficient data
19.	2019-004224-38	A Phase 3 Study of the Efficacy, Safety and Pharmacokinetics of Ustekinumab as Open-label Intravenous Induction Treatment Followed by Randomized Double-blind Subcutaneous Ustekinumab Maintenance in...	Duplicate study
20.	2020-003193-48	A Phase 3, Open-Label, Multicenter Study to Evaluate the Efficacy and Safety of Darvadstrocel in the Treatment of Complex Perianal Fistula in Pediatric Subjects with Crohn‚Äôs Disease over a Period o...	Duplicate study
21.	2020-004301-31	A Randomized, Double-Blind, Phase 3 Study to Evaluate the Efficacy and Safety of Vedolizumab Intravenous as Maintenance Therapy in Pediatric Subjects with Moderately to Severely Active Crohn‚Äôs Dise...	Duplicate study

No.#	EudraCT Number	Title	Reason for exclusion
22.	2006-000410-20	A Phase 3, Randomized, Open-label, Parallel-group, Multicenter Trial to Evaluate the Safety and Efficacy of Infliximab (REMICADE ®) in Pediatric Subjects with Moderately to Severely Active Ulcerative Colitis	Duplicate study
23.	2006-004814-41	A Multicenter, Double-Blind Study to Evaluate the Safety, Efficacy and Pharmacokinetics of the Human Anti-TNF Monoclonal Antibody Adalimumab in Pediatric Subjects with Moderate to Severe Crohn's Disease	Duplicate study
24.	2007-006494-90	A Multi-center, Open-label Study of the Human Anti−TNF Monoclonal Antibody Adalimumab to Evaluate the Efficacy and the Long-term Safety and Tolerability of Repeated Administration of Adalimumab in Pediatric Subjects with Crohn's Disease Who Have Demonstrated a Clinical Response in the M06-806 Study	Duplicate study
25.	2011-000164-10	A Phase 1, Multicenter, Open-label Study to Determine the Safety and Pharmacokinetics of MMX Mesalamine Following Administration in Children and Adolescents With Ulcerative Colitis	Duplicate study
26.	2011-003743-22	A Multicenter, Open label, Non-Comparative Study to Evaluate the Safety of Entocort EC for the Treatment of Crohn's Disease in Paediatric Subjects Aged 5 to 17 Years, Inclusive	Duplicate study
27.	2012-004366-18	A Phase 1b Open-Label Study to Assess the Safety and Pharmacokinetics of Subcutaneously Administered Golimumab, a Human Anti-TNFα Antibody, in Pediatric Subjects With Moderately to Severely Active Ulcerative Colitis	Duplicate study
28.	2012-004851-35	Once versus twice daily mesalazine to introduce remission in pediatric ulcerative colitis: a randomized controlled trial	Duplicate study
29.	2012-005226-29	Azithromycin Based Therapy for Induction of Remission in Active Pediatric Crohn's Disease	Duplicate study
30.	2013-001744-65	A Phase 3, Multicenter, Randomized, Double-blind Study to Determine the Safety and Efficacy of MMX Mesalamine/Mesalazine in Pediatric Subjects with Mild to Moderate Ulcerative Colitis, in Both Acute and Maintenance Phases	Duplicate study
31.	2013-003032-77	A Multicenter, Randomized, Double-Blind Study of the Human Anti-TNF Monoclonal Antibody Adalimumab in Pediatric Subjects with Moderate to Severe Ulcerative Colitis.	Duplicate study
32.	2014-004381-24	A Phase 2, Open-label, Multicenter Study to Assess the Safety and Efficacy of Certolizumab Pegol in Children and Adolescents With Active Crohn's Disease (NURTURE Study)	Duplicate study
33.	2014-004531-39	A Multi-Center, Randomized, Double-blind, Placebo-controlled Study of Adalimumab for the Maintenance of Clinical Remission in Japanese Subjects With Crohn's Disease.	Duplicate study
34.	2014-005666-29	A Phase 1 randomised, double-blind, placebo-controlled study to assess the safety and tolerability of Bacteroides thetaiotaomicron in young people aged 16 to 18 years with stable Crohn's disease.	Duplicate study
35.	2015-001653-32	A Phase 4, Multicenter, Open-label Study of Serum Infliximab Concentrations and Efficacy and Safety of Dose Escalation in Pediatric Patients with inflammatory Bowel Disease	Duplicate study
36.	2015-001963-37	A Phase 3, long-term active treatment extension study of mongersen (GED-0301) in subjects with Crohn’s disease	Duplicate study
37.	2015-004121-13	A Multicenter Open-label Study of the Human Anti-TNF Monoclonal Antibody Adalimumab to Investigate Efficacy, Safety and Pharmacokinetics after Dose Escalation in Japanese Subjects with Crohn's Disease	Duplicate study
38.	2016-000641-31	A Multicenter, Randomized, Double-Blind, Placebo-Controlled Study to Evaluate the Safety and Efficacy of Upadacitinib (ABT-494) for Induction and Maintenance Therapy in Subjects With Moderately to Severely Active Ulcerative Colitis	Duplicate study
39.	2016-000642-62	A Multicenter, Randomized, Double-Blind, Placebo-Controlled Induction Study to Evaluate the Efficacy and Safety of Upadacitinib (ABT-494) in Subjects with Moderately to Severely Active Ulcerative Colitis	Duplicate study
40.	2016-001956-22	A Randomized Double-blind Pharmacokinetic study of Ustekinumab in Pediatric Subjects with Moderately to Severely Active Crohn's Disease	Duplicate study
41.	2017-002231-41	A Phase 2, Randomized, Double-Blind, Dose-Ranging Study to Determine the Pharmacokinetics, Safety and Tolerability of Vedolizumab IV in Pediatric Subjects with Ulcerative Colitis or Crohn’s Disease	Duplicate study
42.	2017-005025-20	An Open-label, Multicenter Study to Assess the Safety of Certolizumab Pegol in Children and Adolescents with Active Crohn's Disease Who Completed C87035 or Were Terminated from C87035 when the Study Was Stopped by UCB	Duplicate study

No.#	EudraCT Number	Title	Reason for exclusion
43.	2018-003985-15	A Phase 3, Randomized, Double-Blind, Placebo-Controlled, 52-Week Study to Assess the Efficacy and Safety of Etrasimod in Subjects with Moderately to Severely Active Ulcerative Colitis	Duplicate study
44.	2018-003986-33	A Phase 3, Randomized, Double Blind, Placebo Controlled, 12 Week Study to Assess the Efficacy and Safety of Etrasimod in Subjects with Moderately to Severely Active Ulcerative Colitis	Duplicate study
45.	2019-004225-24	A Phase 3 Study of the Efficacy, Safety, and Pharmacokinetics of Ustekinumab as Open-label Intravenous Induction Treatment Followed by Randomized Double-blind Subcutaneous Ustekinumab Maintenance in Pediatric Participants with Moderately to Severely Active Crohn's Disease	Duplicate study
46.	2017-003649-10	A Phase I, Open–Label, Randomized, Pharmacokinetic, Pharmacodynamic, And Safety Study Of Etrolizumab Followed By Open–Label Extension And Safety Monitoring In Pediatric Patients From 4 Years To Less Than 18 Years Of Age With Moderate To Severe Ulcerative Colitis Or Moderate To Severe Crohn's Disease	Duplicate study
47.	2007-005702-49	Pilot study on the efficacy of golimumab in the induction and maintenance of clinical and endoscopic remission in patients with steroid-dependent ulcerative colitis.	Duplicate study
48.	2004-002802-31	Multicentre, Controlled, Randomised, Open, Cross-over Study Comparing the Acceptability of Pentasa® Sachets versus Pentasa® tbl. 500mg in Children with Crohn´s Disease	Duplicate study
49.	2011-003742-40	A Multicenter, Open label, Non-Comparative Study to Evaluate the Safety of Entocort™ EC as a Maintenance Treatment for Crohn's Disease in Paediatric Subjects Aged 5 to 17 Years, Inclusive	Duplicate study
50.	2015-001346-29	A Multi-Center, Open-Label Study of Adalimumab to Evaluate Long-Term Safety and Tolerability of Repeated Administration of Adalimumab in Pediatric Subjects with Ulcerative Colitis Who Completed the Study M11-290	Duplicate study
51.	2017-002182-21	A Phase 2b, Extension Study to Determine the Long-term Safety of Vedolizumab IV in Pediatric Subjects With Ulcerative Colitis or Crohn's Disease.<br>(Long-term Safety With Vedolizumab IV in Pediatric Subjects With Ulcerative Colitis or<br>Crohn's Disease)	Duplicate study
52.	2017-004496-31	A Phase 3 Randomized, Open-Label Study to Assess the Efficacy, Safety,and Pharmacokinetics of Golimumab Treatment, a Human anti-TNFα Monoclonal Antibody, Administered Subcutaneously in Pediatric Participants with Moderately to Severely Active Ulcerative Colitis	Duplicate study
53.	2020-004300-34	A Randomized, Double-Blind, Phase 3 Study to Evaluate the Efficacy and Safety of Vedolizumab Intravenous as Maintenance Therapy in Pediatric Subjects with Moderately to Severely Active Ulcerative Colitis Who Achieved Clinical Response Following Open–Label Vedolizumab Intravenous Therapy	Duplicate study
54.	2020-005311-49	Master protocol for a Phase 3, multicenter, open-label, long-term extension study to evaluate the long-term efficacy and safety of mirikizumab in children and adolescents with moderate-to-severe ulcerative colitis or Crohn's disease	Duplicate study
55.	2021-000630-34	A Phase 3b Extension Study to Evaluate the Long-term Safety of Vedolizumab Intravenous in Pediatric Patients With Ulcerative Colitis or Crohn's Disease	Duplicate study
56.	2021-003220-32	Impact of early proactive therapeutic drug monitoring on the durability and efficacy of infliximab therapy in pediatric inflammatory bowel disease: a multicenter open-label randomized-control trial.	Duplicate study
57.	2021-006282-37	A Phase 3, Multicenter, Randomized, Platform Study of p19 Inhibition of the IL-23 Pathway to Establish Efficacy in Pediatric Crohn's Disease	Duplicate study
58.	2022-000811-29	A Phase 3, Multicenter, Randomized, Platform Study of p19 Inhibition of the IL-23 Pathway to Establish Efficacy in Pediatric Crohn's Disease (MACARONI-23)	Duplicate study
59.	2022-002648-35	Prospective Analysis of Pharmacokinetic Infliximab Data in pediatric CD patients	Duplicate study
60.	2020-004457-76	A Phase 3, Multicenter, Open-label, Basket, Long-term Extension Study of Ustekinumab in Pediatric Clinical Study Participants (2 to <18 Years of Age)	Duplicate study
61.	2016-000674-38	A Phase 3 Multicenter, Long-Term Extension Study to Evaluate the Safety and Efficacy of Upadacitinib (ABT-494) in Subjects with Ulcerative Colitis (UC)	Duplicate study
62.	2018-002378-30	Open-Label Induction And Maintenance Study Of Oral Cp-690,550 (Tofacitinib) In Children With Moderately To Severely Active Ulcerative Colitis	Duplicate study
63.	2018-003987-29	An Open-Label Extension Study of Etrasimod in Subjects with Moderately to Severely Active Ulcerative Colitis	Duplicate study

No.#	EudraCT Number	Title	Reason for exclusion
64.	2021-002308-11	A phase 2/3, multicenter, randomized, double-blind study to evaluate the efficacy, safety, pharmacokinetics and pharmacodynamics of oral ozanimod (RPC1063) in pediatric subjects with moderately to severely active ulcerative colitis with an inadequate response to conventional therapy.	Duplicate study
65.	2021-005019-30	A Phase 2/3, Multicenter, Randomized, Double-blind Study to Evaluate the Efficacy, Safety, Pharmacokinetics and Pharmacodynamics of Oral Ozanimod (RPC1063) in Pediatric Participants with Moderately to Severely Active Crohn's Disease with an Inadequate Response to Conventional Therapy	Duplicate study
66.	2020-000311-71	Pilot study of a new technique of Oral Fecal Transplantation using frozen stool capsules for the maintenance treatment of UC with pediatric onset	Duplicate study
67.	2015-001900-76	Manipulating the microbiome in IBD by antibiotics and fecal microbiota transplantation (FMT): a randomized controlled trial	Duplicate study

Supplementary Table 2: Characteristics of Completed Trials in Paediatric IBD

Intervention category	Study ID	Intervention	FDA/EMA approval (if applicable)					Study design	Comparator (if applicable)	Initial enrolment	Primary outcome measure(s)	Secondary outcome measure (s)	Age	Population characteristics	Treatment phase	Sponsor	Location	Single- vs multi-centre	Target or actual sample size	Recruitment status	Results (yes/no)
			Indication	FDA – adult	FDA – paediatric	EMA – adult	EMA – paediatric														
Biologic agents (RCTs)	1. NCT00445939	Adalimumab<br>*Anti-TNF*	CD	27 Feb 2007<br>*Humira*	23 Sep 2014<br>*Humira*	04 Jun 2007<br>*Humira*	22 Nov 2012<br>*Humira*	Parallel, quadruple-blind, phase 2/3	Placebo	Feb 2007	Clinical remission (CDAI<150) at week 4	1. CDAI Remission (CDAI<150) at week 2, 6, and 8<br>2. Clinical response in period A (weeks 2 and 4)<br>3. Clinical response in period B (weeks 6 and 8)	15 - 75	1. CD with CDAI between 220 and 450<br>2. If previously used infliximab, discontinued due to loss of response or intolerance	Induction	Industry	Japan	Multi-centre	90	Completed	Yes
			UC	28 Sep 2012<br>*Humira*	24 Feb 2021<br>*Humira*	04 Apr 2012<br>*Humira*	20 Nov 2020<br>*Humira*														
	2. NCT00445432	Adalimumab<br>*Anti-TNF*	CD	27 Feb 2007<br>*Humira*	23 Sep 2014<br>*Humira*	04 Jun 2007<br>*Humira*	22 Nov 2012<br>*Humira*	Parallel, quadruple-blind, phase 2/3	Placebo	Mar 2007	Clinical remission (CDAI<150) at week 52	1. 70-point response at week 52<br>2. 100-point response at week 52<br>3. Change in CDAI<br>4. Change in IOIBD<br>5. Change in IBDQ<br>6. Change in SF-36	15 - 75	CD patients successfully enrolled in and completed related study NCT00445939	Maintenance	Industry	Japan	Multi-centre	82	Completed	Yes
			UC	28 Sep 2012<br>*Humira*	24 Feb 2021<br>*Humira*	04 Apr 2012<br>*Humira*	20 Nov 2020<br>*Humira*														
	3. NCT00409682	Adalimumab (dose regimen 1)<br>*Anti-TNF*	CD	27 Feb 2007<br>*Humira*	23 Sep 2014<br>*Humira*	04 Jun 2007<br>*Humira*	22 Nov 2012<br>*Humira*	Parallel, quadruple-blind, phase 3	Adalimumab (dose regimen 2)	Apr 2007	Clinical remission (PCDAI≤10) at week 26	1. Clinical remission at week 52<br>2. Clinical response (decrease in PCDAI at least 15 points) at week 26<br>3. IMPACT III at week 26 and 52	6 - 17	CD diagnosis for at least 12 weeks with PCDAI > 30 despite concurrent oral corticosteroid/AZA/6-MP/MTX treatment	Induction and maintenance	Industry	Belgium, Canada, Czech Republic, France, Netherlands, Poland, UK, US	Multi-centre	192	Completed	Yes
			UC	28 Sep 2012<br>*Humira*	24 Feb 2021<br>*Humira*	04 Apr 2012<br>*Humira*	20 Nov 2020<br>*Humira*														
	4. NCT00853099	Adalimumab<br>*Anti-TNF*	CD	27 Feb 2007<br>*Humira*	23 Sep 2014<br>*Humira*	04 Jun 2007<br>*Humira*	22 Nov 2012<br>*Humira*	Parallel, double-blind, phase 3	Placebo	Feb 2009	1. Clinical remission (MS ≤2 with no individual subscore >1) at 8 weeks<br>2. Clinical remission at 52 weeks	1. Percentage of patients with clinical remission at 8, 32 and 52 weeks<br>2. Clinical response<br>3. Mucosal healing<br>4. Rectal bleeding subscore<br>5. PGA subscore<br>6. Stool frequency subscore<br>7. AEs	15+	Active UC with MS of 6-12 at baseline despite concurrent treatment with (1) oral CS or immunosuppressants or both or (2) AZA or 6-MP	Induction	Industry	Japan	Multi-centre	274	Completed	Yes
			UC	28 Sep 2012<br>*Humira*	24 Feb 2021<br>*Humira*	04 Apr 2012<br>*Humira*	20 Nov 2020<br>*Humira*														
	5. NCT02065557	Adalimumab<br>*Anti-TNF*	CD	27 Feb 2007<br>*Humira*	23 Sep 2014<br>*Humira*	04 Jun 2007<br>*Humira*	22 Nov 2012<br>*Humira*	Parallel, quadruple-blind, phase 3	Placebo	Oct 2014	Clinical remission as per pMS and MS	1. Clinical response per MS<br>2. Mucosal healing at week 52<br>3. Clinical remission per MS at week 52<br>4. CS-free clinical remission per MS and pMS	4 - 17	Active UC with MS of 6-12 despite concurrent treatment with oral CS or immunosuppressants or both	Induction and maintenance	Industry	Australia, Austria, Belgium, Canada, Czechia, Hungary, Israel, Japan, New Zealand, Poland, Slovakia, Spain, UK, US	Multi-centre	101	Completed	Yes
			UC	28 Sep 2012<br>*Humira*	24 Feb 2021<br>*Humira*	04 Apr 2012<br>*Humira*	20 Nov 2020<br>*Humira*														

Intervention category	Study ID	Intervention	FDA/EMA approval (if applicable)					Study design	Comparator (if applicable)	Initial enrolment	Primary outcome measure(s)	Secondary outcome measure (s)	Age	Population characteristics	Treatment phase	Sponsor	Location	Single- vs multi-centre	Target or actual sample size	Recruitment status	Results (yes/no)
			Indication	FDA – adult	FDA – paediatric	EMA – adult	EMA – paediatric														
	6.<br>NCT02256462<br>*The PAILOT Trial*	Adalimumab<br>*Anti-TNF*	CD	27 Feb 2007<br>*Humira*	23 Sep 2014<br>*Humira*	04 Jun 2007<br>*Humira*	22 Nov 2012<br>*Humira*	Parallel, open-label, phase 4	None	May 2015	Loss of response during treatment	1. CS-free complete clinical remission<br>2. Trough levels<br>3. ADA antibodies<br>4.Anthropometrics<br>5. Laboratory markers<br>6. AEs<br>7. Need for treatment modification<br>8. PCDAI	6 - 17	1. CD diagnosis<br>2. Biologic naïve<br>3. Negative TB test, HBV-S Ag, stool culture, parasites, *C. difficile* toxin	Induction and maintenance	Institutional grant	Israel	Single-centre	82	Completed	Yes
			UC	28 Sep 2012<br>*Humira*	24 Feb 2021<br>*Humira*	04 Apr 2012<br>*Humira*	20 Nov 2020<br>*Humira*														
	7.<br>NCT00291668	Certolizumab pegol<br>*Anti-TNF*	CD	22 Apr 2008<br>*Cimzia*	Not approved for paediatric use	Authorization refused on Nov 17, 2007 on the basis of insufficient evidence supporting the benefits and safety concerns of Cimzia (subcutaneous certolizumab pegol)		Parallel, double-blind, phase 2	Placebo	Mar 2006	Clinical response (decrease in CDAI of 100 points from baseline) or remission (CDAI<150) at week 6	1. CDAI at weeks 2,4 and 6<br>2. 100-point response at weeks 2 and 4<br>3. 70-point response at weeks 2,4, and 6<br>4. Clinical remission at weeks 2,4 and 6<br>5. IBDQ at weeks 2, 4 and 6<br>5. CRP at weeks 2, 4 and 6	16 - 65	1. CD diagnosis at least 24 weeks prior with CDAI between 220 and 450<br>2. CRP of 1 mg/dL or higher at the start	Induction	Industry	Japan	Multi-centre	94	Completed	Yes
	8.<br>NCT03681067<br>*The CDAID Trial*	GSK1070806<br>*Anti-IL-18*	CD	Not approved for adult use	Not approved for paediatric use	Not approved for adult use	Not approved for paediatric use	Parallel, quadruple-blind, phase 1/2	Placebo	Feb 2019	1. AEs and SAEs<br>2. Clinical lab – haematology and biochemistry panel<br>3. Infections<br>4. Heart rate<br>5. Blood pressure<br>6. Body temperature<br>7. Respiratory rate<br>8. ECGs	CDAI score over time	16+	Moderate to severe CD with endoscopic evidence of active disease	Induction	Industry	UK	Single-centre	5	Completed	No
	9.<br>NCT00554710	Infliximab (in combination with AZA)<br>*Anti-TNF*	CD	24 Aug 1998<br>*Remicade*	19 May 2006<br>*Remicade*	13 Aug 1999<br>*Remicade*	30 May 2007<br>*Remicade*	Parallel, double-blind, phase 4	Standard treatment (corticosteroids)	May 2001	Remission without CS and without surgical resection (month 6 and 12)	1. Time to relapse<br>2. Proportion receiving infliximab, methylprednisolone, antimetabolites<br>3. Median serum C-reactive protein concentration<br>4. Proportion experiencing AE<br>5. Mean endoscopic severity scores and proportion of patients without ulcers	16 - 75	Active CD (CDAI>200) with no previous treatment with CS, antimetabolites, or biologic agents	Induction and maintenance	Industry	Belgium	Single-centre	129	Completed	No
			UC	16 Sep 2005<br>*Remicade*	23 Sep 2011<br>*Remicade*	26 Jan 2006<br>*Remicade*	21 Feb 2012<br>*Remicade*														

Intervention category	Study ID	Intervention	FDA/EMA approval (if applicable)					Study design	Comparator (if applicable)	Initial enrolment	Primary outcome measure(s)	Secondary outcome measure (s)	Age	Population characteristics	Treatment phase	Sponsor	Location	Single- vs multi-centre	Target or actual sample size	Recruitment status	Results (yes/no)
			Indication	FDA – adult	FDA – paediatric	EMA – adult	EMA – paediatric														
	10. NCT00207675	Infliximab (5 mg/kg)<br>*Anti-TNF*	CD	24 Aug 1998<br>*Remicade*	19 May 2006<br>*Remicade*	13 Aug 1999<br>*Remicade*	30 May 2007<br>*Remicade*	Double-blind (first year), open-label, phase 3	Infliximab (10 mg/kg)	Feb 2003	Clinical response at week 10 (≥ 15-point decrease in PCDAI with a total score ≤ 30 points)	Clinical response at week 54<br>Clinical remission at week 54<br>CS use at week 54<br>Change in height status at week 54	6 - 17	1. CD diagnosis with gastritis, duodenitis, colitis, ileitis, or ileocolitis<br>2. Active CD despite adequate ongoing immunomodulator treatment	Induction and maintenance	Industry	NA	Multi-centre	112	Completed	Yes
			UC	16 Sep 2005<br>*Remicade*	23 Sep 2011<br>*Remicade*	26 Jan 2006<br>*Remicade*	21 Feb 2012<br>*Remicade*														
	11. NCT00336492	Infliximab (5 or 10 mg/kg)<br>*Anti-TNF*	CD	24 Aug 1998<br>*Remicade*	19 May 2006<br>*Remicade*	13 Aug 1999<br>*Remicade*	30 May 2007<br>*Remicade*	Parallel, open-label, phase 3	Infliximab (different dosing or infusion regimens)	Sep 2006	Clinical response (decease in MS ≥ 3 points and rectal bleeding score of 0 or 1) at week 8	Clinical remission at week 54 (PUCAI<10)	6 - 17	Moderate to severe UC diagnosis for at least 2 weeks before start	Induction and maintenance	Industry	Belgium, Canada, Denmark, Netherlands, US	Multi-centre	60	Completed	Yes
			UC	16 Sep 2005<br>*Remicade*	23 Sep 2011<br>*Remicade*	26 Jan 2006<br>*Remicade*	21 Feb 2012<br>*Remicade*														
	12. NCT01346826	Infliximab<br>*Anti-TNF*	CD	24 Aug 1998<br>*Remicade*	19 May 2006<br>*Remicade*	13 Aug 1999<br>*Remicade*	30 May 2007<br>*Remicade*	Parallel, double-blind, phase 4	Infliximab (standard dosing)	May 2011	Total numbers of infusion reactions related with infliximab infusion	Numbers of severe infusion reactions related with infliximab infusion	16 - 80	CD or UC patients receiving infliximab and showing no reactions during four 2-hour 5 mg/kg infusions	Induction and maintenance	Institutional grant	South Korea	Single-centre	145	Completed	Yes
			UC	16 Sep 2005<br>*Remicade*	23 Sep 2011<br>*Remicade*	26 Jan 2006<br>*Remicade*	21 Feb 2012<br>*Remicade*														
	13. NCT03926130	Mirikizumab<br>*Anti-IL-23p19*	CD	15 Jan 2025<br>*Omvoh*	*Approval pending*	12 Feb 2025<br>*Omvoh*	*Approval pending*	Parallel, triple-blind, phase 3	Ustekinumab, placebo	Jul 2019	1. Clinical Response at Week 12 and Endoscopic Response at Week 52<br>2. Clinical Response at Week 12 and Clinical Remission at Week 52	1. Endoscopic Response<br>2. Endoscopic Remission<br>3. Clinical remission<br>4. Change from Baseline in Urgency Numeric Rating Score (NRS)<br>5. CRP<br>6. FCal<br>7. Fistulae response<br>8. EIMs<br>9. PK<br>10. Health related QOL	15-80	1. Moderate to severe CD<br>2. Intolerance/loss of response/inadequate response to conventional CD therapies<br>3. Female- meet contraception recommendations	Induction and maintenance	Industry	Argentina, Australia, Austria, Belgium, Brazil, Canada, China, Croatia, Czechia, Denmark, France, Germany, Hungary, India, Israel, Italy, Japan, Korea, Latvia, Lithuania, Mexico, Netherlands, Poland, Puerto Rico, Romania, Russia, Serbia, Slovakia, Spain, Switzerland, Turkey, Ukraine, UK, USA	Multi-centre	1158	Completed	Yes
			UC	26 Oct 2023<br>*Omvoh*	*Approval pending*	26 May 2023<br>*Omvoh*	*Approval pending*														
	14. NCT03290781	Ontamalimab	CD/UC	Not approved for adult use	Not approved for	Not approved for adult use	Not approved for	Parallel, double-	Placebo	Apr 2018	Remission as per composite	1. Endoscopic remission<br>2. Clinical remission	16 - 81	1. Completed induction in related studies NCT03259334 and	Maintenance	Institutional grant	Multi-national	Multi-centre	366	Completed	Yes

Intervention category	Study ID	Intervention	FDA/EMA approval (if applicable)					Study design	Comparator (if applicable)	Initial enrolment	Primary outcome measure(s)	Secondary outcome measure (s)	Age	Population characteristics	Treatment phase	Sponsor	Location	Single- vs multi-centre	Target or actual sample size	Recruitment status	Results (yes/no)
			Indication	FDA – adult	FDA – paediatric	EMA – adult	EMA – paediatric														
	FIGARO UC 303	*Anti-MAdCAM-1*			paediatric use		paediatric use	blind, phase 3			score at week 52	3. Sustained remission<br>4. Clinical response<br>5. Mucosal healing<br>6.Glucocorticoid-free remission<br>7. Remission as per total MS<br>8. Sustained endoscopic remission<br>9. TEAEs<br>10. Positive antidrug antibodies		NCT03259308 and received clinical response (decrease in patient-reported symptoms and total MS)							
	15. NCT03104413	Risankizumab (via IV infusions and SC injections)<br><br>*Anti-IL-23p19*	CD<br><br>UC	16 Jun 2022 *Skyrizi*<br><br>18 Jun 2024 *Skyrizi*	Not approved for paediatric use<br><br>Not approved for paediatric use	21 Nov 2022 *Skyrizi*<br><br>24 Jul 2024 *Skyrizi*	Not approved for paediatric use<br><br>Not approved for paediatric use	Parallel, double-blind, phase 3	Placebo	Dec 2017	1. CDAI Clinical Remission<br>2. SES-CD Endoscopic Response	1. Clinical remission<br>2. CDAI Clinical Response and Remission (week 4 and week 12)<br>3. Change in FACIT<br>4. CDAI clinical response and endoscopic response<br>5. Stool frequency remission<br>6. Abdominal pain remission<br>7. Enhanced clinical response<br>8. Ulcer-free endoscopy<br>9. Resolution of EIMs (in participants with EIMs)<br>10 % CD-related hospitalizations<br>11. % without draining fistulas at week 12 (in participants with draining fistulas at week 0)	16 - 80	1. Moderate to severe CD diagnosis with CDAI of 220-450<br>2. Intolerance or inadequate response to biologic therapy	Induction	Industry	Multinational	Multi-centre	618	Completed	Yes
	16. NCT03105128	Risankizumab (via IV infusions and SC injections)<br><br>*Anti-IL-23p19*	CD	16 Jun 2022 *Skyrizi*	Not approved for paediatric use	21 Nov 2022 *Skyrizi*	Not approved for paediatric use	Parallel, double-blind, phase 3	Placebo	May 2017	1. CDAI Clinical remission<br>2. Endoscopic response	1. Clinical remission<br>2. CDAI clinical response and remission (week 4 and week 12)<br>3. Change in FACIT<br>4. CDAI clinical response and endoscopic response<br>5. Stool frequency remission	16 - 80	1. Moderate to severe CD diagnosis with CDAI of 220-450<br>2. Intolerance or inadequate response to biologic therapy	Induction	Industry	Multinational	Multi-centre	931	Completed	Yes

Intervention category	Study ID	Intervention	FDA/EMA approval (if applicable)					Study design	Comparator (if applicable)	Initial enrolment	Primary outcome measure(s)	Secondary outcome measure (s)	Age	Population characteristics	Treatment phase	Sponsor	Location	Single- vs multi-centre	Target or actual sample size	Recruitment status	Results (yes/no)
			Indication	FDA – adult	FDA – paediatric	EMA – adult	EMA – paediatric														
			UC	18 Jun 2024<br>*Skyrizi*	Not approved for paediatric use	24 Jul 2024<br>*Skyrizi*	Not approved for paediatric use					6. Abdominal pain remission<br>7. Enhanced clinical response<br>8. Ulcer-free endoscopy<br>9. Resolution of EIMs (in participants with EIMs)<br>10 CD-related hospitalizations<br>11. No draining fistulas at week 12 (in participants with draining fistulas at week 0)									
	17. NCT02968108	Ustekinumab<br>*Anti-IL12-and-IL23*	CD	23 Sep 2016<br>*Stelara*	15 Apr 2026<br>*Stelara*	11 Nov 2016<br>*Stelara*	31 Mar 2025<br>*Stelara*	Parallel, double-blind, phase 1	Alternate dosing regimen	Jan 2017	Serum USTE concentrations over time	1. Clinical response as per PCDAI<br>2. Clinical remission as per PCDAI	2 - 17	1. Active moderate to severe CD with colitis, ileitis, or ileocolitis and baseline PCDAI>30<br>2. Prior or concurrent treatment with oral CS/AZA/6-MP/MTX<br>3. Negative stool tests for enteric pathogens	Induction	Industry	Belgium, Canada, France, Germany, Poland, US	Multi-centre	45	Completed	No
			UC	18 Oct 2019<br>*Stelara*	*Approval pending*	03 Sep 2019<br>*Stelara*	*Approval pending*														
	18. NCT02038920	Vedolizumab<br>*Anti-integrin-α4β7*	CD/UC	20 May 2014<br>*Entyvio*	*Approval pending*	22 May 2014<br>*Entyvio*	*Approval pending*	Parallel, quadruple-blind, phase 3	Placebo	Jan 2014	1. CDAI-100 Response<br>2. Clinical Remission<br>3. AEs or TEAEs<br>4. TEAE related to weight, vital signs, ECGs<br>5. Abnormal laboratory parameters	1. CDAI<150<br>2. Change in CRP<br>3. Durable remission: CDAI<150<br>4. CS-free remission: CDAI<150<br>5. Serum vedo concentration<br>6. AVA<br>7. Neutralizing AVA	15 - 80	Small-intestinal, large-intestinal, or small-/large-intestinal CD diagnosis with baseline CDAI of 220-450	Induction and maintenance	Industry	Japan	Multi-centre	157	Completed	Yes
	19. NCT02039505	Vedolizumab<br>*Anti-integrin-α4β7*	CD/UC	20 May 2014<br>*Entyvio*	*Approval pending*	22 May 2014<br>*Entyvio*	*Approval pending*	Parallel, quadruple-blind, phase 3	Placebo	Feb 2014	1. Clinical response at week 10<br>2. Clinical remission at week 60<br>3. AEs or TEAEs<br>4. TEAE related to weight, vital signs, ECGs<br>5. Abnormal laboratory parameters	1. Clinical remission at week 10<br>2. Mucosal healing at week 10<br>3. Durable clinical response at week 10 and week 60<br>4. Mucosal healing at week 60<br>5. Durable clinical remission at week 10 and week 60<br>6. CS-free clinical remission at week 60<br>7. Serum vedolizumab concentration from week 2 to week 60<br>8. Neutralizing AVA	15 - 80	1. Total or left-sided moderate to severe UC with baseline MS of 6-12<br>2. Resistance, intolerance, or loss of response to CS, immunomodulators, or anti-TNF	Induction and maintenance	Industry	Japan	Multi-centre	292	Completed	Yes

Intervention category	Study ID	Intervention	FDA/EMA approval (if applicable)					Study design	Comparator (if applicable)	Initial enrolment	Primary outcome measure(s)	Secondary outcome measure (s)	Age	Population characteristics	Treatment phase	Sponsor	Location	Single- vs multi-centre	Target or actual sample size	Recruitment status	Results (yes/no)
			Indication	FDA – adult	FDA – paediatric	EMA – adult	EMA – paediatric														
	20.<br>NCT03138655	Vedolizumab<br><br>*Anti-integrin-α4β7*	CD/UC	20 May 2014<br><br>*Entyvio*	*Approval pending*	22 May 2014<br><br>*Entyvio*	*Approval pending*	Parallel, triple-blind, phase 2	Alternate dosing regimen	Nov 2017	1. Area under serum concentration-time curve at week 14<br>2. Average serum concentration during a dosing interval at week 14<br>3. Observed serum concentration at the end of a dosing interval at week 14	1. Percentage UC participants with clinical response as per complete MS<br>2. Percentage CD participants with clinical response as per CDAI	2 - 17	1. Moderate to severe UC extending proximal to the rectum with complete MS of 6-12<br>2. Moderate to severe CD involving the ileum and/or colon with SES-CD>7<br>3. Inadequate response, loss of response, or intolerance to corticosteroids, immunomodulators, or anti-TNF	Induction	Industry	Belgium,<br>Canada,<br>France,<br>Germany,<br>Hungary,<br>Israel,<br>Netherlands,<br>Poland,<br>Ukraine,<br>UK,<br>US	Multi-centre	89	Completed	Yes
	21.<br>NCT03196427	Vedolizumab<br><br>*Anti-integrin-α4β7*	CD/UC	20 May 2014<br><br>*Entyvio*	*Approval pending*	22 May 2014<br><br>*Entyvio*	*Approval pending*	Parallel, double-blind, phase 2	Alternate dosing regimen	Jul 2018	Treatment-emergent Adverse Events (TEAEs)	1. Complete MS clinical response<br>2. SES-CD and CDAI clinical response<br>3. Time to major IBD-related events<br>4. Change in IMPACT-3 scores<br>5. Change in height, weight, BMI<br>6. Tanner Stage V achievement	2-17	Moderate to severe UC or CD who completed NCT03138655 study and achieved clinical response at week 22	Maintenance	Industry	France,<br>Hungary<br>Israel,<br>Poland,<br>Ukraine, UK<br>, US	Multi-centre	59	Completed	No
	25.<br>NCT04779307	Vedolizumab<br><br>*Anti-Integrin-α4β7*	CD/UC	20 May 2014<br><br>*Entyvio*	*Approval pending*	22 May 2014<br><br>*Entyvio*	*Approval pending*	Parallel, quadruple-blind, phase 3	Alternate dosing regimen	Oct 2021	Clinical remission (mMS)	1. Clinical remission/ response (mMS, pMS, MS)<br>2. Sustained clinical remission/ response<br>3. Sustained endoscopic remission<br>4. Endoscopic response<br>5. CS-free clinical remission<br>6. Serum trough concentration<br>7. AVA and positive neutralizing AVA<br>8. AEs, SAEs, AESIs<br>9. Change in weight, linear growth Z-score, Tanner Stage	2-17	1. Moderate-severely active UC, unresponsive or intolerant to their current standard of care<br>2. Weighs ≥10 kg<br>3. Has failed, lost response to, or been intolerant to CS, AZA, 6-mercaptopurine, methotrexate, TNF-α antagonists<br>4. Has extensive colitis or pancolitis of >8 years' duration or left-sided colitis of >12 years' duration must have documented evidence of a negative surveillance colonoscopy within 12 months before screening	Induction and maintenance	Industry	Australia,<br>Belgium,<br>Canada,<br>China,<br>Croatia,<br>Greece,<br>Hungary,<br>Israel, Italy,<br>Japan,<br>Poland,<br>South Korea,<br>UK, United States	Multi-centre	121	Completed	No
	22.<br>NCT00267306	Visilizumab<br><br>*Anti-CD3*	CD/UC	Production as IBD medication halted post phase II/III trials due to inefficacy and poor safety profile				Parallel, open-label, phase 1/2	Alternate dosing regimen	Jan 2004	1. AEs<br>2. Tolerability	Not provided	16 - 70	Active UC despite ongoing steroid treatment	Induction	Industry	US	Multi-centre	144	Completed	Yes

Intervention category	Study ID	Intervention	FDA/EMA approval (if applicable)					Study design	Comparator (if applicable)	Initial enrolment	Primary outcome measure(s)	Secondary outcome measure (s)	Age	Population characteristics	Treatment phase	Sponsor	Location	Single- vs multi-centre	Target or actual sample size	Recruitment status	Results (yes/no)
			Indication	FDA – adult	FDA – paediatric	EMA – adult	EMA – paediatric														
	23. NCT04630028	Ustekinumab<br><br>*Anti-IL12-and-IL23*	CD	23 Sep 2016<br><br>*Stelara*	15 Apr 2026<br><br>*Stelara*	11 Nov 2016<br><br>*Stelara*	31 Mar 2025<br><br>*Stelara*	Parallel, double-blind, phase 3	Placebo	Mar 2021	1. Clinical remission (mMS)<br>2. Safety (AEs, SAEs, AEs leading to study discontinuation, AESIs, lab abnormalities)<br>3. PKs (serum ustekinumab concentration)	1. Clinical response (mMS)<br>2. Symptomatic remission<br>3. Clinical remission (PUCAI<10)<br>4. Endoscopic improvement<br>5. Histologic-endoscopic mucosal improvement<br>6. CS-free clinical remission	2-17	Moderate-severely active UC	Induction and maintenance	Industry	Belgium, Germany, Hungary, Israel, Japan, Poland, Russia, UK, US	Multi-centre	112	Completed	No
			UC	18 Oct 2019<br><br>*Stelara*	*Approval pending*	03 Sep 2019<br><br>*Stelara*	*Approval pending*														
	24. NCT04673357	Ustekinumab<br><br>*Anti-IL12-and-IL23*	CD	23 Sep 2016<br><br>*Stelara*	15 Apr 2026<br><br>*Stelara*	11 Nov 2016<br><br>*Stelara*	31 Mar 2025<br><br>*Stelara*	Parallel, double-blind, phase 3	Placebo	Apr 2021	1. Clinical remission (PCDAI<=10)<br>2. Safety (AEs, SAEs, AEs leading to study discontinuation, AESIs, lab abnormalities)<br>3. PKs (serum ustekinumab concentration)	1. Clinical remission (sPCDAI)<br>2. Clinical response<br>3. Endoscopic response (SES-CD)<br>4. CS-free clinical remission	2-17	1. CD or fistulizing CD with active colitis, ileitis, or ileocolitis, confirmed at any time in the past by endoscopy and histology<br>2. Moderate-severely active CD<br>3. If receiving enteral nutrition, must have been on a stable regimen for at least 2 weeks prior to induction week 0	Induction and maintenance	Industry	Belgium, Germany, Hungary, Israel, Japan, Poland, Russia, UK, US	Multi-centre	101	Completed	No
			UC	18 Oct 2019<br><br>*Stelara*	*Approval pending*	03 Sep 2019<br><br>*Stelara*	*Approval pending*														
	26. NCT03283085	Ontamalimab<br><br>*Anti-MAdCAM-1*	CD/UC	Not approved for adult use	Not approved for paediatric use	Not approved for adult use	Not approved for paediatric use	Parallel, double-blind, phase 3	Alternate dosing regimen	Feb 2018	1. TEAEs<br>2. Serious infections<br>3. Notable changes in clinical lab parameters, ECG, vital signs	1. Treatment response	16-80	1. Moderate-severe UC or CD<br>2. Enrolled in NCT03259334 or NCT03259308 (UC), or NCT03559517 or NCT03566823 (CD), and are in the treatment period of NCT03290781 (UC) or NCT03627091 (CD), completed the early termination or Week 52 visit in maintenance study (NCT03290781, NCT03627091), had responded to ontamalimab treatment (in the induction and/or maintenance studies)	Maintenance	Industry	Argentina, Australia, Austria, Belgium, Bosnia and Herzegovina, Bulgaria, Canada, Colombia, Croatia, Czechia, Estonia, Germany, Greece, Hungary, Ireland, Israel, Italy, Japan, Lebanon, Lithuania, Mexico, Netherlands, New Zealand, Poland, Portugal, Romania, Russia,	Multi-centre	557	Completed	Yes

Intervention category	Study ID	Intervention	FDA/EMA approval (if applicable)					Study design	Comparator (if applicable)	Initial enrolment	Primary outcome measure(s)	Secondary outcome measure (s)	Age	Population characteristics	Treatment phase	Sponsor	Location	Single- vs multi-centre	Target or actual sample size	Recruitment status	Results (yes/no)
			Indication	FDA – adult	FDA – paediatric	EMA – adult	EMA – paediatric														
																	Serbia, Slovakia, South Africa, South Korea, Spain, Switzerland, Turkey, Ukraine, UK, US				
	27. EudraCT: 2014-005702-37	Infliximab<br>*Anti-TNF*	CD	24 Aug 1998<br>*Remicade*	19 May 2006<br>*Remicade*	13 Aug 1999<br>*Remicade*	30 May 2007<br>*Remicade*	Parallel, open-label, phase 4	Other medicinal products	Feb 2015	Clinical remission	1. Clinical response and remission<br>2. Mucosal healing<br>3. Growth & QoL (IMPACT-III)<br>4. Therapy failure<br>5. AEs<br>6. Cumulative therapy use<br>7. Long-term remission, response, mucosal healing rates, flairs, AEs, complications, QoL	3-17	New-onset, untreated CD with moderate-to-severe disease activity (weighted PCDAI>40)	Induction and maintenance	Institutional grant	Belgium, Finland, Netherlands	Multi-centre	100	Completed	Yes
			UC	16 Sep 2005<br>*Remicade*	23 Sep 2011<br>*Remicade*	26 Jan 2006<br>*Remicade*	21 Feb 2012<br>*Remicade*														
Biologic agents (non-RCTs)	1. NCT00686374	Adalimumab<br>*Anti-TNF*	CD	27 Feb 2007<br>*Humira*	23 Sep 2014<br>*Humira*	04 Jun 2007<br>*Humira*	22 Nov 2012<br>*Humira*	Single-arm, open-label, phase 3	None	May 2008	1. PCDAI clinical remission<br>2. PCDAI clinical response	1. CDAI clinical remission<br>2. CDAI clinical response<br>3. Steroid-free PCDAI remission<br>4. Steroid-free CDAI remission<br>5. Mean change in PCDAI<br>6. Mean change in CDAI	7 to 18	CD patients must have successfully enrolled in and completed Study M06-806 through Week 52.<br>Participant must have been a responder	Maintenance	Industry	NA	Multi-centre	100	Completed	Yes
			UC	28 Sep 2012<br>*Humira*	24 Feb 2021<br>*Humira*	04 Apr 2012<br>*Humira*	20 Nov 2020<br>*Humira*														
	2. NCT01958827	Adalimumab<br>*Anti-TNF*	CD	27 Feb 2007<br>*Humira*	23 Sep 2014<br>*Humira*	04 Jun 2007<br>*Humira*	22 Nov 2012<br>*Humira*	Single-arm, open-label, phase 3	None	Sep 2013	Clinical response - CDAI	1. Clinical remission: CDAI<150<br>2. CR-50<br>3. CR-70<br>4. CR-100<br>5. CRP<br>6. Hematology parameters<br>7. Chemistry parameters<br>8. Systolic BP<br>9. Diastolic BP<br>10. Heart rate<br>11. Body temperature<br>12. AEs<br>13. Change in mean ADA serum concentration	15+	CD patients who received induction treatment with Humira® and achieved response, and then lost response during maintenance treatment; Elevated CRP at screening	Induction and maintenance	Industry	Japan	Multi-centre	28	Completed	Yes
			UC	28 Sep 2012<br>*Humira*	24 Feb 2021<br>*Humira*	04 Apr 2012<br>*Humira*	20 Nov 2020<br>*Humira*														

Intervention category	Study ID	Intervention	FDA/EMA approval (if applicable)					Study design	Comparator (if applicable)	Initial enrolment	Primary outcome measure(s)	Secondary outcome measure (s)	Age	Population characteristics	Treatment phase	Sponsor	Location	Single- vs multi-centre	Target or actual sample size	Recruitment status	Results (yes/no)
			Indication	FDA – adult	FDA – paediatric	EMA – adult	EMA – paediatric														
												14. Anti-ADA antibodies									
	3. NCT02632175	Adalimumab<br>*Anti-TNF*	CD<br>UC	27 Feb 2007 *Humira*<br>28 Sep 2012 *Humira*	23 Sep 2014 *Humira*<br>24 Feb 2021 *Humira*	04 Jun 2007 *Humira*<br>04 Apr 2012 *Humira*	22 Nov 2012 *Humira*<br>20 Nov 2020 *Humira*	Single-arm, open-label, phase 3	None	Nov 2015	1. AEs<br>2. Clinical remission (partial Mayo)<br>3. Clinical response (partial Mayo)<br>4. PUCAI remission<br>5. PUCAI response	Not provided	4-17	Moderate-severe UC who enrolled and completed M11-290 study (NCT02065557)	Maintenance	Industry	Japan, Poland, Slovakia, Spain, UK, US	Multi-centre	59	Completed	Yes
	4. NCT00329420	Certolizumab pegol<br>*Anti-TNF*	CD	22 Apr 2008<br>*Cimzia*	*Not approved for paediatric use*	Authorization refused on Nov 17, 2007 on the basis of insufficient evidence supporting the benefits and safety concerns of CIMZIA (subcutaneous certolizumab pegol)		Single-arm, open-label extension, phase 2	None	May 2006	Percentage of CDAI responders (achieving either clinical remission [CDAI ≤150] or clinical response [100-point reduction in CDAI from baseline]) at week 34	1. Change in CDAI at weeks 8, 10, 12, 14, 16, 20, 24, 28, 32, 34<br>2. CDAI responders at weeks 8, 12, 14, 16, 20, 24, 28, 32<br>3. Clinical remission<br>4. Time to disease progression<br>5. IBDQ<br>6. CRP<br>7. 70-point response on CDAI	16 to 64	CD patients who did not show clinical efficacy at Week 6 of the double-blind main study (NCT00291668) [reduction in CDAI score of ≥100 points from Week 0, or remission (CDAI ≤150)]	Induction and maintenance	Industry	Japan	Multi-centre	46	Completed	Yes
	5. NCT00329550	Certolizumab pegol<br>*Anti-TNF*	CD	22 Apr 2008<br>*Cimzia*	*Not approved for paediatric use*	Authorization refused on Nov 17, 2007 on the basis of insufficient evidence supporting the benefits and safety concerns of CIMZIA (subcutaneous certolizumab pegol)		Single-arm, open-label extension, phase 2	None	May 2006	Percentage of CDAI responders (achieving either clinical remission [CDAI ≤150] or clinical response [100-point reduction in CDAI from baseline]) at week 26	1. CDAI change at weeks 8, 12, 16, 20, 24, 26 or last visit<br>2. CDAI responders at weeks 8, 12, 16, 20, 24, 26 or last visit<br>3. Clinical remission<br>4. Time to disease progression<br>5. IBDQ<br>6. CRP<br>7. 70-point response on CDAI	16 to 64	CD patients who participated in main double-blind induction study C87037 (NCT00291668) and showed clinical efficacy	Maintenance	Industry	Japan	Multi-centre	40	Completed	Yes
	6. NCT01190410	Certolizumab pegol<br>*Anti-TNF*	CD	22 Apr 2008<br>*Cimzia*	*Not approved for paediatric use*	Authorization refused on Nov 17, 2007 on the basis of insufficient evidence supporting the benefits and safety concerns of CIMZIA (subcutaneous certolizumab pegol)		Parallel, open-label, phase 2	Alternate dosing regimen	Aug 2010	TEAEs	1. Discontinued treatment due to TEAE<br>2. Anti-nuclear antibodies<br>3. dsDNA antibodies<br>4. Clinical remission	6 to 17	CD patients who completed the C87035 study (NCT00899678) through Week 62 or assessments when their participation in C87035 was terminated when the study was stopped	Maintenance	Industry	Australia, Canada, US	Multi-centre	16	Completed	Yes

Intervention category	Study ID	Intervention	FDA/EMA approval (if applicable)					Study design	Comparator (if applicable)	Initial enrolment	Primary outcome measure(s)	Secondary outcome measure (s)	Age	Population characteristics	Treatment phase	Sponsor	Location	Single- vs multi-centre	Target or actual sample size	Recruitment status	Results (yes/no)
			Indication	FDA – adult	FDA – paediatric	EMA – adult	EMA – paediatric														
	7. NCT01900574	Golimumab<br>*Anti-TNF*	UC	15 May 2013<br>*Simponi*	07 Oct 2025<br>*Simponi*	19 Sep 2013<br>*Simponi*	19 Jan 2026<br>*Simponi*	Single-arm, open-label, phase 1	None	Aug 2013	Serum golimumab concentration	1. Clinical response<br>2. Clinical remission<br>3. PUCAI remission at weeks 6, 54, and 110<br>4. Mucosal healing	2 to 17	Moderate to severe UC (MS of 6-12 and endoscopic subscore ≥2); Current treatment with, or history of failing at least 1 of the following agents: oral or IV CS, 6-MP, AZA, or history of corticosteroid dependency	Induction and maintenance	Industry	Austria, Belgium, Canada, Denmark, France, Germany, Israel, Netherlands, Poland, US	Multi-centre	35	Completed	No
	8. NCT00805766	Infliximab<br>*Anti-TNF*	CD	24 Aug 1998<br>*Remicade*	19 May 2006<br>*Remicade*	13 Aug 1999<br>*Remicade*	30 May 2007<br>*Remicade*	Single-arm, open-label, phase 3	None	Dec 2008	Median CDAI change	1. CDAI<br>2. CDAI remission rates<br>3. CDAI change<br>4. TA-650 serum concentration<br>5. TA-650 antibody	16 to 75	Patients with CD who have relapsed in spite of maintenance treatment with 5mg/kg REMICADE, and who have an insufficient response to previous treatment	Induction and maintenance	Industry	Japan	Multi-centre	39	Completed	Yes
			UC	16 Sep 2005<br>*Remicade*	23 Sep 2011<br>*Remicade*	26 Jan 2006<br>*Remicade*	21 Feb 2012<br>*Remicade*														
	9. NCT01661257	Infliximab<br>*Anti-TNF*	CD	24 Aug 1998<br>*Remicade*	19 May 2006<br>*Remicade*	13 Aug 1999<br>*Remicade*	30 May 2007<br>*Remicade*	Single-arm, open-label, phase 3	None	Oct 2009	TIM-3 expression and correlation with PCDAI		13 to 17	Severe CD requiring infliximab treatment, Confirmed CD diagnosis based on clinical, endoscopic and histological findings	NA	Institutional grant	Republic of Korea	Single-centre	20	Completed	No
			UC	16 Sep 2005<br>*Remicade*	23 Sep 2011<br>*Remicade*	26 Jan 2006<br>*Remicade*	21 Feb 2012<br>*Remicade*														
	10. NCT01585155	Infliximab<br>*Anti-TNF*	CD	24 Aug 1998<br>*Remicade*	19 May 2006<br>*Remicade*	13 Aug 1999<br>*Remicade*	30 May 2007<br>*Remicade*	Single-arm, open-label, phase 3	None	Feb 2012	CAI remission	1. CAI score<br>2. pMS<br>3. PUCAI score	6 to 17	Patients with UC of at least 3 months duration prior to screening, Active UC despite adequate conventional therapy	Induction and maintenance	Industry	Japan	Multi-centre	21	Completed	Yes
			UC	16 Sep 2005<br>*Remicade*	23 Sep 2011<br>*Remicade*	26 Jan 2006<br>*Remicade*	21 Feb 2012<br>*Remicade*														
	11. NCT01580670	Infliximab<br>*Anti-TNF*	CD	24 Aug 1998<br>*Remicade*	19 May 2006<br>*Remicade*	13 Aug 1999<br>*Remicade*	30 May 2007<br>*Remicade*	Single-arm, open-label, phase 3	None	Mar 2012	PCDAI response	1. PCDAI score<br>2. Change in PCDAI<br>3. PCDAI remission rate<br>4. Serum TA-650 concentration<br>5. Percent AEs	6 to 17	Patients with CD of at least 3 months duration prior to screening, Active CD despite adequate conventional therapy	Induction and maintenance	Industry	Japan	Multi-centre	14	Completed	Yes
			UC	16 Sep 2005<br>*Remicade*	23 Sep 2011<br>*Remicade*	26 Jan 2006<br>*Remicade*	21 Feb 2012<br>*Remicade*														
	12. NCT02624037	Individualized Dosage Precision Infliximab Dashboard<br>*Anti-TNF*	CD	24 Aug 1998<br>*Remicade*	19 May 2006<br>*Remicade*	13 Aug 1999<br>*Remicade*	30 May 2007<br>*Remicade*	Single-arm, open-label	None	Jan 2015	Infliximab trough levels	Anti-infliximab antibody levels	6 – 45	1. IBD diagnosis<br>2. Recently indicated infliximab induction as per standard-of-care	Maintenance	Institutional grant	US	Single-centre	189	Completed	No
			UC	16 Sep 2005<br>*Remicade*	23 Sep 2011<br>*Remicade*	26 Jan 2006<br>*Remicade*	21 Feb 2012<br>*Remicade*														
	13. NCT04004611	Mirikizumab<br>*Anti-IL-23p19*	CD	15 Jan 2025<br>*Omvoh*	*Approval pending*	12 Feb 2025<br>*Omvoh*	*Approval pending*	Parallel, open-label, phase 2	Alternate dosing regimen	May 2020	Clearance of mirikizumab (PK)	1. Clinical remission<br>2. Clinical response<br>3. Endoscopic remission	2 – 17	1. Moderate to severe UC<br>2. Weight>10 kg	Induction and maintenance	Industry	Multi-national	Multi-centre	26	Completed	Yes

Intervention category	Study ID	Intervention	FDA/EMA approval (if applicable)					Study design	Comparator (if applicable)	Initial enrolment	Primary outcome measure(s)	Secondary outcome measure (s)	Age	Population characteristics	Treatment phase	Sponsor	Location	Single- vs multi-centre	Target or actual sample size	Recruitment status	Results (yes/no)
			Indication	FDA – adult	FDA – paediatric	EMA – adult	EMA – paediatric														
			UC	26 Oct 2023<br>*Omvoh*	*Approval pending*	26 May 2023<br>*Omvoh*	*Approval pending*					4. Symptomatic remission<br>5. Height velocity<br>6. Weight<br>7. Patient-reported abdominal pain scores		3. Evidence of UC extending proximal to the rectum<br>4. Inadequate/loss of response/intolerance to immunomodulators, CS, JAK-inhibitors, or biologics							
	14. NCT05784246	Mirikizumab<br>*Anti-IL-23p19*	CD	15 Jan 2025<br>*Omvoh*	*Approval pending*	12 Feb 2025<br>*Omvoh*	*Approval pending*	Parallel, open-label, phase 3	Alternate dosing regimen	Nov 2023	Clinical remission as per mMS at week 52 among the week 12 clinical responders	1. MMS clinical remission<br>2. MMS clinical response<br>3. Endoscopic remission<br>4. Endoscopic improvement<br>5. PUCAI clinical response<br>6. PUCAI clinical remission<br>7. Histologic endoscopic mucosal improvement<br>8. PK	2 – 17	1. Moderate to severe UC<br>2. Weight>10 kg<br>3. Have UC at least 3 months in duration before baseline<br>4. Inadequate/loss of response/intolerance to immunomodulators, CS, JAK-inhibitors, or biologics	Induction and maintenance	Industry	Belgium, Canada, France, Germany, Israel, Italy, Japan, Poland, South Korea, UK, US	Multi-centre	63	Completed	No
			UC	26 Oct 2023<br>*Omvoh*	*Approval pending*	26 May 2023<br>*Omvoh*	*Approval pending*														
	15. NCT02862132	Vedolizumab<br>*Anti-integrin-α4β7*	CD/UC	20 May 2014<br>*Entyvio*	*Approval pending*	22 May 2014<br>*Entyvio*	*Approval pending*	Single-arm, open-label	None	Jan 2017	Complete remission at week 14, 30, 54, 108, 162	1. Clinical remission<br>2. Clinical response<br>3. FCal<br>4. CRP<br>5. Rate of loss of response<br>6. Steroid dependency<br>7. AEs<br>8. Mucosal inflammation<br>9. Time to remission<br>10. lPGA<br>11. Height velocity<br>12. Surgical interventions	0 – 17	1. IBD diagnosis<br>2. Initiating Vedolizumab therapy	Induction and maintenance	Institutional grant	Denmark, Ireland, Israel, Slovenia, UK, US	Multi-centre	142	Completed	No

Intervention category	Study ID	Intervention	FDA/EMA approval (if applicable)					Study design	Comparator (if applicable)	Initial enrolment	Primary outcome measure(s)	Secondary outcome measure (s)	Age	Population characteristics	Treatment phase	Sponsor	Location	Single- vs multi-centre	Target or actual sample size	Recruitment status	Results (yes/no)
			Indication	FDA – adult	FDA – paediatric	EMA – adult	EMA – paediatric														
Small molecule therapy (RCTs)	1. NCT03531892	AJM300<br><br>*Carotegrast methyl*	CD/UC	*Not approved (approved only in Japan for UC)*		*Not approved (approved only in Japan for UC)*		Parallel, quadruple-blind, phase 3	Placebo	Jun 2018	Clinical Response Rate	1. Clinical remission rate<br>2. Clinical response rate<br>3. Mucosal healing rate<br>3. Rectal bleeding subscore<br>4. Change in TMS and pMS<br>5. FCal<br>6. Peripheral white blood cell differentiation	16-74 years	1. Moderate UC diagnosis<br>2. Inadequate response or intolerance to 5-ASA	Induction	Industry	Japan	Multi-centre	198	Completed	No
	2. NCT03945188	Etrasimod<br><br>*Sphingosine-1-phosphate receptor modulator*	CD	Not approved for adult use	Not approved for paediatric use	Not approved for adult use	Not approved for paediatric use	Parallel, double-blind, phase 3	Placebo	Jun 2019	Clinical remission as per mMS at weeks 12 and 52	1. Endoscopic improvement<br>2. Symptomatic remission<br>3. Mucosal healing<br>4. CS-free clinical remission<br>5. Clinical response<br>6. Endoscopic normalization	16 - 80	Active UC diagnosis	Induction	Industry	Multinational	Multi-centre	433	Completed	Yes
			UC	13 Oct 2023<br><br>*Velsipity*	*Approval pending*	16 Feb 2024<br><br>*Velsipity* (16 years or older)	16 Feb 2024<br><br>*Velsipity* (16 years or older)														
	3. NCT03996369	Etrasimod<br><br>*Sphingosine-1-phosphate receptor modulator*	CD	Not approved for adult use	Not approved for paediatric use	Not approved for adult use	Not approved for paediatric use	Parallel, double-blind, phase 3	Placebo	Sep 2020	Clinical remission as per mMS at week 12	1. Endoscopic improvement<br>2. Symptomatic remission<br>3. Mucosal healing<br>4. CS-free clinical remission<br>5. Clinical response<br>6. Endoscopic normalization	16 - 80	Active UC diagnosis	Induction	Industry	Multinational	Multi-centre	354	Completed	Yes
			UC	13 Oct 2023<br><br>*Velsipity*	*Approval pending*	16 Feb 2024<br><br>*Velsipity* (16 years or older)	16 Feb 2024<br><br>*Velsipity* (16 years or older)														
	4. NCT04706793<br><br>*ELEVATE UC JAPAN*	Etrasimod<br><br>*Sphingosine-1-phosphate receptor modulator*	CD	Not approved for adult use	Not approved for paediatric use	Not approved for adult use	Not approved for paediatric use	Parallel, double-blind, phase 3	Placebo	Dec 2020	Clinical remission as per mMS	1. Endoscopic improvement<br>2. Symptomatic remission<br>3. Mucosal healing<br>4. CS-free clinical remission<br>5. Sustained clinical remission	16 - 80	1. Moderate to severe UC<br>2. Completed week 12 of related study APD334 (#64)	Induction	Industry	Japan	Multi-centre	42	Completed	Yes
			UC	13 Oct 2023<br><br>*Velsipity*	*Approval pending*	16 Feb 2024<br><br>*Velsipity* (16 years or older)	16 Feb 2024<br><br>*Velsipity* (16 years or older)														
	5. EudraCT: 2015-000319-41	Ozanimod<br><br>*Sphingosine-1-phosphate receptor modulator*	CD	Not approved for adult use	Not approved for paediatric use	Not approved for adult use	Not approved for paediatric use	Double-blind, phase 3	Placebo	Sep 2015	Induction and maintenance of clinical remission	1. Clinical response<br>2. Endoscopic improvement<br>3. Histologic remission<br>4. Safety and tolerability	12 - 75	1. Active UC diagnosis with complete MS of 6-12<br>2. Endoscopic evidence of extending from the anal verge<br>3. On oral 5-ASAs, immunomodulators, or CS	Induction and maintenance	Industry	Multinational	Multi-centre	1050	Completed	Yes
			UC	27 May 2021<br><br>*Zeposia*	Not approved for paediatric use	18 Nov 2021<br><br>*Zeposia*	Not approved for paediatric use														

Intervention category	Study ID	Intervention	FDA/EMA approval (if applicable)					Study design	Comparator (if applicable)	Initial enrolment	Primary outcome measure(s)	Secondary outcome measure (s)	Age	Population characteristics	Treatment phase	Sponsor	Location	Single- vs multi-centre	Target or actual sample size	Recruitment status	Results (yes/no)
			Indication	FDA – adult	FDA – paediatric	EMA – adult	EMA – paediatric														
	6. NCT02819635	Upadacitinib<br>*JAK Inhibitor*	CD	18 May 2023<br>*Rinvoq*	Not approved for pediatric use	12 Apr 2023<br>*Rinvoq*	Not approved for pediatric use	Parallel, quadruple-blind, phase 2/3	Placebo	Sep 2016	Clinical remission as per mMS for each sub-study	1. Endoscopic improvement<br>2. Clinical remission per MS<br>3. Clinical response per mMS, pMS<br>4. Histologic improvement<br>5. No bowel urgency, abdominal pain<br>6. IBDQ<br>7. FACIT Score	16 - 75	1. Active UC diagnosis for 90 days or more before baseline with mMS of 5-9<br>2. Inadequate response or loss of response or intolerance to oral 5-ASAs, CS, immunosuppressants, and/or biologics	Induction and maintenance	Industry	Multinational	Multi-centre	1302	Completed	Yes
			UC	16 Mar 2022<br>*Rinvoq*	Not approved for pediatric use	22 Jul 2022<br>*Rinvoq*	Not approved for pediatric use														
	7. NCT03653026	Upadacitinib<br>*JAK Inhibitor*	CD	18 May 2023<br>*Rinvoq*	Not approved for pediatric use	12 Apr 2023<br>*Rinvoq*	Not approved for pediatric use	Parallel, quadruple-blind, phase 3	Placebo	Dec 2018	Clinical remission per mMS	1. Endoscopic improvement<br>2. Endoscopic remission<br>3. Clinical response per ADS<br>4. Clinical response per partial ADS<br>5. Histologic-endoscopic mucosal improvement<br>6. No bowel urgency or abdominal pain<br>7. Histologic improvement<br>8. IBDQ<br>9. Mucosal healing<br>10. FACIT-F Score	16 - 75	1. Active UC with mMS of 5-9<br>2. Inadequate response, loss of response, or intolerance to 5-ASAa, CS, immunosuppressants, and/or biologic therapies.	Induction	Industry	Multinational	Multi-centre	522	Completed	Yes
			UC	16 Mar 2022<br>*Rinvoq*	Not approved for pediatric use	22 Jul 2022<br>*Rinvoq*	Not approved for pediatric use														
	8. NCT05507203	Obefazimod	CD/UC	Not approved for adult use	Not approved for paediatric use	Not approved for adult use	Not approved for paediatric use	Parallel, triple-blind, phase 3	Placebo	Oct 2022	Clinical remission (mMS)	1. Endoscopic improvement<br>2. Clinical response<br>3. Symptomatic remission<br>4. Histologic-endoscopic mucosal	16+	Moderate-severe active UC	Induction	Industry	Multinational	Multi-centre	639	Completed	No
	9. NCT05507216	Obefazimod	CD/UC	Not approved for adult use	Not approved for paediatric use	Not approved for adult use	Not approved for paediatric use	Parallel, triple-blind, phase 3	Placebo	Dec 2022	Clinical remission (mMS)	1. Endoscopic improvement<br>2. Clinical response<br>3. Symptomatic remission<br>4. Histologic-endoscopic mucosal	16+	Moderate-severe active UC	Induction	Industry	Multinational	Multi-centre	636	Completed	No
5-ASA or mesalamine (RCTs)	1. NCT00713310	Mesalamine<br>(*Asacol*)	UC	31 Jan 1992<br>(discontinued in April 2013)	18 Oct 2013<br>(discontinued in April 2013)	Authorized in various countries in parallel via DCP (decentralized procedure; do not need to be authorized by the centralized procedure)		Parallel, triple-blind, phase 3	High dose (2g – 4.8g/day) vs low dose (1.2g – 2.4g/day)	Dec 2008	Treatment success (PUCAI<10 or reduction of ≥20 points from baseline to week 6 with week 6 score ≥ 10 (partial)	NA	5 - 17	Mild to moderate UC with PUCAI between 10 and 55 and does not require steroids	Induction	Industry	Canada, Croatia, Poland, Romania, US	Multi-centre	83	Completed	Yes

Intervention category	Study ID	Intervention	FDA/EMA approval (if applicable)					Study design	Comparator (if applicable)	Initial enrolment	Primary outcome measure(s)	Secondary outcome measure (s)	Age	Population characteristics	Treatment phase	Sponsor	Location	Single- vs multi-centre	Target or actual sample size	Recruitment status	Results (yes/no)
			Indication	FDA – adult	FDA – paediatric	EMA – adult	EMA – paediatric														
	2.<br>NCT01201122<br><br>*The MUPPIT Trial*	Mesalamine<br><br>*(Pentasa)*	UC	10 Sep 1999<br><br>*Pentasa (tablet formulation)*	Not approved for pediatric use	Authorized in various countries in parallel via DCP (decentralized procedure; do not need to be authorized by the centralized procedure)		Parallel, single-blind, phase 4	Alternate dosing regimen – once daily vs twice daily	Sep 2010	Difference in mean PUCAI score between groups	Complete response or large partial response as per PUCAI	6 - 18	1. Mild to moderate UC with PUCAI 10 – 55 points<br>2. Weight at least 15 kg	Induction	Institutional grant	Israel	Multi-centre	86	Completed	No
	3.<br>NCT01130844	MMX Mesalamine<br><br>*(Lialda)*	UC	16 Jan 2007<br><br>*Lialda*	26 Jun 2020<br><br>*Lialda*	Specific brand not approved; mesalamine, mesalazine and associated products marketed and authorized via DCP licensing		Parallel, open-label, phase 1	Alternate dosing regimen for MMX Mesalamine	Oct 2010	1. AUC<br>2. Cmax<br>3. Tmax<br>4. Total body clearance (CL)<br>5. AUC, Cmax, Tmax, and CL of Ac-5-ASA	1. Dose absorbed in urine<br>2. Amount recovered in urine<br>3. Ac-5-ASA recovered in urine	5 - 17	1. UC diagnosis for at least 3 months stable on or off 5-ASA containing/metabolizing treatments<br>2. Weight of 18-82 kg	NA	Institutional grant	Australia, Poland, Slovakia, UK, US	Multi-centre	52	Completed	Yes
	4.<br>NCT02093663	MMX Mesalamine<br><br>*(Lialda)*	UC	16 Jan 2007<br><br>*Lialda*	26 Jun 2020<br><br>*Lialda*	Specific brand not approved; mesalamine, mesalazine and associated products marketed and authorized via DCP licensing		Parallel, quadruple-blind, phase 3	Alternate dosing regimen – high versus low dose	Dec 2014	1. Acute phase clinical response (week 8)<br>2. Maintenance phase clinical response (week 26)	1. Acute phase and maintenance phase clinical and endoscopic clinical response (week 8, week 26) – central and local<br>2. Change in DUCS (week 8, week 26)<br>3. PUCAI remission (week 26)	5 - 17	1. Weight 18-90 kg<br>2. Mild to moderate UC<br>3. Acute phase: Partial UCDAI score>2 at baseline with stable 5-ASA as part of normal treatment<br>4. Maintenance phase: Partial UCDAI score>1	Induction and maintenance	Industry	Canada, Hungary, Israel, Poland, Slovakia, UK, US	Multi-centre	107	Completed	Yes
	5.<br>NCT00225810	Mesalamine<br><br>*(Pentasa sachets, 1 g)*	UC	10 Sep 1999<br><br>*Pentasa (tablet formulation)*	Not approved for paediatric use	Authorized in various countries in parallel via DCP (decentralized procedure; do not need to be authorized by the centralized procedure)		Cross-over, open-label, phase 4	Pentasa tablets (500 mg)	Oct 2005	Acceptability	1. AEs<br>2. Stool and urine<br>3. Concentration of mesalazine<br>4. PCDAI	8 - 18	1. CD diagnosis<br>2. Weight < 40 kg<br>3. Oral maintenance treatment with 5-ASA and any anti-inflammatory medication	Maintenance	Industry	Czech Republic	Multi-centre	50	Completed	No
5-ASA or mesalamine (non-RCTs)	1.<br>NCT01536535	Mesalamine<br><br>*(Pentasa)*	UC	10 Sep 1999<br><br>*Pentasa (tablet formulation)*	Not approved for paediatric use	Authorized in various countries in parallel via DCP (decentralized procedure; do not need to be authorized by the centralized procedure)		Parallel, open-label, phase 4	Prednisone – oral and IV, anti-TNFα, calcineurin inhibitors, immunomodulators, colectomy	Jul 2012	1. CS-free remission as per PUCAI<br>2. Need for additional therapy or colectomy	Number of participants receiving a colectomy	4 to 17	New diagnosis of UC by standard clinical, endoscopic, and histologic features; colitis extending beyond the rectosigmoid; PUCAI of ≥10 at diagnosis; no prior therapy	Induction and maintenance	Institutional grant	Canada, US	Multi-centre	431	Completed	Yes
Corticosteroids and immunosuppressants (RCTs)	1.<br>NCT03412682	Budesonide<br><br>*Glucocortico-steroid*	CD<br><br>UC	02 Oct 2001<br><br>*Entocort*<br><br>14 Jan 2013<br><br>*Uceris (tablets)*	29 Apr 2016<br><br>*Entocort*<br><br>Not approved for paediatric use	Not approved for IBD indications<br>Entocort may be licensed via "historical" national license		Parallel, double-blind, phase 3	Mesalazine	Mar 2018	Change in UCDAI	1. Clinical remission<br>2. UCDAI<br>3. Clinical improvement<br>4. Endoscopic improvement<br>5. Symptom resolution<br>6. Change in partial UCDAI	16 - 75	Mild- to moderate active UC	Induction	Industry	Japan	Multi-centre	274	Completed	No
	2.<br>NCT01514240	Budesonide (D9421-C)	CD	02 Oct 2001<br><br>*Entocort*	29 Apr 2016<br><br>*Entocort*	Not approved for IBD indications		Parallel, triple-blind, phase 3	Mesalazine	Feb 2012	Remission at week 8	1. CDAI remission<br>2. Change in CDAI	15+	1. Mild to moderate active CD (CDAI 180 – 400)	Induction	Industry	Japan	Multi-centre	123	Completed	Yes

Intervention category	Study ID	Intervention	FDA/EMA approval (if applicable)					Study design	Comparator (if applicable)	Initial enrolment	Primary outcome measure(s)	Secondary outcome measure (s)	Age	Population characteristics	Treatment phase	Sponsor	Location	Single- vs multi-centre	Target or actual sample size	Recruitment status	Results (yes/no)
			Indication	FDA – adult	FDA – paediatric	EMA – adult	EMA – paediatric														
		Glucocortico-steroid	UC	14 Jan 2013<br>*Uceris (tablets)*	Not approved for paediatric use	Entocort may be licensed via "historical" national license						3. Cumulative remission rate<br>4. Clinical improvement rates<br>5. Change in IBDQ scores		2.Ileal/ileocecal and/or ascending colonic disase							
	3. NCT02772965	Methotrexate<br>*Antimetabolite*	CD	Not approved for adult use in IBD – off-label use	Not approved for paediatric use in IBD – off-label use	22 Aug 2019<br>IV form combined with corticosteroid approved with limited dosing for adult CD	Not approved for pediatric use	Parallel, triple-blind, phase 3	Placebo	Oct 2016	Treatment failure	1. Mean Patient Reported Outcome Measurement and Information System score<br>2. Positive anti-TNF antibody	0 - 20	CD patients initiating anti-TNF therapy with infliximab or adalimumab	Induction and maintenance	Government agency	US	Multi-centre	306	Completed	Yes
	4. NCT00347048	Tacrolimus<br>*Calcineurin Inhibitor*	Non-IBD indications	Approved for non-IBD indications		Approved for non-IBD indications		Parallel, quadruple-blind, phase 3	Placebo	Sep 2006	Improvement in DAI score at week 2	1. Change in DAI (total and each item) at week 2<br>2. Change in clinical severity and symptoms<br>3. Endoscopic finding<br>4. Patient's impression<br>5. Amount of steroid	16 - 64	Moderate to severe refractory UC with steroid resistance or dependence	Induction and maintenance	Industry	Japan	Multi-centre	62	Completed	No
	5. NCT00720538	Thalidomide	Non-IBD indications	Approved for non-IBD indications		Approved for non-IBD indications		Parallel, quadruple-blind, phase 3	Placebo	Aug 2008	Clinical remission at 8, 12, 26 and 52 weeks	1. Clinical response<br>2. Steroid dose reduction<br>3. Endoscopic remission<br>4. AEs<br>5. Weight, BMI and height	2 - 20	Chronic refractory moderate to severe IBD	Induction	Institutional grant	Italy	Multi-centre	84	Completed	Yes
Corticosteroids and immunosuppressants (non-RCTs)	1. NCT01444092	Budesonide<br>*Glucocortico-steroid*	CD<br>UC	02 Oct 2001 *Entocort*<br>14 Jan 2013 *Uceris (tablets)*	29 Apr 2016 *Entocort*<br>Not approved for paediatric use	Not approved for IBD indications<br>Entocort may be licensed via "historical" national license		Single-arm, open-label, phase 3	None	Nov 2011	Adverse event(s)	1. PCDAI<br>2. IMPACT III	5 to 17	Active CD of the ileum and/or ascending colon confirmed by endoscopic and/or radiographic evidence, and/or evidence of mucosal erosions and/or histology	Induction and maintenance	Industry	Canada, Germany, Italy, Poland, US	Multi-centre	123	Completed	Yes
	2. NCT01453946	Budesonide<br>*Glucocortico-steroid*	CD<br>UC	02 Oct 2001 *Entocort*<br>14 Jan 2013 *Uceris (tablets)*	29 Apr 2016 *Entocort*<br>Not approved for paediatric use	Not approved for IBD indications<br>Entocort may be licensed via "historical" national license		Single-arm, open-label, phase 3	None	Dec 2011	Adverse event(s)	1. PCDAI<br>2. IMPACT III	5 to 17	Active CD of the ileum and/or ascending colon confirmed by endoscopic and/or radiographic evidence, and/or evidence of mucosal erosions and/or histology and a PCDAI≤10	Maintenance	Industry	Canada, Germany, Italy, Poland, US	Multi-centre	55	Completed	Yes
	3. NCT02269358	Methotrexate<br>*Antimetabolite*	CD/UC	Not approved for adult use in	Not approved for paediatric	22 Aug 2019<br>IV form combined	Not approved for pediatric use	Single-arm, open-label, phase 4	None	Jul 2015	Remission	1. Remission<br>2. Trough levels<br>3. ATIs	8 to 18	Established diagnosis of CD;<br>Active disease PCDAI >10 at least two weeks	Induction and maintenance	individual	Israel	Single-centre	22	Completed	No

Intervention category	Study ID	Intervention	FDA/EMA approval (if applicable)					Study design	Comparator (if applicable)	Initial enrolment	Primary outcome measure(s)	Secondary outcome measure (s)	Age	Population characteristics	Treatment phase	Sponsor	Location	Single- vs multi-centre	Target or actual sample size	Recruitment status	Results (yes/no)
			Indication	FDA – adult	FDA – paediatric	EMA – adult	EMA – paediatric														
				IBD – off-label use	use in IBD – off-label use	with corticosteroid approved with limited dosing for adult CD								after infusion;<br>On Infliximab with at least 3 prior infusions, and still active despite a decreased dose interval (≤6 weeks) or increased dose of infliximab (≥7.5 mg/kg /dose Q8W)							
	4. NCT00643071	Tacrolimus<br><br>*Calcineurin Inhibitor*	Non-IBD indications	Approved for non-IBD indications		Approved for non-IBD indications		Single-arm, open-label, phase 3	None	Sep 2006	Improvement in DAI score	1. Change in DAI<br>2. Changes in clinical severity and symptoms<br>3. Endoscopic findings<br>4. Patients' impression<br>5. Amount of steroid	16 to 64	Severe refractory UC patients<br>or<br>Moderate to severe refractory UC patients who participated and received placebo in F506-CL-1107 study	NA	Industry	Japan	Multi-centre	32	Completed	No
	5. NCT01233570	Tacrolimus<br><br>*Calcineurin Inhibitor*	Non-IBD indications	Approved for non-IBD indications		Approved for non-IBD indications		Single-arm, open-label, phase 2	None	Unavailable	Standardized digital photography following Physicians' Global Severity Scale pre- and prost-treatment	1. Global self-assessment<br>2. Perianal disease activity index	12+	Confirmed diagnosis of CD of at least 3 months duration confirmed by radiography, endoscopy or pathological examination,<br>required to have a skin manifestation, long-standing, concomitant IS therapy was allowed if the dose was stable and not controlling the skin problem	NA	Institutional grant	UK	Single-centre	20	Completed	No
	6. NCT05587673	Methylprednisolone<br><br>*Corticosteroid*	CD/UC	24 Oct 1957	Not approved for pediatric use	Nationally authorized	Not approved for pediatric use	Single-arm, open-label, phase 1	None	Oct 2022	Number of patients with symptomatic, imaging or biopsy related improvement in their IBD symptoms	1. Number of patients with pre-procedural and procedural imaging correlated with clinical outcomes<br>2. Number of patients with molecular changes correlated with improvement in symptoms	4-25	1. Newly or established IBD diagnosis with acute flare not being controlled with first line therapy<br>2. Present with typical IBD symptoms (eg, abdominal pain, loss of appetite, rectal bleeding, diarrhoea, obstruction, or passage of mucus)	NA	Institutional grant	US	Single-centre	3	Completed	No
Nutritional and dietary therapy (RCTs)	1. NCT05034458	CDED	CD	Not approved; dietary supplements do not need FDA approval		Not approved		Parallel, open-label	None	May 2021	FCal	Adherence	1 month-17 years	CD patients with complete remission induction on biologic therapy<br>FCal higher than lab standards (250 ug.g)	Induction	Institutional grant	Argentina	Single-centre	30	Completed	No
	2. NCT02843100	EEN	CD/UC	Not approved; dietary supplements do not need FDA approval		Not approved		Parallel, open-label	CDED<br>PEN	Apr 2018	Clinical remission as per PCDAI	1. Remission<br>2. Microbiome<br>3. FCal<br>4. Additional treatment<br>5. Mucosal healing	8 - 18	1. Mild to severe CD with PCDAI 15 – 47.5 incl.<br>2. Active inflammation (CRP>0.6 mg/dL or	Induction and maintenance	Industry	Canada, Ireland, Israel, Spain	Multi-centre	63	Completed	No

Intervention category	Study ID	Intervention	FDA/EMA approval (if applicable)					Study design	Comparator (if applicable)	Initial enrolment	Primary outcome measure(s)	Secondary outcome measure (s)	Age	Population characteristics	Treatment phase	Sponsor	Location	Single- vs multi-centre	Target or actual sample size	Recruitment status	Results (yes/no)
			Indication	FDA – adult	FDA – paediatric	EMA – adult	EMA – paediatric														
														ESR>20 or Calprotectin>200 μg/g)							
	3. NCT00838149	Glutamine	CD/UC	Not approved; dietary supplements do not need FDA approval		Not approved		Parallel, open-label, phase 1	Whey protein	Nov 2005	Improvement in intestinal permeability at 2 months	Change in small intestinal morphology	15 - 60	CD patients in remission with an abnormal intestinal permeability	NA	Institutional grant	India	Single-centre	30	Completed	No
	4. NCT00940576	Mare's milk	CD/UC	Not approved; dietary supplements do not need FDA approval		Not approved		Cross-over, quadruple-blind	Placebo	Jul 2000	CDAI – CD<br>CAI – UC	Patient-reported extraintestinal disorders	10 - 50	Clear and definite CD or UC diagnosis	NA	Institutional grant	Germany	Multi-centre	17	Completed	Yes
	5. NCT02201693	Cyclic EEN (Modulen IBD)	CD/UC	Not approved; dietary supplements do not need FDA approval		Not approved		Parallel, open-label	Modulen IBD – low dose (25% of daily caloric requirements)	Dec 2014	First relapse as per wPCDAI	1. Clinical remission as per wPCDAI CDAI, PGA<br>2. Time to first relapse<br>3. Variation of wPCDAI<br>4. Endoscopic remission via CDEIS<br>5. Transmural healing<br>6. FCal<br>7. QoL questionnaire<br>8. Growth pattern – Z-score	6 - 18	1. New-onset or acute relapse of CD treated with enteral nutrition<br>2. Stopping all other therapies (immunomodulators, antibiotics, anti-TNF, etc.) prior to study start	Induction and maintenance	Institutional grant	France	Multi-centre	100	Completed	No
	6. NCT03653481	Oligofructose-enriched Inulin<br><br>*Dietary fiber Therapy*	CD/UC	Not approved; dietary supplements do not need FDA approval		Not approved		Parallel, quadruple-blind	Maltodextrin (placebo)	Oct 2018	Alteration of gut microbiota and metabolome	Change in FCal	8 - 21	1. Colonic/ileocolonic CD, UC, or IBD-U<br>2. PCDAI<15 incl. or PGA quiescent<br>3. No visible blood in bowel movements 7 days prior<br>4. Normal inflammatory lab markers	Induction	Industry	US	Single-centre	51	Completed	No
	7. NCT02610101	Specific carbohydrate diet	CD/UC	Not subject to FDA approval (diet therapy)		Not subject to EMA approval (diet therapy)		Triple-blind, factorial	Modified specific carbohydrate diet; whole foods diet	Nov 2015	Change in PCDAI	1. Faecal microbiome<br>2. CRP<br>3. FCal	12 - 21	Mild to moderate CD diagnosis (PCDAI 10 – 45) on stable medication for at least 1 month before start	NA	Institutional grant	US	Single-centre	18	Completed	No
	8. NCT03301311	Specific carbohydrate diet	CD/UC	Not subject to FDA approval (diet therapy)		Not subject to EMA approval (diet therapy)		Cross-over, open-label	Modified specific carbohydrate diet	Apr 2018	1. Stool frequency & consistency<br>2. Pain interference<br>3. Gastrointestinal symptoms<br>4. FCal	1. PUCAI/Spcdai<br>2. CRP, ESR, Albumin, HCT<br>3. Growth<br>4. sCDAI<br>5. PUCAI	7 - 18	UC or IBD-U diagnosis with evidence of acute inflammation and/or elevated acute phase reactant	NA	Institutional grant	US	Multi-centre	54	Completed	No
	9. NCT05456763	Sodium butyrate	CD/UC	Not approved; dietary supplements do not need FDA approval		Not approved (not listed in Directive 2002/46/EC on the approximation of laws relating to food supplements)		Parallel, triple-blind	Placebo	Jun 2013	1. Remission rate as per PCDAI<br>2. Remission rate as per PUCAI	1. PCDAI/PUCAI<br>2. FCal<br>3. Side effects	6 - 18	IBD diagnosis with colon involvement	Induction	Institutional grant	NA	Multi-centre	100	Completed	No

Intervention category	Study ID	Intervention	FDA/EMA approval (if applicable)					Study design	Comparator (if applicable)	Initial enrolment	Primary outcome measure(s)	Secondary outcome measure (s)	Age	Population characteristics	Treatment phase	Sponsor	Location	Single- vs multi-centre	Target or actual sample size	Recruitment status	Results (yes/no)
			Indication	FDA – adult	FDA – paediatric	EMA – adult	EMA – paediatric														
	10. NCT01728870	PEN + whole food diet	CD/UC	Not approved; dietary supplements do not need FDA approval		Not approved		Parallel, open-label	EEN + free diet	Jan 2013	1. Patient adherence<br>2. MARS questionnaire	1. PCDAI response<br>2. PCDAI remission<br>3. Bone health<br>4. CRP<br>5. Physician assessment of compliance	4 - 18	1. CD diagnosis with macroscopic small bowel involvement or isolated large bowel disease confined to the right or transverse colon<br>2. PCDAI>10	Induction	Individual	Canada, Israel	Multi-centre	74	Completed	Yes
	11. NCT01187459	Vitamin D	CD/UC	Present in ingredient directory for dietary supplements (in combination with calcium)		Present in the Directive 2002/46/EC on the approximation of laws relating to food supplements)		Parallel, double-blind, phase 4	Alternate dose	Sep 2010	Number of patients with optimal 25OHD concentration	PCDAI to determine likelihood of remaining in remission	8 - 18	CD diagnosis with remission for at least 4 weeks as per PCDAI < 10	Maintenance	Institutional grant	Canada	Multi-centre	87	Completed	Yes
	12. NCT01692808	Vitamin D3 + EEN/CS	CD/UC	Vitamin D present in ingredient directory for dietary supplements (in combination with calcium)		Vitamin D present in the Directive 2002/46/EC on the approximation of laws relating to food supplements)		Parallel, open-label, phase 2	EEN only<br>CS only	Oct 2012	Number of AEs as per 25OHD blood levels, calcium, phosphorus, PTH	1. Decrease in inflammatory parameters<br>2. Immunological changes<br>3. Bioavailability	10 - 18	CD diagnosis with recent blood test (albumin, ESR, HCT)	NA	Institutional grant	Canada	Single-centre	20	Completed	No
	13. NCT02186275	Vitamin D3 (high dose)	CD/UC	Present in ingredient directory for dietary supplements (in combination with calcium)		Present in the Directive 2002/46/EC on the approximation of laws relating to food supplements)		Parallel, triple-blind, phase 3	Vitamin D3 – low dose	Feb 2016	Occurrence of relapse as per PCDAI	1. Time to first relapse<br>2. Relapses per year per patient<br>3. Duration of CS<br>4. CD-related hospitalizations<br>5. IMPACT III<br>6. Canadian Health Measures Survey – Children's Physical Activity Questionnaire<br>7. Change in bone mineral density	9 - 18	CD diagnosis with PCDAI < 30 incl. and concurrent treatment with CS and/or enteral nutrition and/or thiopurines and/or MTX and/or 5-ASAs and/or anti-TNF	Induction and maintenance	Institutional grant	Canada	Multi-centre	25	Completed	No
	14. NCT01957423	Whey protein	CD/UC	Not approved; dietary supplements do not need FDA approval		Not approved		Parallel, double-blind	Soy protein	Mar 2012	BMI	1. Body fat percentage<br>2. Body lean percentage<br>3. Mid-arm circumference<br>4. Mid-arm muscle circumference<br>5. Corrected arm muscle area<br>6. CDAI<br>7. CRP<br>8. Serum albumin<br>9. Pre-albumin<br>10. Triceps skin fold thickness	0 to 65+	CD patients treated with AZA or anti-TNF or both	NA	Institutional grant	Brazil	Single-centre	41	Completed	No
	15. NCT04239248	Whole food (Tasty&Healthy) diet	CD	Not subject to FDA approval (diet therapy)		Not subject to EMA approval (diet therapy)		Parallel, single-blind	EEN, no intervention	Apr 2020	Tolerability	1. Mucosal healing (Mucosal Inflammation Noninvasive Index)<br>2. Clinical remission<br>3. FCal	6-40	1. Mild to moderate CD with <1 years of disease since diagnosis<br>2. No current/prior treatment with 5-ASA,	Induction and maintenance	Institutional grant	Israel	Single-centre	129	Completed	Yes (induction results)

Intervention category	Study ID	Intervention	FDA/EMA approval (if applicable)					Study design	Comparator (if applicable)	Initial enrolment	Primary outcome measure(s)	Secondary outcome measure (s)	Age	Population characteristics	Treatment phase	Sponsor	Location	Single- vs multi-centre	Target or actual sample size	Recruitment status	Results (yes/no)
			Indication	FDA – adult	FDA – paediatric	EMA – adult	EMA – paediatric														
														immunomodulators or biologics							
Nutritional and dietary therapy (non-RCTs)	1. NCT02472457	CDED	CD	Not approved; dietary supplements do not need FDA approval		Not approved		Parallel, open-label	Placebo – free diet	Aug 2015	Remission as per PCDAI, CRP, FCal	1. Time to relapse<br>2. Microbial composition<br><br>Other outcomes: Dietary compliance	10 to 21	CD patients withdrawing from biologic or IMs monotherapy as part of clinical care;<br>Normal Growth Velocity, or Tanner 5<br>Steroid-free Remission (PCDAI <10 without the height component) for at least 12 months prior to enrollment<br>Colonoscopy during the preceding 3 months with complete mucosal healing or only few aphthous ulcerations;<br>Stool calprotectin <250µg/g during the preceding 3 months	Induction and maintenance	Institutional grant	US	Single-centre	19	Completed	Yes
	2. NCT00889161	Curcumin	CD/UC	Not approved; dietary supplements do not need FDA approval		Not approved		Single-arm, open-label, phase 1	None	May 2009	Tolerability	None provided	8 to 18	Children and adolescents 8-18 years old with pIBD	NA	Institutional grant	US	Single-centre	11	Completed	Yes
	3. NCT03755583	EEN	CD/UC	Not approved; dietary supplements do not need FDA approval		Not approved		Single-arm, open-label	None	Feb 2017	1. 5-item gut microbiota measure<br>2. 2-item SES-CD	1. 5-item wPCDAI<br>2. 2-item 25-OH VITD3<br>3. Weight<br>4. Height<br>5. FCal<br>6. IGF-1<br>7. Calcium	1 to 17	Pediatric CD patients who were newly diagnosed and being treated with EEN	Induction	Institutional grant	China	Single-centre	31	Completed	No
	4. NCT04757181	IBD-AID	CD/UC	Not subject to FDA approval (diet therapy)		Not subject to EMA approval (diet therapy)		Single-arm, open-label	None	Feb 2017	Microbiome change	1. Improvement of symptoms<br>2. Improvement of inflammatory markers	15+	Diagnosis of IBD, specifically CD and UC	NA	Institutional grant	US	Single-centre	38	Completed	No
	5. NCT03644602	Low FODMAP Diet	CD/UC	Not subject to FDA approval (diet therapy)		Not subject to EMA approval (diet therapy)		Single-arm, open-label	None	Jul 2016	IBS-SSS questionnaire	SF-36 questionnaire	0 to 65+	IBD patients in remission	NA	Institutional grant	Italy	Single-centre	150	Completed	No
	6. NCT02213835	Specific carbohydrate diet	CD/UC	Not subject to FDA approval (diet therapy)		Not subject to EMA approval (diet therapy)		Single-arm, open-label	None	Jun 2014	PCDAI/PUCAI	Change in lab measures – CBC, sedimentation rate, CRP, FCal<br><br>Other outcomes: Stool microbiome analysis	8 to 21	Diagnosis of UC or CD based on history, physical exam, laboratory, radiological studies and histology;<br>Mild or moderate disease activity based upon PCDAI score (10-45) or PUCAI score (10-60)	NA	Individual	US	Single-centre	9	Completed	Yes
	7. NCT02922881	Ulcerative Colitis Diet	CD/UC	Not subject to FDA approval (diet therapy)		Not subject to EMA approval (diet therapy)		Single-arm, open-label	None	Aug 2015	Clinical remission defined by PUCAI	1. Dietary compliance – MARS questionnaire<br>2. CRP	8 to 19	1. Established UC diagnosis<br>2. Mild to moderate active disease,	Induction	Governmental agency	US	Single-centre	5	Completed	No

Intervention category	Study ID	Intervention	FDA/EMA approval (if applicable)					Study design	Comparator (if applicable)	Initial enrolment	Primary outcome measure(s)	Secondary outcome measure (s)	Age	Population characteristics	Treatment phase	Sponsor	Location	Single- vs multi-centre	Target or actual sample size	Recruitment status	Results (yes/no)
			Indication	FDA – adult	FDA – paediatric	EMA – adult	EMA – paediatric														
												3. ESR<br>4. FCal<br>5. Microbial composition		10<PUCAI<45<br>3. Stable medication (IMM/5-ASA) use or no medication use for the past 6 weeks							
	8. NCT02345733	Ulcerative Colitis Diet	CD/UC	Not subject to FDA approval (diet therapy)		Not subject to EMA approval (diet therapy)		Parallel, open-label, phase 4	Antibiotic treatment	Sep 2015	Remission rate as per PUCAI	1. Mean PUCAI<br>2. Mean FCal<br>3. PGA<br>4. Remission as per PUCAI	8 to 19	Established diagnosis of UC;<br>Mild to moderate active disease, PUCAI ≤10 and ≤45;<br>Stable medication (IMM/ 5ASA) use for the past 6 weeks	Induction	individual	Canada, Israel, US	Multi-centre	20	Completed	No
	9. NCT03508193	Whole-food smoothie	CD/UC	Not subject to FDA approval (diet therapy)		Not subject to EMA approval (diet therapy)		Single-arm, open-label	None	Nov 2017	FCal	IMPACT III	8 to 21	1. CD diagnosis within 4 weeks of study entry<br>2. Active CD (PCDAI ≥10)	Induction	Institutional grant	US	Single-centre	12	Completed	Yes
	10. NCT04782544	Buttermilk powder	CD/UC	Not approved; dietary supplements do not need FDA approval		Not approved		Parallel, double-blind	Placebo (anhydrous milk fat)	June 2021	PUCAI or PCDAI	1. ImproveCareNow Physician Global Assessment<br>2. IMPACT-III 3. intestinal permeability (lactulose/mannitol urinary assay)<br>4. CRP<br>5. FCal	9-21	1. Mild-moderate or severe IBD defined by ImproveCareNow Physician Global Assessment<br>2. Ileal, ileocolonic, colonic location of disease	Induction	Institutional grant	US	Single-centre	11	Completed	No
Microbiological and microbiota interventions (RCTs)	1. NCT02330653	FMT	CD/UC	Not approved; regulation and support for development provided<br><br>*Rebyota (rectal formulation) approved on Nov 30, 2022		Not approved; regulatory submissions ongoing for marketing authorization in 5-10 years		Parallel, quadruple-blind, phase 1/2	Placebo	Nov 2015	FMT-related AEs	1. Remission as per PUCAI or PCDAI<br>2. Improvement in FCal, ESR, CRP, Albumin, HCT<br>3. Percent donor microbiome<br>4. Improvement as per PUCAI or PCDAI	5 - 30	1. UC (PUCAI>9) or CD (PCDAI>10) diagnosis with intolerance, failure, or refusal to first-line maintenance<br>2. Visual or histologic evidence of inflammation<br>3. Negative tests for HBV, HCV, HIV	Induction and maintenance	Individual	US	Single-centre	15	Completed	Yes
	2. NCT02487238	FMT - Enema	CD/UC	Not approved; regulation and support for development provided<br><br>*Rebyota (rectal formulation) approved on Nov 30, 2022		Not approved; regulatory submissions ongoing for marketing authorization in 5-10 years		Parallel, quadruple-blind, phase 1	Placebo – Saline Enema	Nov 2015	Feasibility – via recruitment, retention, eligibility, acceptance, AEs	1. Microbiome change<br>2. Remission as per PUCAI<br>3. Clinical improvement as per PUCAI<br>4. Biological improvement as per ESR, CRP, Hb/Albumin<br>5. Mucosal healing<br>6. Urine metabolomics change	3 - 17	1. UC or IBD-U diagnosis with evidence of active disease<br>2. Any ongoing treatment must be stable	Induction and maintenance	Institutional grant	Canada	Multi-centre	35	Completed	Yes
	3. NCT02704728	*Bacteroides thetaiotaomicron* (Thetanix)	CD/UC	Not approved		Not approved		Parallel, double-blind	Placebo	Dec 2015	TEAEs	1. PGA<br>2. wPCDAI	16 – 18	Confirmed CD diagnosis currently in clinical remission	NA	Industry	UK	Multi-centre	18	Completed	No

Intervention category	Study ID	Intervention	FDA/EMA approval (if applicable)					Study design	Comparator (if applicable)	Initial enrolment	Primary outcome measure(s)	Secondary outcome measure (s)	Age	Population characteristics	Treatment phase	Sponsor	Location	Single- vs multi-centre	Target or actual sample size	Recruitment status	Results (yes/no)
			Indication	FDA – adult	FDA – paediatric	EMA – adult	EMA – paediatric														
		Probiotic supplement										3. Clinical and non-clinical lab tests<br>4. Faecal microbiome<br>5. FCal									
	4.<br>NCT00175292	VSL#3<br><br>*Probiotic supplement*	Unknown indications	Limited approval issued; indications unknown<br><br>(Currently used for UC, ileal pouch, and IBS)		Not approved		Parallel, double-blind, phase 3	Placebo	Dec 2003	Endoscopic recurrence	Endoscopic recurrence at 90 and 360 days<br>CDAI<br>QoL<br>Adverse events	16+	CD diagnosis with resection of ileocolonic disease and small bowel to colonic anastomosis within 30 days of randomization	Maintenance	Government agency	Canada	Multi-centre	120	Completed	No
	5.<br>NCT00944736	VSL#3<br><br>*Probiotic supplement*	Unknown indications	Limited approval issued; indications unknown<br><br>(Currently used for UC, ileal pouch, and IBS)		Not approved		Parallel, triple-blind, phase 3	Placebo	Jul 2009	Intestinal permeability via Sugar Absorption Test	None provided	11 - 17	1. Small bowel or colonic CD in remission as per PCDAI<10<br>2. On maintenance 5-ASA, 6-MP, and/or AZA for at least 2 months	NA	Institutional grant	US	Single-centre	12	Completed	Yes
	6.<br>NCT03378167	FMT	CD	Not approved; regulation and support for development provided<br><br>*Rebyota (rectal formulation) approved on Nov 30, 2022		Not approved; regulatory submissions ongoing for marketing authorization in 5-10 years		Parallel, triple-blind, phase 1	Placebo	Dec 2018	Recruitment rate, drop out rate, patient protocol adherence, AEs	1. Improvement as per PCDAI<br>2. Decrease in CRP<br>3. Change in urine metabolite profile<br>4. Change in faecal 16s rRNA + metagenomics profile	3 – 17	1. CD or IBD-U favoring CD<br>2. Active disease	NA	Institutional grant	Canada	Multi-centre	17	Completed	No
Microbiological and microbiota interventions (non-RCTs)	1.<br>NCT01560819	FMT	CD/UC	Not approved; regulation and support for development provided<br><br>*Rebyota (rectal formulation) approved on Nov 30, 2022		Not approved; regulatory submissions ongoing for marketing authorization in 5-10 years		Single-arm, open-label, phase 1	None	Mar 2012	Clinical response as per PUCAI	None provided	7 to 21	Established diagnosis of pIBD; mild to moderate disease: PUCAI of 10-65; stable disease activity and therapy for two months prior to FMT procedure	Induction and maintenance	Institutional grant	US	Single-centre	20	Completed	Yes
	2.<br>NCT01757964	FMT	CD/UC	Not approved; regulation and support for development provided<br><br>*Rebyota (rectal formulation) approved on Nov 30, 2022		Not approved; regulatory submissions ongoing for marketing authorization in 5-10 years		Single-arm, open-label, phase 1	None	Dec 2012	Clinical response as per PCDAI or PUCAI	None provided	12 to 21	Patients with mild to moderate UC or CD<br>Parent willing to donate stool for the transplant	Induction	individual	US	Single-centre	13	Completed	Yes
	3.<br>NCT03268213	FMT	CD/UC	Not approved; regulation and support for development provided<br><br>*Rebyota (rectal formulation) approved on Nov 30, 2022		Not approved; regulatory submissions ongoing for marketing authorization in 5-10 years		Single-arm, open-label, early phase 1	None	Nov 2013	TEAEs	1. Microbial diversity<br>2. Microbial composition<br>3. Microbial function<br>4. FCal<br>5. Recurrent *C. difficile*	7+	UC patients with one of the following:<br>Treated with steroids, IMs, or biologic therapy for at least one month	NA	Institutional grant	US	Single-centre	12	Completed	No
	4.<br>NCT01947101	FMT	CD/UC	Not approved; regulation and support for development provided		Not approved; regulatory submissions ongoing for marketing authorization in 5-10 years		Single-arm, open-label, phase 1	None	Feb 2014	Frequency of AEs	Efficacy in inducing or maintaining remission as per PUCAI	12 to 20	Diagnosis of UC based on clinical symptoms, as well as laboratory and colonoscopic findings, including histology, PUCAI < 35	Induction and maintenance	Institutional grant	US	Single-centre	6	Completed	No

Intervention category	Study ID	Intervention	FDA/EMA approval (if applicable)					Study design	Comparator (if applicable)	Initial enrolment	Primary outcome measure(s)	Secondary outcome measure (s)	Age	Population characteristics	Treatment phase	Sponsor	Location	Single- vs multi-centre	Target or actual sample size	Recruitment status	Results (yes/no)
			Indication	FDA – adult	FDA – paediatric	EMA – adult	EMA – paediatric														
				*Rebyota (rectal formulation) approved on Nov 30, 2022										[mild activity] History of steroid dependency							
	5. NCT02108821	FMT	CD/UC	Not approved; regulation and support for development provided<br><br>*Rebyota (rectal formulation) approved on Nov 30, 2022		Not approved; regulatory submissions ongoing for marketing authorization in 5-10 years		Single-arm, open-label, phase 1	None	Mar 2014	Frequency of AEs	Efficacy in inducing or maintaining remission as per PUCAI or PCDAI	2 to 22	IBD patients with a flare due to failure of current therapy and have to undergo EGD and colonoscopy for restaging the disease and escalation of therapy<br>PUCAI<65;<br>PCDAI<40	NA	Institutional grant	US	Single-centre	23	Completed	Yes
	6. NCT03267238	FMT	CD/UC	Not approved; regulation and support for development provided<br><br>*Rebyota (rectal formulation) approved on Nov 30, 2022		Not approved; regulatory submissions ongoing for marketing authorization in 5-10 years		Single-arm, open-label, early phase 1	None	Jun 2017	TEAEs	1. Microbial diversity<br>2. Microbial composition<br>3. Microbial function<br>4. FCal	7+	1. Established CD diagnosis with one or more of the following: Treated with steroids, IMs or biological therapy for at least a month	NA	Institutional grant	US	Single-centre	9	Completed	No
	7. NCT03194529	FMT	CD/UC	Not approved; regulation and support for development provided<br><br>*Rebyota (rectal formulation) approved on Nov 30, 2022		Not approved; regulatory submissions ongoing for marketing authorization in 5-10 years		Single-arm, open-label, phase 1/2	None	Oct 2017	TEAEs	1. PCDAI<br>2. Changes in gut microbiome	7 to 21	1. CD diagnosis<br>2. Remission of disease as per PCDAI<10<br>3. Needs upper GI Endoscopy	Maintenance	Institutional grant	US	Single-centre	9	Completed	No
Antibiotic and antifungal therapy (RCTs)	1. NCT02033408	Methylprednisolone + vancomycin/ amoxycillin/ metronidazole/ doxycycline/ ciprofloxacin)	Non-IBD indications	Approved for non-IBD indications		Approved for non-IBD indications		Parallel, single-blind	Steroids only – methylprednisolone/metronidazole<br>Open arm – antibiotics OR FMT	Jan 2014	Total PUCAI	1. Remission as per PUCAI<10<br>2. PUCAI<35<br>3. Second line therapy<br>4. Rate of steroid<br>5. Need for admission<br>6. FCal<br>7. Rate of gastrointestinal carriage of resistant organisms<br>8. Change in microbiome pattern<br>9. Rate of *C. difficile* infection	2-75	1. UC diagnosis with admission for IV steroid therapy<br>2. PUCAI at least 65 at admission and >45 at baseline	Induction	Institutional grant	Canada, Finland, Israel, Italy, Poland, Spain	Multi-centre	28	Completed	Yes
	2. NCT01596894	Azithromycin + metronidazole<br><br>*Antibiotic Therapy*	Non-IBD indications	Approved for non-IBD indications		Approved for non-IBD indications		Parallel, single-blind, phase 4	Metronidazole	Oct 2012	Clinical response as per PCDAI	1. CRP<br>2. FCal<br>3. Remission	5 - 17	1. Mild to moderate active CD of less than 3 years with PCDAI 10 – 40 incl.<br>2. Colonic and/or ileal involvement defined as L1, L2, L3 and may have L4a<br>3. CRP>0.6 mg/dL<br>4. Negative *C. difficile* toxin	Induction	Individual	Israel	Multi-centre	73	Completed	Yes

Intervention category	Study ID	Intervention	FDA/EMA approval (if applicable)					Study design	Comparator (if applicable)	Initial enrolment	Primary outcome measure(s)	Secondary outcome measure (s)	Age	Population characteristics	Treatment phase	Sponsor	Location	Single- vs multi-centre	Target or actual sample size	Recruitment status	Results (yes/no)
			Indication	FDA – adult	FDA – paediatric	EMA – adult	EMA – paediatric														
	3. NCT03794765	Ceftriaxone, metronidazole<br><br>*Antibiotic Therapy*	Non-IBD indications	Approved for non-IBD indications		Approved for non-IBD indications		Parallel, triple-blind, phase 2	Placebo	Apr 2019	Clinical response as per Oxford's criteria	1. Second-line therapy<br>2. Colectomy<br>3. Mortality<br>4. Duration of hospital stay<br>5. Serum CRP<br>6. MS<br>7. FCal	13+	Acute severe UC	Induction	Institutional grant	India	Single-centre	50	Completed	No
	4. NCT03476317	Fluconazole<br><br>*Antifungal Therapy*	Non-IBD indications	Approved for non-IBD indications		Approved for non-IBD indications		Parallel, quadruple-blind, phase 2	Placebo	Jul 2018	1. Change in FCal<br>2. Change in PCDAI	1. CRP<br>2. Safety and tolerability via side effects and/or AEs	6+	1. Active CD or IBD-U as per PCDAI > 30<br>2. CRP > 15 mg/L or FCal > 350 µg/g<br>3. Primary nonresponse or loss of response for immunomodulators or anti-TNF after at least 8 weeks of treatment	NA	Government agency	US	Single-centre	10	Completed	Yes
Procedural or tissue transfer therapy (non-RCTs)	1. NCT02150551	Allogeneic bone marrow-derived mesenchymal stromal cells	CD/non-IBD indications	Approved for non-IBD indications outside the US (Canada, 2012; India, 2016; Japan, 2015, 2018; New Zealand, 2012; South Korea, 2011, 2014,)<br><br>*Adipose tissue-derived mesenchymal stromal cells are approved for Crohn's fistula (South Korea, 2012) and complex perianal fistulas in CD (Europe, 2018)		Not approved<br><br>* Adipose tissue-derived mesenchymal stromal cells are approved for complex perianal fistulas in CD (2018)		Single-arm, open-label, phase 1	None	Jun 2018	AEs, SAEs, and/or early treatment discontinuations	1. Clinical response as per PCDAI<br>2. Improvement in lab parameters – CRP, FCal, anti-HLA antibodies, viral-specific T-cell activity	12-22 years	Patients with moderate-severely active CD or UC and documented active disease on flexible sigmoidoscopy, colonoscopy or MRE within the preceding 2 months;<br>Patients who have failed or are intolerant of biologic therapy	NA	Individual	US	Single-centre	1	Completed	Yes
	2. NCT03803917	Injection with freshly collected autologous adipose tissue	CD/UC	Not approved		Not approved		Single-arm, open-label	None	Mar 2015	Clinical healing of the fistula(e)	1. Ceased or reduced fistula secretion<br>2. Complications to the treatment	0 to 65+	1. Clinical diagnosis of CD<br>2. Presence of complex CD fistulae<br>3. Fistulae refractory to standard surgical intervention, including long-term seton and regular curettage, and/or medical treatment	NA	Institutional grant	Denmark	Single-centre	21	Completed	Yes
	3. NCT03449069	Autologous mesenchymal stromal cell coated fistula plug	CD	Not approved		Nor approved		Single-arm, open-label, phase 1	None	Jul 2018	Number of participants with treatment-related adverse events (safety and toxicity)	Response to treatment<br>Radiographic response to treatment	12-17 years	CD with single or multiple draining complex fistulae<br>Concurrent clinical therapies<br>Colonoscopy in the last year<br>No contraindications to MR evaluations<br>Failed standard therapy including ani-TNFs	NA	Institutional grant	USA	Single-centre	1	Completed	No
Miscellaneous therapy (RCTs)	1. NCT00715117	Naltrexone	Non-IBD indications	Approved for non-IBD indications		Approved for non-IBD indications		Cross-over, quadruple-	Placebo	Jul 2008	AEs	1. PCDAI<br>2. IMPACT III	6 - 17	CD diagnosis with PCDAI of at least 31	NA	Institutional grant	US	Single-centre	14	Completed	Yes

Intervention category	Study ID	Intervention	FDA/EMA approval (if applicable)					Study design	Comparator (if applicable)	Initial enrolment	Primary outcome measure(s)	Secondary outcome measure (s)	Age	Population characteristics	Treatment phase	Sponsor	Location	Single- vs multi-centre	Target or actual sample size	Recruitment status	Results (yes/no)
			Indication	FDA – adult	FDA – paediatric	EMA – adult	EMA – paediatric														
		Opioid Antagonist						blind, phase 2													
	2. NCT00109473	Growth hormone<br><br>(Nutropin AQ)	Non-IBD indications	Approved for non-IBD indications		Approved for non-IBD indications		Parallel, open-label, phase 2	Corticosteroids	Apr 2005	CDHIS	1. IMPACT III score<br>2. PCDAI<br>3. Total corticosteroid use<br>4. CDEIS<br>5. Height velocity	5+	1. Moderate-severe CD diagnosis with ileo-colonic involvement<br>2. On budesonide or prednisone starting dose or stable on AZA/6-MP	Induction and maintenance	Institutional grant	US	Single-centre	22	Completed	Yes
	3. NCT00463151	Rebamipide (60, 150, 300 mg)<br><br>*Prostaglandins and epidermal growth factor stimulator*	Non-IBD indications	Not FDA or EMA approved. Other approvals:<br>1. Japan – 2011<br>*(Gastric ulcers and gastritis)*<br>2. Russia – 12 Sep 2012<br>*(Gastroprotective agent)*<br>3. India – 29 Jul 2004<br>*(Gastric intestinal ulcers in adults)*<br>4. South Korea – 25 May 2016 (registered)				Parallel, quadruple-blind, phase 2	Placebo	Jun 2007	Clinical improvement rate – decrease of DAI	1. Clinical remission as per DAI<br>2. Mean change in total DAI score<br>3. Improvement in DAI sub-score<br>4. Mean change in total endoscopic index (EI) score	16+	1. Active UC patients with insufficient response to oral ASA<br>2. Major lesions between the sigmoid and rectum on colonoscopy	Induction	Industry	Japan	Multi-centre	124	Completed	Yes
	4. NCT00259558	Retarded release Phosphatidylcholine<br><br>*Phospholipid*	Non-IBD indications	Summary report for clinical use under 503B as a Bulk Drug Substance submitted in Oct 2021		Not authorized by the EMA		Parallel, double-blind, phase 2	PC (different dose groups)	Apr 2003	Change in clinical index (Rachmilewitz)	1. Clinical remission (CAI<3)<br>2. Clinical response (CAI improvement >50%)<br>3. Histology<br>4. QoL<br>5. Endoscopic response (EAI improvement >50%)	16 – 90	Chronic active ulcerative pancolitis with clinical index rachmilewitz and endoscopic index > 7 each	NA	Institutional grant	Germany	Single-centre	60	Completed	Yes
	5. NCT00259545	Retarded release Phosphatidylcholine<br><br>*Phospholipid*	Non-IBD indications	Summary report for clinical use under 503B as a Bulk Drug Substance submitted in Oct 2021		Not authorized by the EMA		Parallel, double-blind, phase 2	Placebo	Feb 2003	Complete steroid reduction	1. Clinical remission<br>2. QoL<br>3. Endoscopic indices<br>4. Histological scor	16 – 90	Active and steroid-dependent UC	Induction	Institutional grant	Germany	Single-centre	60	Completed	Yes
	6. NCT05684484	Roflumilast	Non-IBD indications	Approved for non-IBD indications		Approved for non-IBD indications		Parallel, open-label, phase 4	CS +immune suppressive +amino salicylic acid	Feb 2023	Clinical improvement in MS	Changes in serum levels of the measured biochemical parameters (TNF-α, STAT3, Caspase-3)	15-80	Mild-moderate UC	Induction	Institutional grant	Egypt	Single-centre	52	Completed	No
Miscellaneous therapy (non-RCTs)	1. NCT01991314	Ferrous sulphate<br><br>*Iron supplements*	CD/UC	Not approved; dietary supplements do not need FDA approval		Present in the Directive 2002/46/EC on the approximation of laws relating to food supplements)		Single-arm, open-label, phase 4	None	Dec 2011	Hb concentration	1. AEs<br>2. CALPR<br>3. SIBDQ<br>4. HADS-A<br>5. MFI<br>6. PSQ-G	13 to 80	IBD patients with proven iron deficiency anaemia on World Health Organisation (WHO)criteria Patients aged 13 – 18 will be considered adolescents, and aged >18 as adults	NA	Institutional grant	UK	Single-centre	90	Completed	Yes

Intervention category	Study ID	Intervention	FDA/EMA approval (if applicable)					Study design	Comparator (if applicable)	Initial enrolment	Primary outcome measure(s)	Secondary outcome measure (s)	Age	Population characteristics	Treatment phase	Sponsor	Location	Single- vs multi-centre	Target or actual sample size	Recruitment status	Results (yes/no)
			Indication	FDA – adult	FDA – paediatric	EMA – adult	EMA – paediatric														
	2. NCT02442960	SA100<br><br>*Herbal treatment (high dose)*	UC	Not approved		Not approved		Parallel, open-label, phase 1	SA100 low dose	Dec 2014	Safety and tolerability via clinical and laboratory AEs	1. Total MS<br>2. SIBDQ<br>3. CRP<br>4. ESR	13 to 75	Mild, moderate and severe UC; MS of 3-12 at screening;<br>Active disease refractory to, intolerant to, 5-ASA, steroids, small molecule, IMs, and biologics or steroid-dependent;<br>PGA Mayo subscore and Mayo endoscopic subscore of at least 1 at screening	Induction	Institutional grant	US	Single-centre	15	Completed	Yes
	3. NCT06777706	Qing Dai<br><br>*Chinese traditional (herbal) medicine*	UC	Not approved		Not approved		Single-arm, open-label	None	Jan 2023	Clinical remission as per PUCAI	1. Clinical response<br>2. Calprotectin <250 mcg/g<br>3. Rate of calprotectin decrease<br>4. Clinical remission at week 3<br>5. Clinical response at week 3<br>6.	4 to 17	Mild to moderate UC	Induction	Institutional grant	Israel	Multi-centre	20	Completed	No
	4. NCT02745678	Thermosensitive gel rectal formulation	CD/UC	Unknown (formulation/brand name not specified in study)		Unknown (formulation/brand name not specified in study)		Single-arm, open-label, phase 1	None	Jan 2016	Safety and efficacy as per the mMS	None provided	8+	Confirmed diagnosis of active, mild to moderate ulcerative proctitis or proctosigmoiditis extending no further than 40 cm from the anal verge (baseline MS of 5-10)	NA	Institutional grant	US	Single-centre	3	Completed	No

Data extracted from ClinicalTrials.gov and ClinicalTrialsRegister.eu.

Abbreviations: AE, adverse event; ADA, adalimumab; AVA, anti-vedolizumab antibody; 5-ASA, 5-aminosalicylates; AUC, area under plasma concentration curve; AZA, azathioprine; CAI, Clinical Activity Index; CD, Crohn's disease; CDAI, Crohn's Disease Activity Index; CDED, Crohn's disease exclusion diet; CDEIS, Crohn's Disease Endoscopic Index of Severity; CDHIS, Crohn's Disease Histologic Index of Severity; Cmax, maximum plasma concentration; CRP, C-reactive protein; CS, corticosteroid; DAI, Disease Activity Index; EEN, exclusive enteral nutrition; EIMs, extraintestinal manifestations; ESR, erythrocyte sedimentation rate; FMT, faecal microbiota transplant; HADS-A, Hospital Anxiety and Depression Score – Anxiety; IBD, inflammatory bowel disease; IBD-U, inflammatory bowel disease - unclassified; IBDQ, Inflammatory Bowel Disease Questionnaire; IL, interleukin; IMM, immunosuppressive; IOIBD, International Organization for the Study of Inflammatory Bowel Disease; MAdCAM-1, mucosal addressin cell adhesion molecule-1; MIF, Multidimensional Fatigue Inventory; mos, months; 6-MP, 6-mercaptopurine; MS, Mayo score; mMS, modified Mayo score; MTX, methotrexate; NA, not available; PCDAI, Paediatric Crohn's Disease Activity Index; PEN, partial enteral nutrition; PGA, Physician Global Assessment; PK, pharmacokinetics; PSQ-G, Perceived Stress Questionnaire; pMS, partial Mayo score; PUCAI, Paediatric Ulcerative Colitis Activity Index; QoL, quality of life; RCT, randomised controlled trial; SAE, serious adverse event; SF-36, Short Form 36; SIBDQ, Short Inflammatory Bowel Disease Questionnaire; Tmax, time to max. plasma concentration; TNF, tumour necrosis factor; UC, ulcerative colitis; UK, United Kingdom; US, United States; yrs, years.

Supplementary Table 3: Characteristics of Active/Ongoing Trials in Paediatric IBD

Intervention category	Study ID	IBD	Intervention	Study design	Comparator (if applicable)	Age	Start date	Expected/ Actual enrolment
**Biologic agents (RCTs)**	1. NCT07158242	UC	Afimkibart	Parallel, quadruple-blind, phase 3	Different dose regimen	2-17 years	15-Apr-2026	100
	2. NCT07184931	CD	Duvakitug	Parallel, quadruple-blind, phase 3	Placebo	16-80 years	1-Oct-2025	980
	3. NCT07184996	UC	Duvakitug	Parallel, quadruple-blind, phase 3	Placebo	16-80 years	8-Oct-2025	980
	4. NCT07185009	UC	Duvakitug	Parallel, quadruple-blind, phase 3	Placebo	16-80 years	16-Jan-2026	671
	5. NCT07184944	CD	Duvakitug	Parallel, quadruple-blind, phase 3	Placebo	16-80 years	22-Jan-2026	751
	6. NCT03596645	UC	Golimumab	Parallel, open-label, phase 3	Infliximab	2-17 years	29-Oct-2018	84
	7. NCT06260163	UC	Guselkumab	Parallel, double-blind, phase 3	Different dose regimen	2-17 years	19-Jan-2024	112
	8. NCT05923073	CD	Guselkumab	Parallel, triple-blind, phase 3	Placebo	2-17 years	13-Mar-2024	120
	9. NCT07196748	UC	Icotrokinra	Parallel, double-blind, phase 3	Placebo	12+ years	1-Oct-2025	882
	10. EudraCT: 2012-000645-13	CD	Infliximab	Open-label, phase 4	Different dose regimen	1-17 years	17-Oct-2012	90
	11. NCT04835506	IBD	Infliximab	Parallel, open-label, phase 4	Different dose regimen	16+ years	01-Nov-2021	124
	12. NCT04999228	UC	Infliximab	Parallel, single-blind, phase 4	Corticosteroid	6-18 years	01-Nov-2021	40
	13. NCT05043870	CD	Infliximab	Parallel, single-blind, phase 4	Infliximab + immunomodulator vs infliximab only	6-18 years	10-Oct-2022	128
	14. NCT06839820	IBD	Infliximab	Cross-over, open-label	Different dose regimen	1-17 years	01-Feb-2025	60
	15. NCT04646187	IBD	Infliximab + adalimumab	Parallel, open-label, phase 4	Standard dosing	12-25 years	11-Mar-2021	148
	16. NCT07424040	CD	Infliximab + azathioprine therapy	Parallel, open-label	Infliximab	6-18 years	1-Mar-2026	154
	17. NCT07314606	IBD	Infliximab + modified CDED	Parallel, open-label	Infliximab	9-17 years	22-Apr-2026	140
	18. NCT03105102	CD	Risankizumab	Parallel, quadruple-blind, phase 3	Placebo	16-80 years	09-Apr-2018	1336
	19. NCT03398135	UC	Risankizumab	Parallel, triple-blind, phase 3	Placebo	16-80 years	28-Aug-2018	1242

Intervention category	Study ID	IBD	Intervention	Study design	Comparator (if applicable)	Age	Start date	Expected/ Actual enrolment
	20. NCT05995353	CD	Risankizumab	Sequential, quadruple-blind, phase 3	Different dose regimen	2-17 years	11-Dec-2023	110
	21. NCT07071519	UC	Risankizumab	Sequential, double-blind, phase 3	Different dose regimen	2-17 years	01-Aug-2025	120
	22. NCT06052059	UC	Tulisokibart	Parallel, quadruple-blind, phase 3	Placebo	16-80 years	25-Oct-2023	1020
	23. NCT04779320	CD	Vedolizumab	Parallel, quadruple-blind, phase 3	Different dose regimen	2-17 years	10-Feb-2022	120
	24. NCT06405087	IBD	Vedolizumab	Parallel, open-label, phase 3	N/A	2-17 years	27-Oct-2025	70
	25. NCT06614387	UC	Vedolizumab	Parallel, single-blind, phase 3	Infliximab	6-17 years	01-Oct-2024	60
**Biologic agents (non-RCTs)**	1. NCT06742606	IBD	Adalimumab	Single-arm, open-label, early phase 1	Ustekinumab	1 month-16 years	29-Oct-2024	40
	2. NCT06663332	IBD	Guselkumab	Parallel, open-label, phase 3	Different dose regimen	> 3 years	29-Oct-2024	196
	3. EudraCT: 2008-005237-30	IBD	Infliximab	Open-label, phase 4	IBD medications other than infliximab	2-17 years	01-Dec-2009	6000
	4. NCT05660746	CD	Infliximab	Parallel, open-label, phase 2/3	Different dose regimen	6-22 years	01-Jul-2023	180
	5. NCT05552287	CD	Infliximab	Single-arm, open-label, phase 4	N/A	3-15 years	02-Aug-2023	50
	6. NCT05906576	CD	Infliximab	Single-arm, open-label, phase 4	N/A	6-17 years	21-Sep-2023	30
	7. NCT04844606	IBD	Mirikizumab	Parallel, open-label, phase 3	Different dose regimen	2-19 years	26-May-2021	150
	8. NCT05509777	CD	Mirikizumab	Parallel, open-label, phase 3	Different dose regimen	2-17 years	13-Mar-2024	90
	9. EudraCT: 2004-005016-93	CD	Polyglobin	Open-label pilot study	N/A	2-17 years	01-Apr-2005	15
	10. NCT06298188	CD	Risankizumab	Single-arm, open-label	N/A	6-18 years	31-Oct-2024	90
	11. NCT05092269	IBD	Ustekinumab	Single-arm, open-label, phase 3	N/A	2-17 years	18-Oct-2021	159
	12. NCT07529236	IBD	Ustekinumab	Single-arm, open-label	N/A	6-17 years	20-Apr-2026	40
	13. NCT05442567	IBD	Vedolizumab	Parallel, open-label, phase 3	Different dose regimen	2+ years	16-May-2023	240
	14. NCT06100289	IBD	Vedolizumab	Parallel, open-label, phase 3	Different dose regimen	2-17 years	22-Jan-2025	70
**Small molecule therapy (RCTs)**	1. NCT05535946	UC	Obefazimod	Parallel, triple-blind, phase 3	Placebo	16+ years	16-Jan-2023	1116

Intervention category	Study ID	IBD	Intervention	Study design	Comparator (if applicable)	Age	Start date	Expected/ Actual enrolment
	2. NCT05076175	UC	Ozanimod	Parallel, quadruple-blind, phase 2/3	Different dose regimen	2-17 years	30-May-2022	120
	3. NCT05782907	UC	Upadacitinib	Sequential, quadruple-blind, phase 3	Different dose regimen	2-17 years	06-Nov-2023	122
	4. NCT06332534	CD	Upadacitinib	Sequential, quadruple-blind, phase 3	Different dose regimen	2-17 years	8-Aug-2024	110
**Small molecule therapy (non-RCTs)**	1. NCT03950232	UC	Etrasimod	Single-arm, open-label, phase 3	N/A	16-80 years	05-Sep-2019	896
	2. NCT05287126	UC	Etrasimod	Single-arm, open-label, phase 2	N/A	12-17 years	16-Dec-2022	36
	3. NCT07470879	UC	Etrasimod	Single-arm, open-label, phase 2	N/A	2-11 years	28-May-2026	24
	4. NCT06865417	UC	Filgotinib	Single-arm, open-label, phase 3	N/A	8-17 years	29-Sep-2025	80
	5. NCT05456412	UC	JAK inhibitor	Sequential, open-label	N/A	16-65 years	20-Dec-2022	60
	6. NCT04624230	UC	Tofacitinib	Sequential, open-label, phase 3	N/A	2-17 years	12-Aug-2021	118
	7. NCT03006068	UC	Upadacitinib	Parallel, quadruple-blind, phase 3	Placebo	16-75 years	31-Jan-2017	950
**Corticosteroids and immunosuppressants (RCTs)**	1. EudraCT: 2012-001653-13	UC	Azathioprine	Parallel, double-blind, phase 4	Placebo	2-17 years	10-Jul-2013	130
	2. NCT04314375	UC	Budesonide	Parallel, double-blind, phase 4	Placebo	5-17 years	29-Sep-2023	70
	3. NCT06719622	CD	Budesonide	Parallel, quadruple-blind, phase 2/3	Omeprazole	6-18 years	30-Jul-2024	114
	4. NCT05976802	UC	Budesonide rectal foam	Parallel, double-blind, phase 4	Placebo	5-17 years	Jun-2027	100
	5. EudraCT: 2016-000522-18	CD	Methotrexate	Parallel, open-label, phase 4	Azathioprine, ADA	6-17 years	*Unavailable*	312
**Nutritional and dietary therapy (RCTs)**	1. NCT03847467	IBD	2'-Fucosyllactose	Sequential-quadruple-blind, phase 1/2	Placebo	11-25 years	20-Sep-2019	116
	2. NCT06515210	UC	Acacia Gum (dietary supplement)	Parallel, double-blind	Microcrystalline cellulose, placebo	14+ years	03-Jun-2025	69
	3. NCT07501078	UC	Aquamin (natural mineral supplement)	Parallel, triple-blind, phase 2	Placebo	5-80 years	Jul-2026	52
	4. NCT05615779	IBD	B-fructan diet	Parallel, double-blind	Pectin diet, placebo	6-39 years	01-Aug-2023	600
	5. NCT07671872	CD	Catered anti-inflammatory diet followed by diet guidance	Cross-over, open-label	Diet guidance followed by catered anti-inflammatory diet	16-75 years	Aug-2026	122

Intervention category	Study ID	IBD	Intervention	Study design	Comparator (if applicable)	Age	Start date	Expected/ Actual enrolment
	6. NCT04777656	CD	CDED + standard therapy	Parallel, single-blind, phase 3	Unrestricted diet	6-17 years	26-Sep-2022	120
	7. NCT04999241	UC	EEN	Parallel, single-blind, phase 4	Regular diet	6-18 years	01-Mar-2025	40
	8. NCT06216899	CD	EEN (whole-food blended smoothie)	Parallel, open-label	EEN (conventional formula)	8-21 years	11-Mar-2024	60
	9. NCT07522528	CD	Enteral nutrition	Parallel, open-label	Unrestricted diet	6-18 years	27-Mar-2026	90
	10. NCT05561738	UC	Nicotinamide riboside chloride	Parallel, quadruple-blind	Placebo	Up to 18 years	28-Feb-2024	40
	11. NCT04859088	CD	PEN	Parallel, open-label	Unrestricted diet	16+ years	20-Sep-2021	80
	12. NCT04522271	IBD	Resistant starch	Parallel, quadruple-blind	Placebo	5-17 years	25-Aug-2020	80
	13. NCT04520594	IBD	Resistant starch	Parallel, quadruple-blind	Placebo	5-17 years	03-Mar-2021	100
	14. NCT06301477	IBD	Resistant starch	Parallel, quadruple-blind	Placebo	8-16 years	1-Dec-2024	45
	15. NCT05791487	UC	UC exclusion diet + PEN + oral budesonide	Parallel, single-blind	Oral budesonide + free diet	17-65 years	01-Dec-2023	90
**Nutritional and dietary therapy (non-RCTs)**	1. NCT04513015	IBD	Antioxidant diet	Parallel, open-label	Normal diet	6-80 years	15-Dec-2019	155
	2. NCT07334340	CD	Nordic CDED	Single-arm, open-label	N/A	6-18 years	1-Feb-2026	30
**Microbiological and microbiota interventions (RCTs)**	1. NCT05202990	UC	FMT (oral)	Parallel, open-label, phase 2	FMT (intra-rectal enemas)	8-17 years	1-Jul-2026	26
	2. NCT07318753	IBD	Lactobacillus rhamnosus CALM 607（LR607）	Parallel, quadruple-blind	Placebo	*Unavailable*	1-Jun-2025	300
	3. NCT07296315	UC	MH002 (microbiome-based live biotherapeutic product)	Parallel, quadruple-blind, phase 2	Placebo	16+ years	3-Apr-2026	204
	4. NCT07533890	IBD	Multi-strain probiotics	Cross-over, single-blind	N/A	5-18 years	1-May-2026	40
**Microbiological and microbiota interventions (non-RCTs)**	1. NCT02636517	IBD	FMT	Parallel, open-label, phase 1	N/A	3-21 years	Dec-2015	250
	2. NCT04014413	IBD	FMT	Parallel, open-label	N/A	*Unavailable*	15-Jul-2019	450
	3. NCT05321758	CD	FMT	Parallel, open-label	Immunosuppressive agents	2-16 years	22-Mar-2020	50
	4. NCT05321745	CD	FMT	Single-arm, open-label	N/A	2-16 years	22-Mar-2022	40
	5. NCT05679622	UC	FMT	Single-arm, open-label	N/A	2-16 years	01-Dec-2022	30
	6. NCT07154784	UC	FMT	Single-arm, open-label, phase 2/3	N/A	10-80 years	19-Jul-2023	30
	7. NCT01765439	IBD	VSL#3	Parallel, open-label	N/A	*Unavailable*	Feb-2014	79

Intervention category	Study ID	IBD	Intervention	Study design	Comparator (if applicable)	Age	Start date	Expected/ Actual enrolment
**Antibiotics and antifungal therapy (RCTs)**	1. NCT07646223	UC	Oral vancomycin	Parallel, open-label, phase 2	Conventional treatment	6-15 years	1-Aug-2026	140
**Procedural or tissue transfer therapy (non-RCTs)**	1. NCT00692939	CD	Autologous stem cell transplant	Single-arm, open-label, phase 1/2	Multiple drugs	10-60 years	26-Jun-2012	20
	2. NCT04224558	CD	Autologous stem cell transplant	Single-arm, open-label, phase 1/2	Multiple drugs	13-28 years	15-Nov-2019	15
	3. NCT06986382	CD	Hematopoietic stem cell transplant	Single-arm, open-label, phase 1/2	N/A	2-30 years	01-Jul_2025	14
	4. NCT05003947	IBD	Mesenchymal stem cells	Single-arm, open-label	N/A	*Unavailable*	11-Jun-2022	15
**Miscellaneous therapy (RCTs)**	1. NCT05986136	UC	Dapagliflozin	Parallel, double-blind, phase 2/3	Mesalamine	18 months-60 years	20-Aug-2023	50
**Miscellaneous therapy (non-RCTs)**	1. NCT05218850	UC	Butyrate enema	Single-arm, open-label, phase 2	N/A	7-21 years	29-Jun-2022	10

Data extracted from ClinicalTrials.gov and ClinicalTrialsRegister.eu.

Abbreviations: ADA, adalimumab; CD, Crohn's disease; CDED, Crohn's disease exclusion diet; EEN, exclusive enteral nutrition; FMT, faecal microbiota transplant; IBD, inflammatory bowel disease; IV, intravenous; N/A, not applicable; RCT, randomised controlled trial; SC, subcutaneous; SOC, standard of care; TDM, therapeutic drug monitoring; PEN, partial enteral nutrition; UC, ulcerative colitis
